# Genome-wide identification of *CNGC* genes in Chinese jujube (*Ziziphus jujuba* Mill.) and *ZjCNGC2* mediated signalling cascades in response to cold stress

**DOI:** 10.1186/s12864-020-6601-5

**Published:** 2020-03-02

**Authors:** Lixin Wang, Min Li, Zhiguo Liu, Li Dai, Mengling Zhang, Lili Wang, Jin Zhao, Mengjun Liu

**Affiliations:** 1 College of Horticulture, Hebei Agricultural University, Baoding, 071001 Hebei China; 2 Research Center of Chinese Jujube, Hebei Agricultural University, Baoding, 071001 Hebei China; 3 College of Life Science, Hebei Agricultural University, Baoding, 071001 Hebei China; 4 Beijing Collaborative Innovation Center for Eco-environmental Improvement with Forestry and Fruit Trees, Beijing, 100000 China

**Keywords:** Chinese jujube, CNGCs, Phylogeny, Stresses, Signalling

## Abstract

**Backgrounds:**

Cyclic nucleotide gated channels (CNGCs) play multifaceted roles in plant physiological processes, especially with respect to signalling processes, plant development, and responses to environmental stresses. However, little information is known about the CNGC family in the large cosmopolitan family *Rhamnaceae*, which has strong tolerance to biotic and abiotic stresses.

**Results:**

In the current study, a total of 15 *ZjCNGCs* which located on 7 chromosomes were firstly identified in Chinese jujube (*Ziziphus jujuba* Mill.), the most important species of *Rhamnaceae* in terms of economic and ecological values. Phylogenetic analysis showed that these *ZjCNGCs* could be classified into four groups, *ZjCNGC12* belonged to group IVA, and *ZjCNGC13*, *14*, *15* belonged to group IVB. In addition, the paralogous and orthologous homology duplication of *ZjCNGC15* occurred during the evolutionary process. The characteristics of *ZjCNGCs* regarding to exon-intron numbers and post-translational modifications showed diversified structures and functions. Motif composition and protein sequence analysis revealed that the phosphate-binding cassette and hinge regions were conserved among ZjCNGCs. Prediction of the *cis*-acting regulatory elements and expression profiles by real-time quantitative PCR analysis showed that some of the *ZjCNGCs* responded to environmental changes, especially *ZjCNGC2*, which was significantly downregulated in response to cold stress, and *ZjCNGC4* was highly induced in response to cold, salt and alkaline stresses. *ZjCNGC13* and *14* were highly induced in the phytoplasma-resistant cultivar and downregulated in the susceptible cultivar. Furthermore, *ZjCNGC2* could be regulated by cAMP treatment, microtubule changes and interact with ZjMAPKK4, which suggested that cAMP and microtubule might play important roles in *ZjCNGC2* mediated *ZjMAPKK4* signalling transduction involved in cold stress.

**Conclusions:**

The identification and classification analysis of *ZjCNGCs* were firstly reported, and some key individual *ZjCNGCs* might play essential roles in the response to biotic and abiotic stresses, especially *ZjCNGC2* mediated *ZjMAPKK4* signalling transduction involved in cold stress. This systematic analysis could provide important information for further functional characterization of *ZjCNGCs* with the aim of breeding stress-resistant cultivars.

## Background

Calcium (Ca^2+^), as an important second messenger, functions as a signalling molecule in many biological processes, including plant development, pollen fertility and tip growth, various biotic and abiotic stresses [1, 2]. The Ca^2+^ signal is modulated by the intimate interplay of channels and transporters as a so-called ‘on’ and ‘off’ mechanisms [3]. In *Arabidopsis*, five families of Ca^2+^-permeable channels have been identified, including 20 members of cyclic nucleotide gated channels (CNGCs) [2, 4]. In the plant kingdom, CNGCs are cation transport channels that enable sodium, calcium and potassium to cross cellular membranes. CNGCs localize on the cell membrane or different organelle membranes and can be regulated by inside second messengers, such as cyclic nucleotide monophosphates (3′,5′-cAMP and 3′,5′-cGMP) and Ca^2+^/calmodulin (CaM) [5–9]. Plant CNGCs can be divided into groups I, II, III, IVA, and IVB according to their phylogenetic relationship and are mainly characterized by six hexa-transmembrane (TM) domains, a pore-forming region between the fifth and sixth TM domains, a calmodulin-binding domain (CaMBD) and a cytosolic C-terminus containing a cyclic nucleotide-binding domain (CNBD). Among these structures, the CNBD domain, which is the most conserved domain, contains a phosphate-binding cassette (PBC) that can bind to cNMP and a hinge region that promotes ligand binding efficacy and selectivity [10–12]. These special properties are necessary for CNGC functions and are widely used to identify CNGCs in plant systems.

With informatics studies, the CNGC gene family has been identified in various plants; 20 members have been identified in *Arabidopsis* [4], 16 in rice [13], 18 in tomato [14], 21 in pear [15], 47 in wheat [16], 35 in tobacco [17], 12 in maize [18] and 26 in *Brassica oleracea* [19]. However, little is known about the biological information of the CNGC family in Chinese jujube.

In numerous physiological and molecular processes, such as plant development, symbiosis, circadian rhythm and in response to environmental stresses, including cold, heat and salt [20, 21], plant CNGCs play important roles in signal transduction. For example, *MdCNGC1* was identified in the apple genome which was highly induced by *Botryosphaeria dothidea* infection. With the transformation to tobacco, *MdCNGC1* negatively regulates resistance to bacterial and fungal pathogens [22]. In *Arabidopsis*, *AtCNGC7* and *AtCNGC8* are essential for male reproductive fertility, and *AtCNGC16* and *AtCNGC18* participate in pollen development [23–25]. *AtCNGC2* is involved in jasmonic acid (JA)-induced apoplastic Ca^2+^ influx, which is further activated by cAMP [26], and *AtCNGC2*, similar to *CNGC20* and *CNGC12*, can bind CaM via its IQ domain [27]. In addition, the *AtCNGC2* orthologues (*Arabidopsis Defense No Death* 1) in potato and tomato negatively regulate resistance to late blight and powdery mildew, resulting in dwarfing and necrosis in tomato but not in potato [28]. In wheat, *TaCNGC14* and *TaCNGC16* play a negative role in resistance against pathogens [16]. In tomato, *SlCNGC7* and *SlCNGC14* play a negative role in drought tolerance [29]. However, the biological function of *ZjCNGCs* is still elusive.

Chinese jujube (*Ziziphus jujuba* Mill.) is the most important economic species and multipurpose fruit tree in the family of *Rhamnaceae*. It can adapt to multiple abiotic stresses, especially alkaline and salinity. Moreover, the whole genome of this plant species has been sequenced [30, 31], providing a foundation for us to identify the CNGC family in Chinese jujube. In the current study, the phylogenetic analysis, gene structure and conserved motifs of *CNGCs* in Chinese jujube (*ZjCNGCs*) were predicted. Furthermore, the expression profiles of these *CNGCs* in response to abiotic and abiotic stresses were investigated by real-time quantitative PCR (qPCR), and the *ZjCNGC2* mediated signalling cascades involved in cold stress were studied.

## Results

### Identification of *CNGC* genes in the jujube genome

With 20 *Arabidopsis* and 21 pear CNGC protein sequences as queries to search against the jujube genome, 15 *ZjCNGC*s were retrieved as potential candidate genes. The domain composition analysis demonstrated that all of the candidate genes were conformed as CNGC proteins (see additional files S1 and S2). Among them, 14 ZjCNGC protein sequences contained both an ion transporter (ITP) domain and a cyclic nucleotide-binding domain (CNBD) [32], which are bona fide CNGC genes. However, ZjCNGC3 only had an ITP domain based on SMART analysis, and the amino sequence of ZjCNGC3 was significantly shorter than that of other members (see additional files S3 and S4), but the annotation of this gene was cyclic nucleotide-gated ion channel 1-like in the NCBI database. Thus, with comprehensive analysis, we suspected that a mistake might have occurred during sequencing, and ZjCNGC3 should actually be a full-length CNGC protein. Finally, a total of 15 *ZjCNGCs* were identified, and this number was smaller than that the number of *CNGCs* identified in *Arabidopsis* and pear. In addition, the identified *ZjCNGC* genes were designated as *ZjCNGC1* to *ZjCNGC15* based on their subfamily classification (Table 1).

**Table 1:** Identification of *CNGC* genes in *Ziziphus jujuba* Mill. (*ZjCNGCs*)

Group	Gene name	Gene ID	Refseq ID	chr	Location	Nucleotidelength (bp)	CDS (bp)	Exon count	Amino Acids Length (aa)	MW (Da)	pI	Stablity	Subcellular localization
I	ZjCNGC1	LOC107414602	XP_015878232.1	3	23,826,933–23,831,489	4557	2148	7	715	82,819.54	9.06	unstable	Cell membrane
	ZjCNGC2	LOC107423657	XP_015888739.1	8	163,003–167,777	4775	2184	8	727	83,314.34	9.08	stable	Cell membrane
	ZjCNGC3	LOC107431258	XP_024934717.1	11	16,971,256–16,961,947	9310	1755	7	584	66,486.95	7.83	stable	Cell membrane
II	ZjCNGC4	LOC107426524	XP_015892210.1	1	26,183,077–26,194,331	11,255	2226	7	741	84,158.52	9.11	unstable	Cell membrane
	ZjCNGC5	LOC107405533	XP_015868088.1	1	39,990,236–39,994,471	4236	2232	7	743	86,211.81	9.39	unstable	Cell membrane
III	ZjCNGC6	LOC107415600	XP_024930943.1	1	8,299,989-8,307,148	7160	1998	7	666	77,466.88	9.41	unstable	Cell membrane
	ZjCNGC7	LOC107426320	XP_015891945.1	1	26,117,668-26,123,235	5568	2133	7	710	81,412.7	9.22	unstable	Cell membrane
	ZjCNGC8	LOC107411379	XP_015874444.1	2	14,942,045-14,947,333	5289	2118	7	705	80,943.8	8.96	unstable	Cell membrane
	ZjCNGC9	LOC107415358	XP_015879150.1	4	3,050,161–3,053,501	3341	2142	6	713	82,218.49	8.64	unstable	Cell membrane
	ZjCNGC10	LOC107429923	XP_015896181.1	11	1,700,846-1,705,676	4831	2199	7	732	84,297.21	8.4	unstable	Cell membrane
	ZjCNGC11	LOC107407431	XP_015870200.1	un	2549–6935	4387	2091	6	696	80,262.42	9.02	unstable	Cell membrane
IVA	ZjCNGC12	LOC107419549	XP_024930017.1	5	28,975,907–28,985,219	9313	2361	12	786	89,765.87	9.3	unstable	Cell membrane
IVB	ZjCNGC13	LOC107430493	XP_015896826.1	1	31,671,028–31,677,305	6278	2082	7	693	79,792.65	8.94	unstable	Cell membrane
	ZjCNGC14	LOC107414103	XP_015877685.1	3	19,344,690–19,348,278	3589	2094	8	697	80,568.7	9.37	unstable	Cell membrane
	ZjCNGC15	LOC107415943	XP_015879866.1	4	8,088,879–8,093,917	5039	2142	9	713	81,866.41	9.57	unstable	Cell membrane

The detailed physiological and biochemical properties of the *ZjCNGC* genes are shown in Table 1. The *ZjCNGCs* were located on 7 chromosomes (Chr), including Chr 1, 2, 3, 4, 5, 8 and 11 although *ZjCNGC11* could not match to a corresponding chromosome. In addition, five of the *ZjCNGC* genes were located on chromosome 1. The CDS length ranged from 1755 bp (*ZjCNGC3*) to 2361 bp (*ZjCNGC12*), with an average of 2127 bp. The length of amino acids ranged from 584 to 786 aa with an average of 708 aa. The predicted molecular weight (Mw) of these proteins ranged from 66.49 to 89.77 kDa, and the theoretical isoelectric point (pI) ranged from 7.83 (*ZjCNGC3*) to 9.57 (*ZjCNGC15*). Moreover, according to the instability index (II), only two proteins were stable in test tubes, namely, ZjCNGC2 and ZjCNGC3. The subcellular localization analysis of all the ZjCNGCs indicated that all of them were located in the cell membrane.

### Phylogenetic analysis of ZjCNGCs

**Fig. 1:**
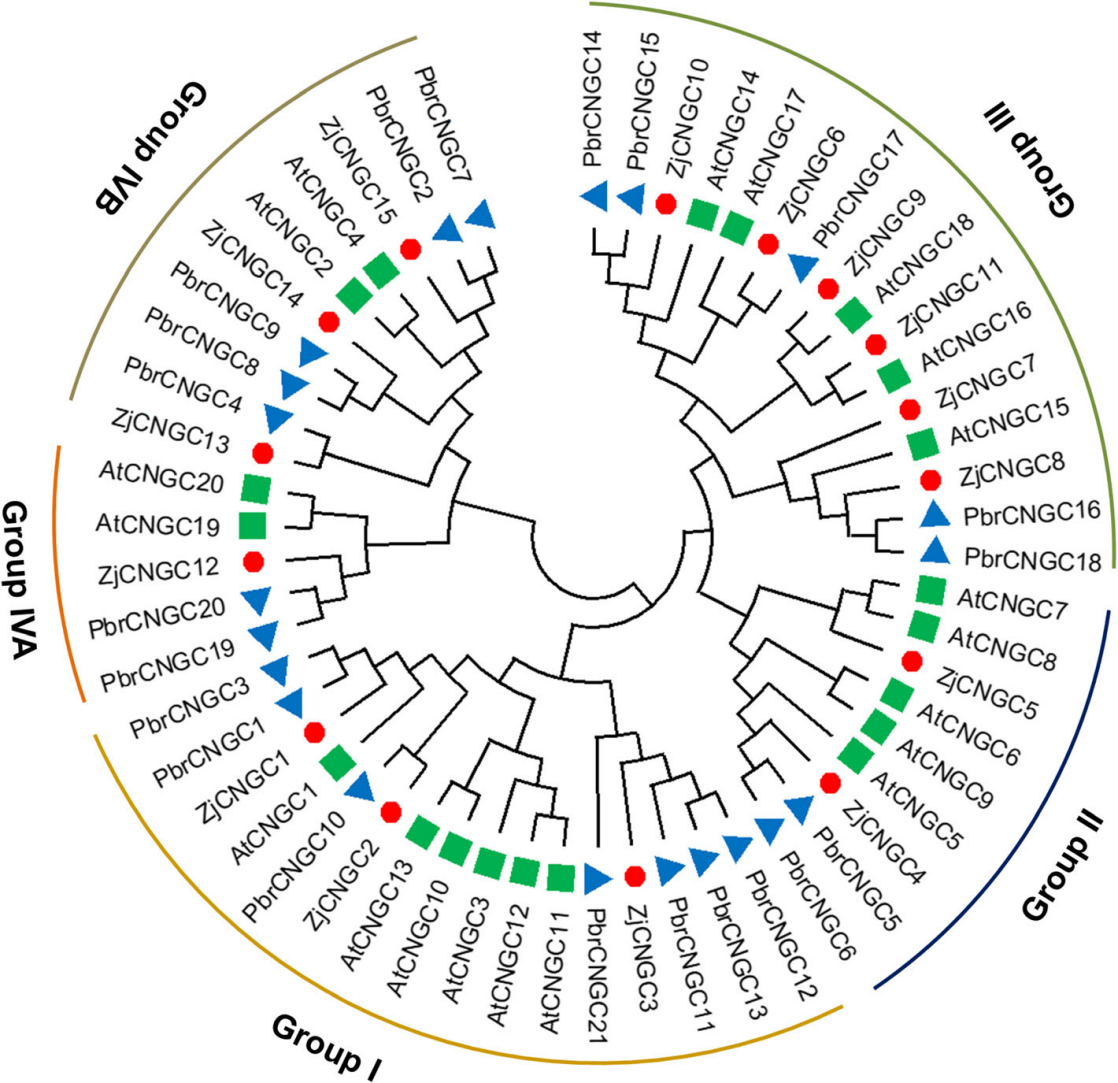
Phylogenetic analysis of the protein sequences of ZjCNGCs, AtCNGCs and PbrCNGCs. MEGA 6.0 was used to construct the phylogenetic tree with the neighbour-joining (NJ) method, and 1000 bootstrap replications were performed to show the reliability. The CNGCs from jujube, pear and *Arabidopsis* were distinguished with different shapes and colours

To analyse the relationship among ZjCNGC proteins, a phylogenetic tree was generated by using the available full-length amino acid sequences of jujube, *Arabidopsis* and pear CNGCs. As shown in Fig. 1, all the proteins could be clustered into four groups as described by Mäser et al. (2001) [4]. Group IV could be divided into another two subgroups (group IVA and group IVB). For each group of AtCNGCs and PbrCNGCs, the corresponding homologous genes were found in jujube, and the number of genes identified was different among groups. Among them, six members of ZjCNGCs (6–11) were clustered into group III, which formed the largest group, and the number of members in group III was basically similar to those in pear and *Arabidopsis*. In addition, group I contained three members (ZjCNGC1–3), group II contained ZjCNGC4 and 5, and the remaining three ZjCNGC (13–15) members belonged to group IVB, while ZjCNGC12 belonged to group IVA. Moreover, some ZjCNGCs located on the same chromosome, such as ZjCNGC4 and ZjCNGC5, ZjCNGC6 and ZjCNGC7 showed little divergence and thus clustered into the same group, which might indicate that some segmental duplication of ZjCNGCs might have occurred during the genome evolutionary process of Chinese jujube.

### The phylogenetic tree and line charts for a lineage of gene groups for *ZjCNGC15*

**Fig. 2:**
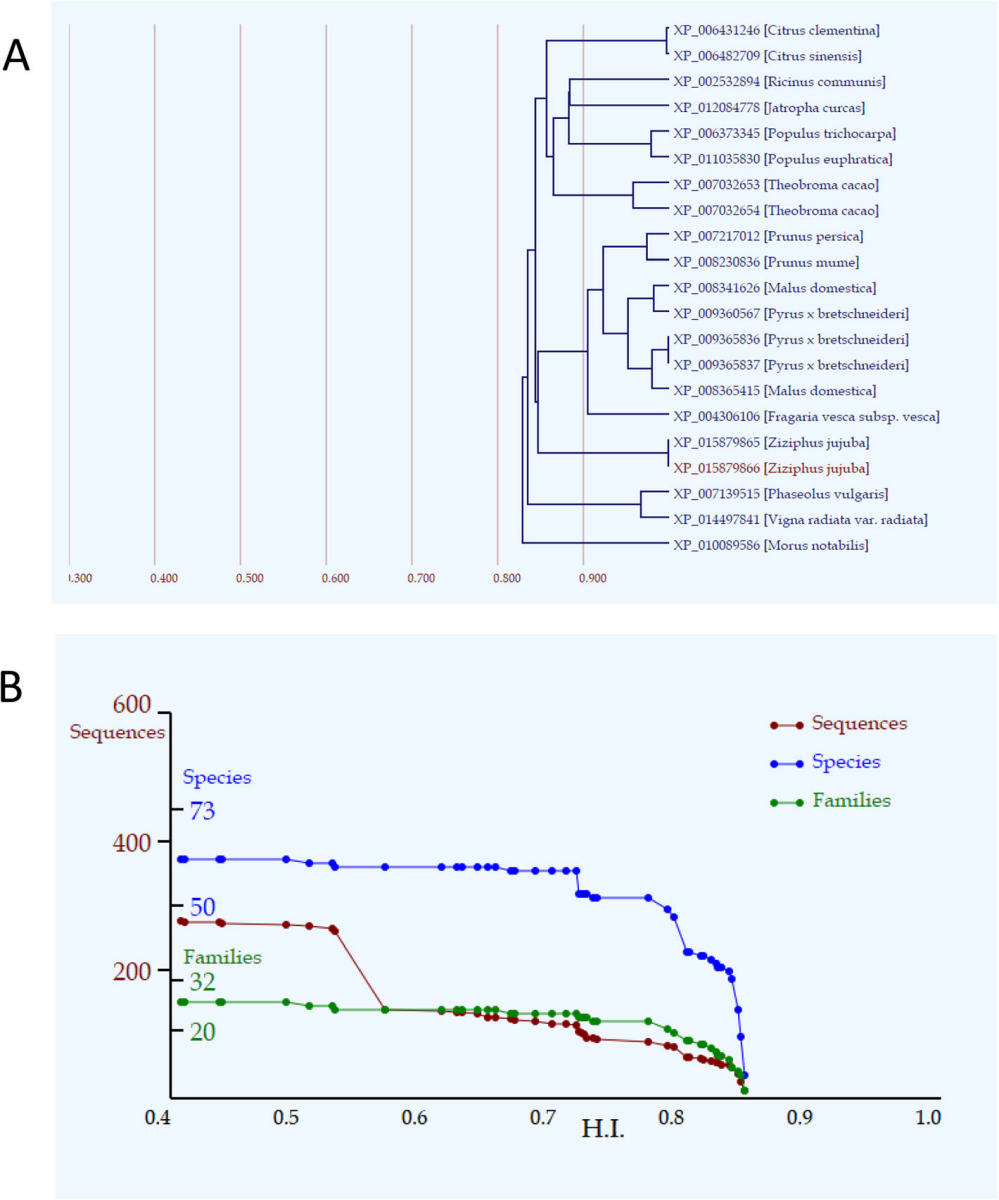
The phylogenetic tree and line charts for a lineage of gene groups for *ZjCNGC15*. **a** The tree contains a gene of interest and 20 genes with the highest *HI* values. The horizontal axis represents *HI*. **b** Red, blue, and green lines represent the numbers of genes (sequences), species, and families contained in individual gene groups, respectively. The horizontal axis represents *HI*

*ZjCNGC15*, belonging to group IVB, is homologous to *AtCNGC2*. *AtCNGC2* has been demonstrated to participate in multiple biological processes; thus, *ZjCNGC15* could be used to perform evolutionary analysis. As shown in Fig. 2a, 20 other genes with high homology indices (*HIs*) in different species were clustered together with *ZjCNGC15* (red colour). *ZjCNGC15* was more homologous to *Prunus* members and three paralogous events that presumably occurred in a group of three genes (*Pyrus bretschneideri* XP_009365836, XP_009365836, and *Malus domestica* XP_008365415), a group of two genes (*Citrus clementina* XP_006431246 and *Citrus sinensis* XP_006482709) and a group of two genes in *Ziziphus jujuba* (XP_015879866 and XP_015879865). In addition, the.

*HIs* in the tree were all above 0.8, showing that they have similar amino acid sequences and might have conserved functions. Based on the evolutionary time analysis with the lineage of gene groups for *ZjCNGC15*, the gene numbers (red line) were 260 at 0.528 of HI and 0.567 at 138 for a large decrease; however, the species number (blue line) showed no decrease at this time, indicating a paralogous event. At an *HI* of 0.792, the decrease occurred in both the number of sequences and species, indicating that this event could be presumably orthologous (Fig. 2b). Therefore, the paralogous and orthologous homology of *ZjCNGC15* occurred during the evolutionary process, and the other *ZjCNGCs* might have experienced a similar evolutionary process.

### Gene structure and motif composition analysis

**Fig. 3:**
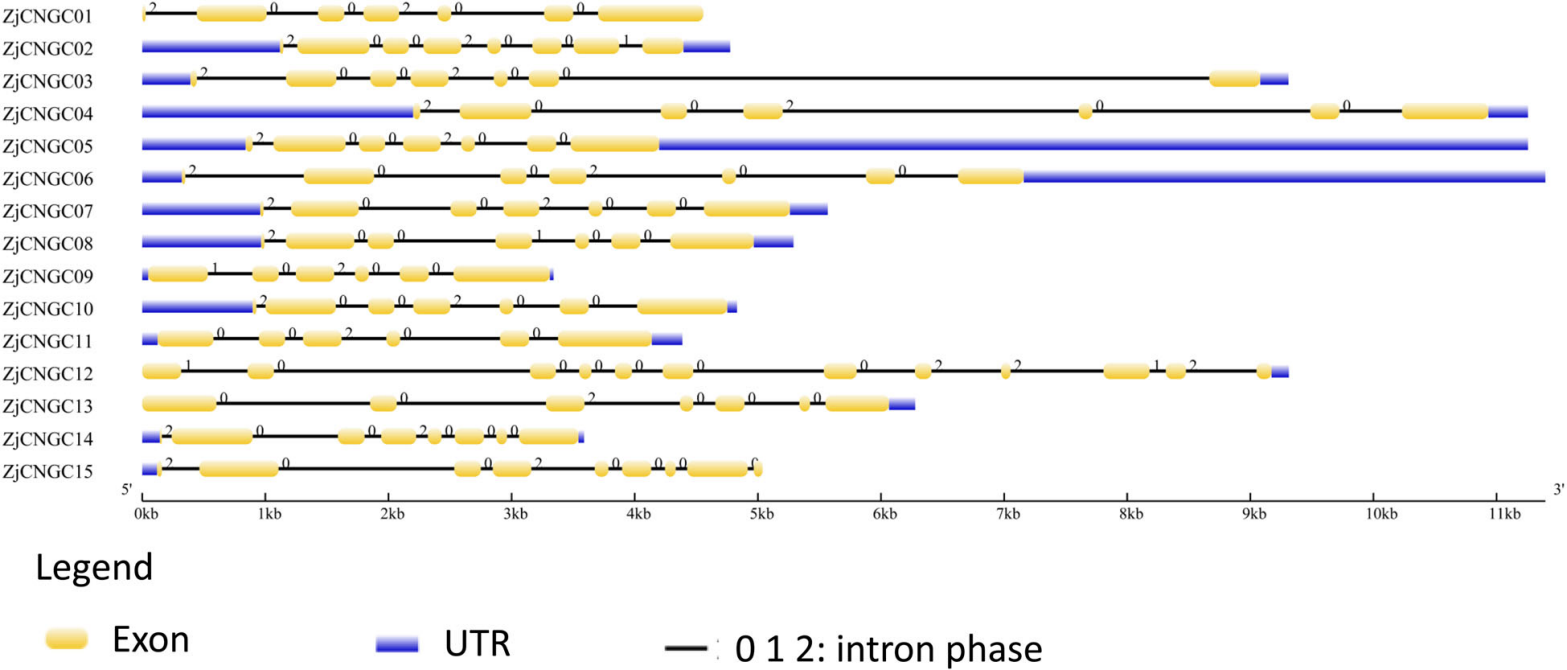
Schematic diagrams of the gene structures of *ZjCNGCs*. The yellow and blue boxes and the black lines indicate the exons, UTRs and introns, respectively; 0, 1 and 2 illustrate different intron phases

The divergence of gene structure, including exon-intron diversity, provides potential insights into the gene function during evolution. As shown in Fig. 3, the number of introns in all *ZjCNGCs* varied from 5 to 11, with an average number of 6. Interestingly, 9 of 15 *ZjCNGCs* indeed had 6 introns, and they were distributed in groups I, II, III and IVB (see additional file S5). However, *ZjCNGC12* had 11 introns that were totally different from the others. Moreover, the intron phase (0, 1 and 2) could indicate that the codons in the exon parts were interrupted by introns at different positions. Similar to *AtCNGCs*, most *ZjCNGCs* were in intron phase 0 and phase 2, while in *ZjCNGC2*, *8*, *9* and *12*, intron phase 1 could be observed. These results demonstrated the changes in terms of loss or gain of exons, intron phases and their shuffling, even in the same subgroup, which promoted functional divergences in the process of evolution.

**Fig. 4:**
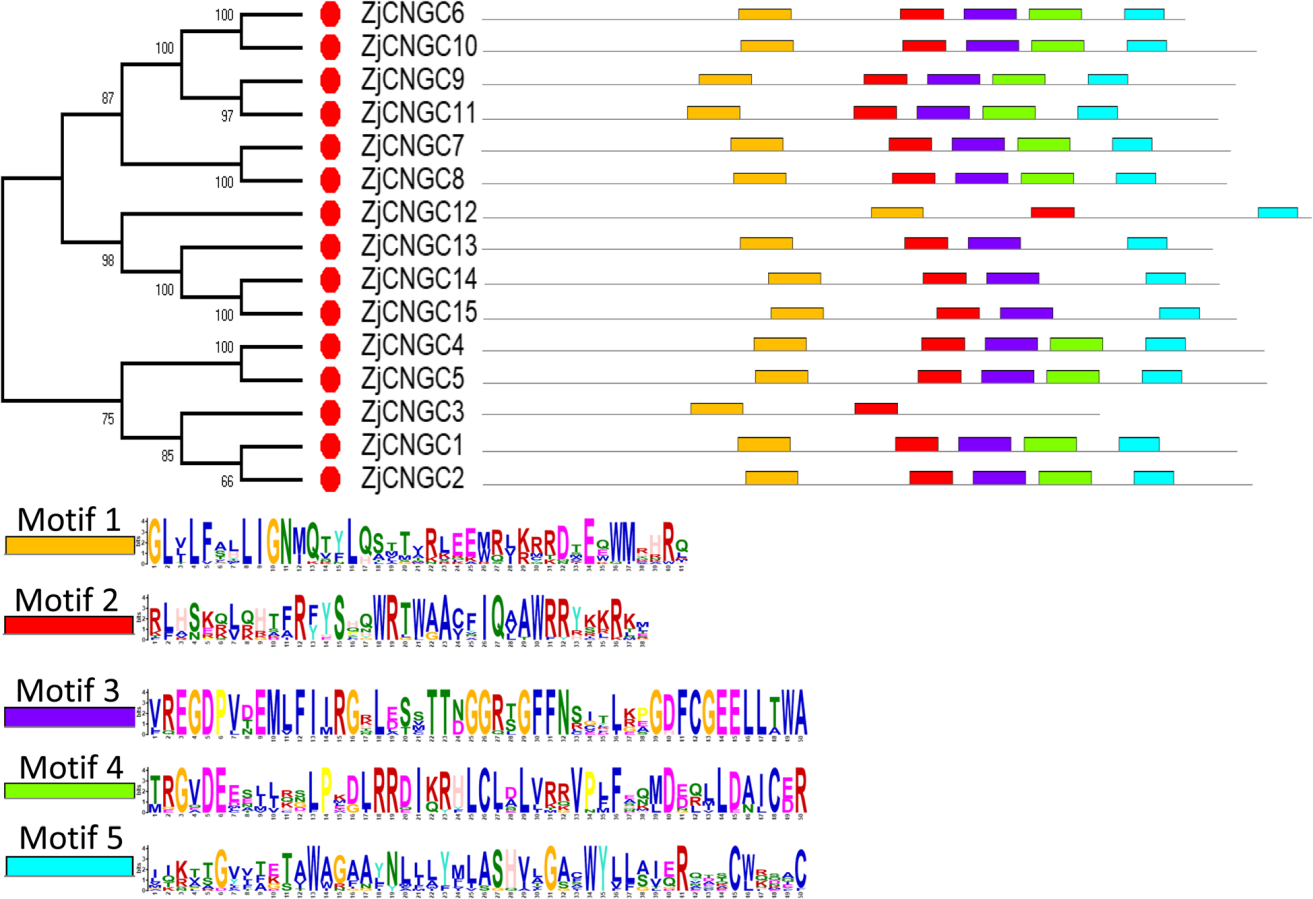
The motif compositions and motif logos of ZjCNGCs corresponding to the phylogenetic tree. The MEME database was used to perform motif identification according to the protein sequences

**Fig. 5:**
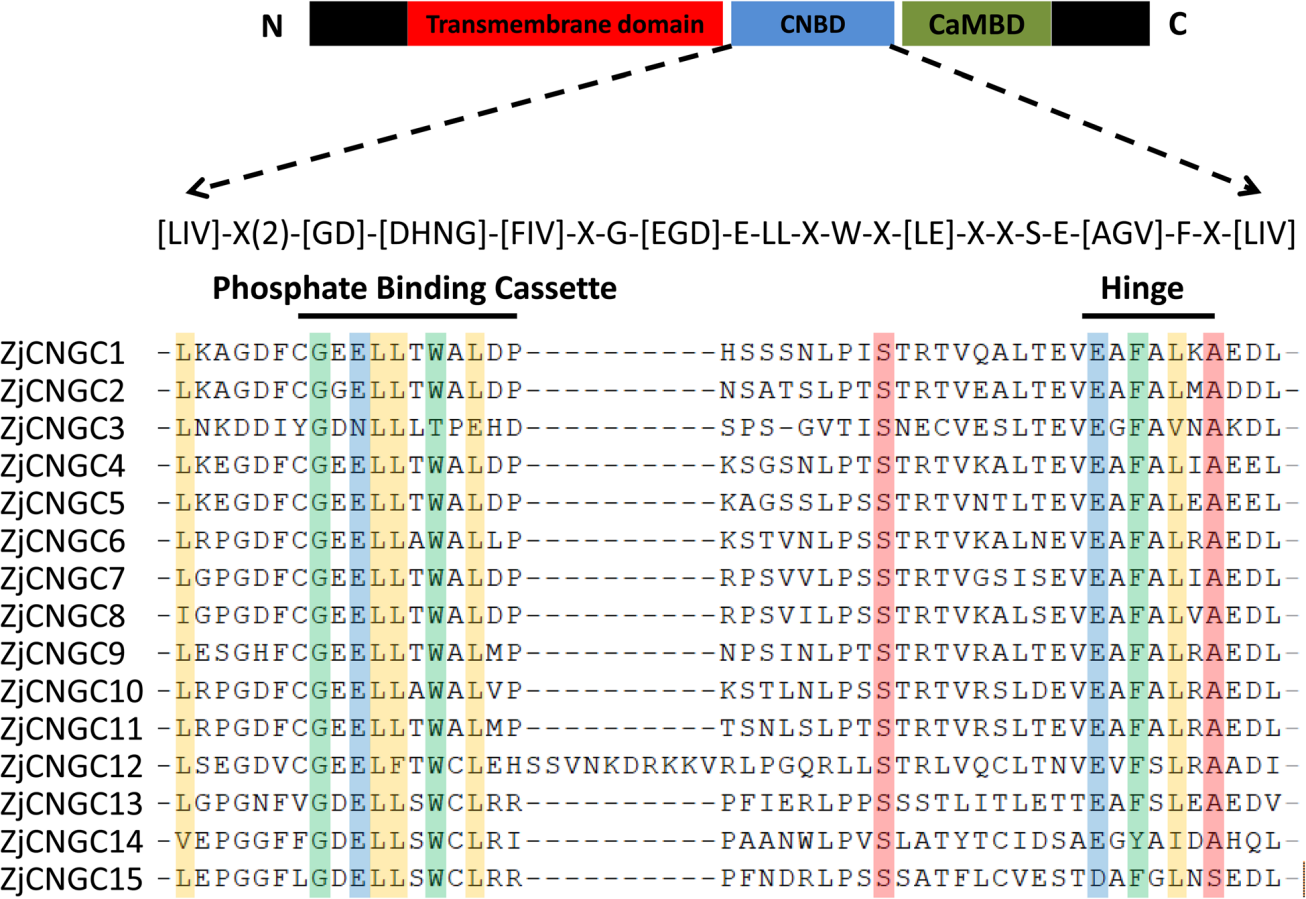
The putative PBC and hinge motifs within the CNBDs of all the ZjCNGCs. The diagram at the top represents three regions of plant CNGCs: the six transmembrane domains (TM), a CNBD containing a PBC and the hinge, and a CaMBD. The ZjCNGC-specific amino acid motif is shown below the cartoon. The square brackets in different colours indicate the highly conserved amino acids

The motif divergence of proteins could also provide insights into the evolutionary history of proteins [33]. Therefore, five motifs in ZjCNGCs were predicted with the MEME database. Except for ZjCNGC3 and 12, the other ZjCNGCs all contained the five motifs (Fig. 4). In addition, motif 2 represented the IQ domain (QWRTWAA [CV] FIQ [AL] AW [RH]RY), and motif 3 was the cyclic nucleotide-binding (CNB) domain, which was located in the middle. Motifs 1, 4 and 5 were the transmembrane domains located at the N- and C-terminal. These results indicated that the transmembrane domain and cNMP-binding domain were specific to plant CNGCs. Among them, the CNBD domain, which could bind cAMP/cGMP, was the main structure of plant CNGCs [34]. The CNBD domain contains two regions, including the PBC and hinge regions. As shown in Fig. 5, a > 90% conserved motif was found in 15 ZjCNGCs, [LIV]-X(2)-[GD]-[DHNG]-[FIV]-X-G-[EGD]-E-LL-X-W-X-[LE]-X-X-S-E-[AGV]-F-X-[LIV], which consisted of the PBC and hinge regions. In addition, the conserved glycine (G) and aliphatic leucine (L) residues in the PBC motif were 100% conserved inside the PBCs. This conserved motif was consistent with the analysis in wheat, maize and rice.

### Prediction of *Cis*-acting regulatory elements

To better elucidate the function of *ZjCNGCs*, which are regulated by their corresponding transcriptional factors in response to biotic and abiotic stresses, 1.0 kb noncoding sequences upstream of the *ZjCNGC* genomic sequences that belong to the promoter region were used to perform *cis*-regulatory element analysis with the PLACE database. The results showed that most of the promoter sequences of *ZjCNGCs* contained several *cis*-regulatory element regulatory sites for biotic and abiotic factors, such as BOXLCOREDCPAL, GT1GMSCAM4, SEBFCONSSTPR10A, MYCCONSENSUSAT, and MYCATERD1, suggesting that *ZjCNGCs* might be involved in regulating diverse stress responses (Table 2). However, the *ZjCNGC14* promoter did not have responsive *cis*-regulatory elements involved in biotic stresses and only contained 6 MYCCONSENSUSAT sequences in response to abiotic stress. Additionally, different WRKY transcription factor binding sites were found in all the promoter sequences of *ZjCNGCs*, including WBBOXPCWRKY1, BOXATNPR1, WBOXHVISO1, WBOXNTERF3, WRKY71OS and WBOXNTCHN48, which indicates that all *ZjCNGCs* were regulated by WRKY transcriptional factors. More importantly, Ca^2+^/calmodulin-binding *cis*-regulatory elements existed in the promoter regions of *ZjCNGC9* and *ZjCNGC11*, indicating that these two genes might be regulated by the Ca^2+^/calmodulin signal transduction pathway.

**Table 2:** Predicted *cis*-acting element in 1 kb upstream regions of the *ZjCNGC* genes

Regulator	Cis-acting element	Signal sequence	Code	Number of elements in 15 *ZjCNGCs*														
				1	2	3	4	5	6	7	8	9	10	11	12	13	14	15
Ca^2+^/Calmodulin binding	CGCGBOXAT	VCGCGB	S000501	0	0	0	0	0	0	0	0	3	0	2	0	0	0	0
Biotic	BOXLCOREDCPAL	ACCWWCC	S000492	0	2	1	0	1	0	0	0	1	1	0	1	1	0	0
	CACGTGMOTIF	CACGTG	S000042	0	0	0	0	0	0	0	0	0	0	2	0	0	0	0
	ELRECOREPCRP1	TTGACC	S000142	0	0	0	0	0	0	0	0	1	0	0	2	0	0	0
	GT1GMSCAM4	GAAAAA	S000453	7	3	3	5	2	5	4	4	6	6	2	7	7	0	2
	MYB1LEPR	GTTAGTT	S000443	1	1	0	0	0	0	0	0	0	0	0	0	0	0	0
	SEBFCONSSTPR10A	YTGTCWC	S000391	0	0	0	0	1	0	1	1	0	1	0	1	0	0	2
Abiotic	MYCATERD1	CATGTG	S000413	0	0	0	1	0	2	2	3	0	0	0	0	0	0	3
	MYCCONSENSUSAT	CANNTG	S000407	2	6	6	4	12	6	6	8	4	8	2	4	2	6	10
	MYB2AT	TAACTG	S000177	3	0	0	0	0	0	0	0	0	0	0	0	0	0	0
WRKY transcription factor	WBBOXPCWRKY1	TTTGACY	S000310	1	0	1	0	0	0	0	0	3	1	0	0	0	0	0
	WBOXATNPR1	TTGAC	S000390	3	0	2	3	2	1	1	4	5	2	0	5	1	0	0
	WBOXHVISO1	TGACT	S000442	2	0	3	0	3	2	3	1	3	3	1	2	1	0	1
	WBOXNTERF3	TGACY	S000457	3	2	5	0	4	4	3	2	5	3	2	5	1	2	2
	WRKY71OS	TGAC	S000447	3	2	5	4	6	7	7	8	7	4	2	9	3	3	6
	WBOXNTCHN48	CTGACY	S000508	0	0	0	0	1	1	2	0	1	0	0	2	0	0	0

### Posttranslational modification and phosphorylation of ZjCNGC proteins

After analyzing the *cis*-regulatory elements of *ZjCNGCs* in the promoter region, the posttranslational modification and phosphorylation of ZjCNGC proteins were analyzed by using ScanProsite. As shown in Table 3, protein kinase C, casein kinase II, cAMP/cGMP kinases, N-myristoylation sites and N-glycosylation sites were widely found in all ZjCNGCs. Among them, the casein kinase II sites were evenly distributed in all the ZjCNGC members, which have important functions in *Arabidopsis* in response to stresses [35]. Protein kinase C was most abundant in the group II and III subfamilies, which belong to a family of ten isoenzymes that play vital roles in cellular signal transduction [36]. Additionally, 1 to 4 cAMP/cGMP-binding motifs were found in all ZjCNGCs, indicating that all ZjCNGCs were targets of cAMP/cGMP involved in signal transduction. However, the leucine zipper patterns and amidation sites were not conserved and randomly distributed in some ZjCNGCs.

**Table 3:** Distribution of phosphorylation and other posttranslational modification sites in ZjCNGC-encoded protein sequences

Gene ID	cAMP/cGMP	PKC	N-MYR	ASN	TYR	CK2	AMD	LEU
ZjCNGC1	3	14	7	6	1	8		
ZjCNGC2	1	14	7	2	2	5		
ZjCNGC3	2	8	10	3	1	9		
ZjCNGC4	4	15	15	7	1	7	1	1
ZjCNGC5	4	15	6	2	1	8		1
ZjCNGC6	1	10	5	2	1	5		
ZjCNGC7	1	15	9	2	1	6		1
ZjCNGC8	2	13	6	5	1	8		
ZjCNGC9	3	11	8	5	1	11		
ZjCNGC10	3	8	8	3	1	7		
ZjCNGC11	3	13	6	5	2	9		
ZjCNGC12	2	11	5	7	2	9	1	
ZjCNGC13	1	6	10	4		10	1	2
ZjCNGC14	1	8	5	6	1	6		
ZjCNGC15	1	7	8	6	1	9		

Note: *cAMP/cGMP* cAMP/cGMP-binding motif profile, *CK2* casein kinase II phosphorylation site, *AMD* amidation site, *PKC* protein kinase C phosphorylation site, *ASN* N-glycosylation site, *TYR* tyrosine kinase phosphorylation site, *MYR* N-myristoylation site, *LEU* leucine zipper pattern

### Expression patterns of *ZjCNGCs* in response to biotic and abiotic stresses

**Fig. 6:**
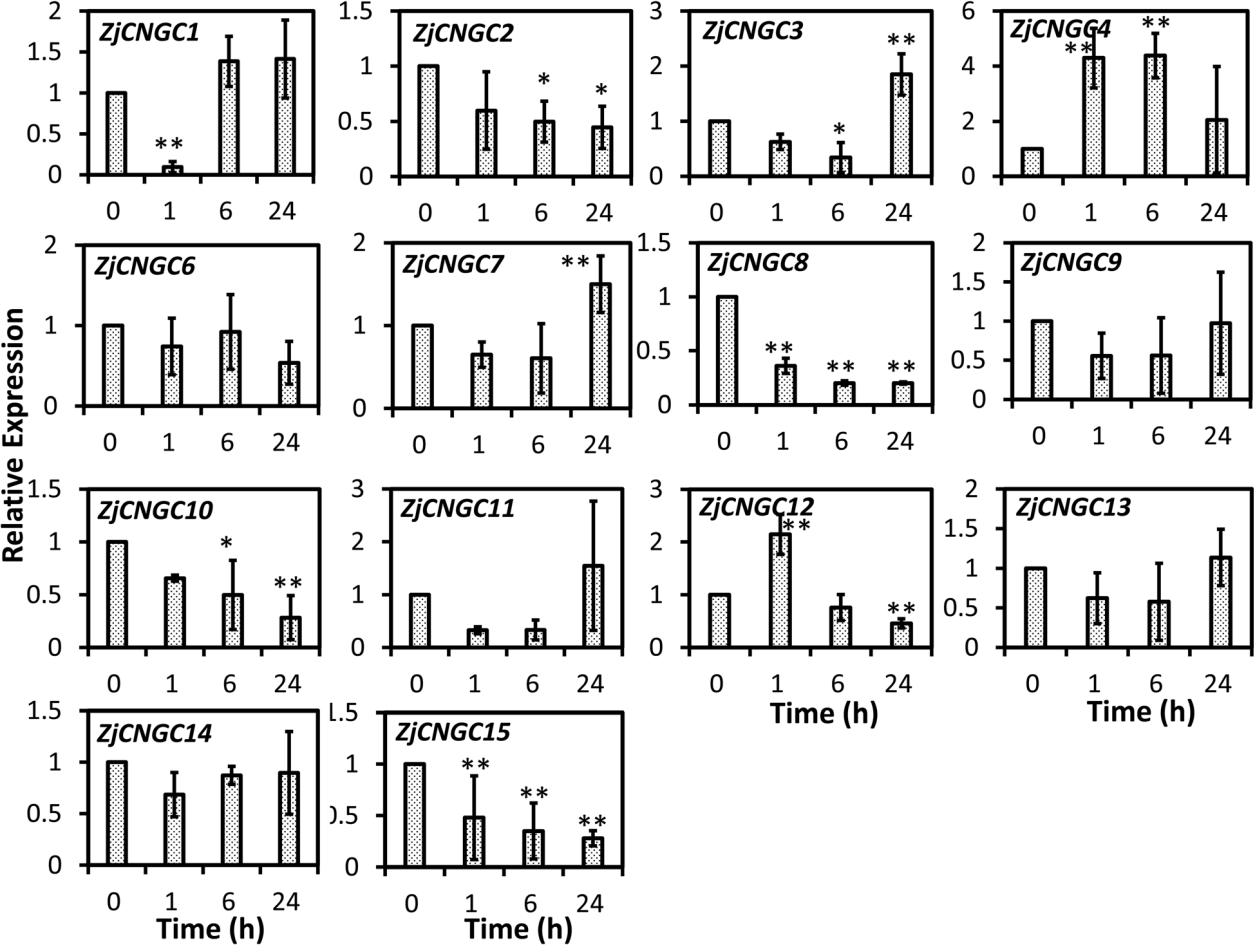
Relative expression profiles of *ZjCNGCs* in response to cold stress. The relative expression level of the representative members of *ZjCNGCs* in three independent replications, and the error bar represents the standard deviation (SD). Asterisks indicate that the corresponding gene was significantly up- or downregulated at different time points compared with the gene expression at the 0 h time point (*P < 0.05, **P < 0.01)

To determine whether *ZjCNGCs* were associated with abiotic stresses, the expression profiles of *ZjCNGCs* in response to cold, salt and alkaline stresses were investigated. Unfortunately, the expression level of *ZjCNGC5* was not detected in any of the treatments. Therefore, the remaining fourteen *ZjCNGCs* were used for qPCR analysis. As shown in Fig. 6, some *ZjCNGCs*, such as *ZjCNGC*2, 8, 10 and 15, were downregulated in response to cold stress within 24 h of treatment. The expression levels of *ZjCNGC4* and *12* increased approximately 4- and 2-fold after 1 h of cold treatment, respectively. The expression level of the others showed no significant changes or increased/decreased at one time point.

**Fig. 7:**
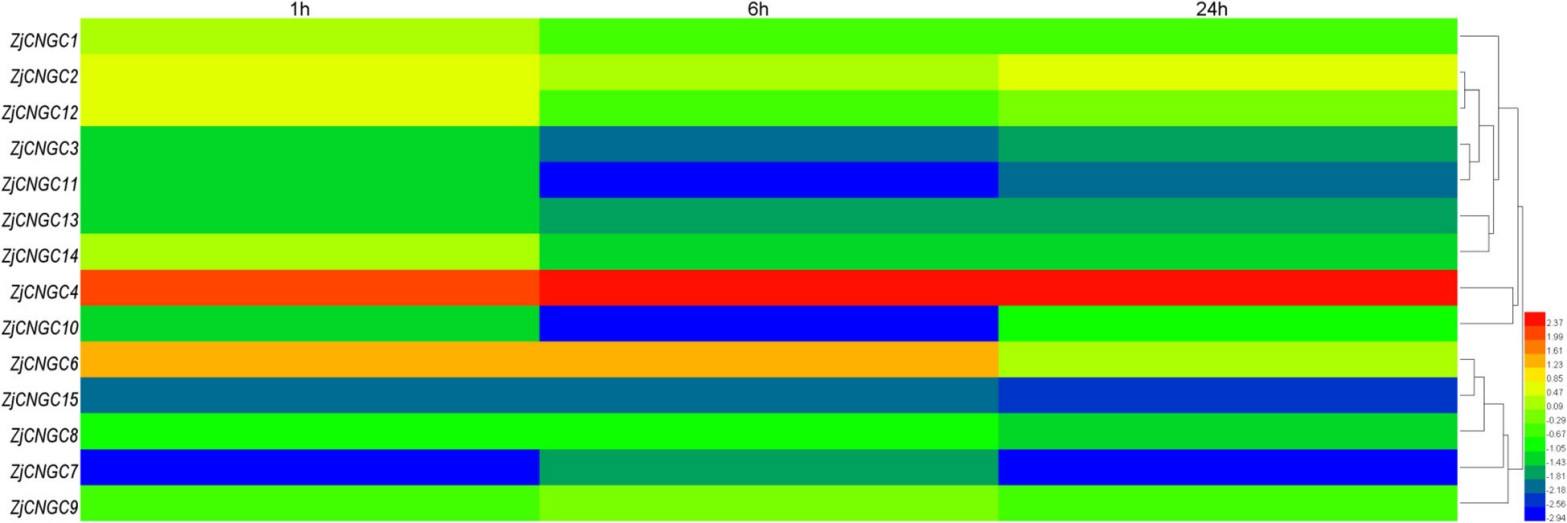
Heatmap analysis of the relative expression profiles of *ZjCNGCs* in response to salt stress. Different colours indicate that the corresponding gene was significantly up- or downregulated at different time points compared with the gene expression at the 0 h time point

**Fig. 8:**
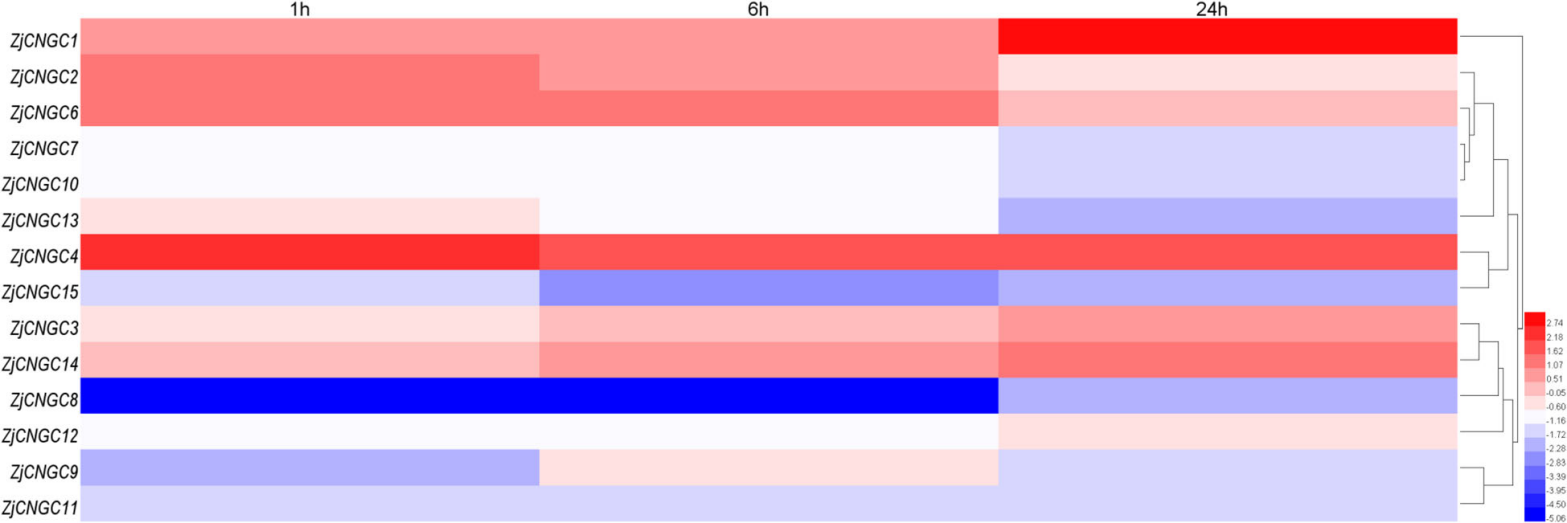
Heatmap analysis of the relative expression profiles of *ZjCNGCs* in response to alkaline stress. Different colours indicate that the corresponding gene was significantly up- or downregulated at different time points compared with the gene expression at the 0 h time point

In response to salt stress, most of the *ZjCNGCs* were downregulated, especially the genes from group III and group IVB (Fig. 7). The same changes in *ZjCNGCs* in response to alkaline stress were observed (Fig. 8). However, *ZjCNGC2*, *4* and *6* were highly induced at the early time point (1 h) under these two treatments. In summary, some *ZjCNGC* genes were significantly induced or repressed by the above treatments.

**Fig. 9:**
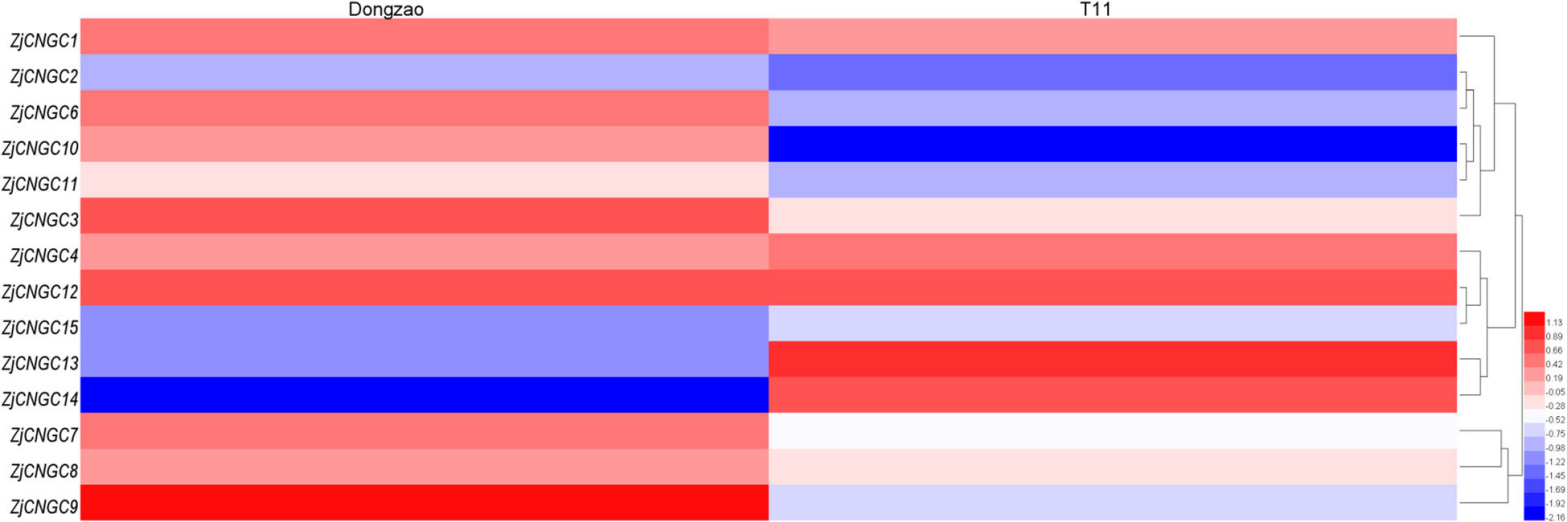
Heatmap analysis of the relative expression profiles of *ZjCNGCs* in response to phytoplasma infection. Different colours indicate that the corresponding genes were significantly up- or downregulated between these two cultivars

The response of *ZjCNGCs* to phytoplasma infection was also investigated. As shown in Fig. 9, most of the *ZjCNGCs* showed no significant difference between the phytoplasma-resistant cultivar “T11” and the susceptible cultivar “Dongzao”. In addition, the expression level of *ZjCNGC10* decreased in the resistant cultivar but not in the susceptible cultivar, while *ZjCNGC13* and *14* were highly induced in the resistant cultivar and downregulated in the susceptible cultivars, indicating that these four genes could have important functions in the process of phytoplasma infection.

### *ZjCNGC2* mediated ZjMAPK cascades involved in cold stress

**Fig. 10:**
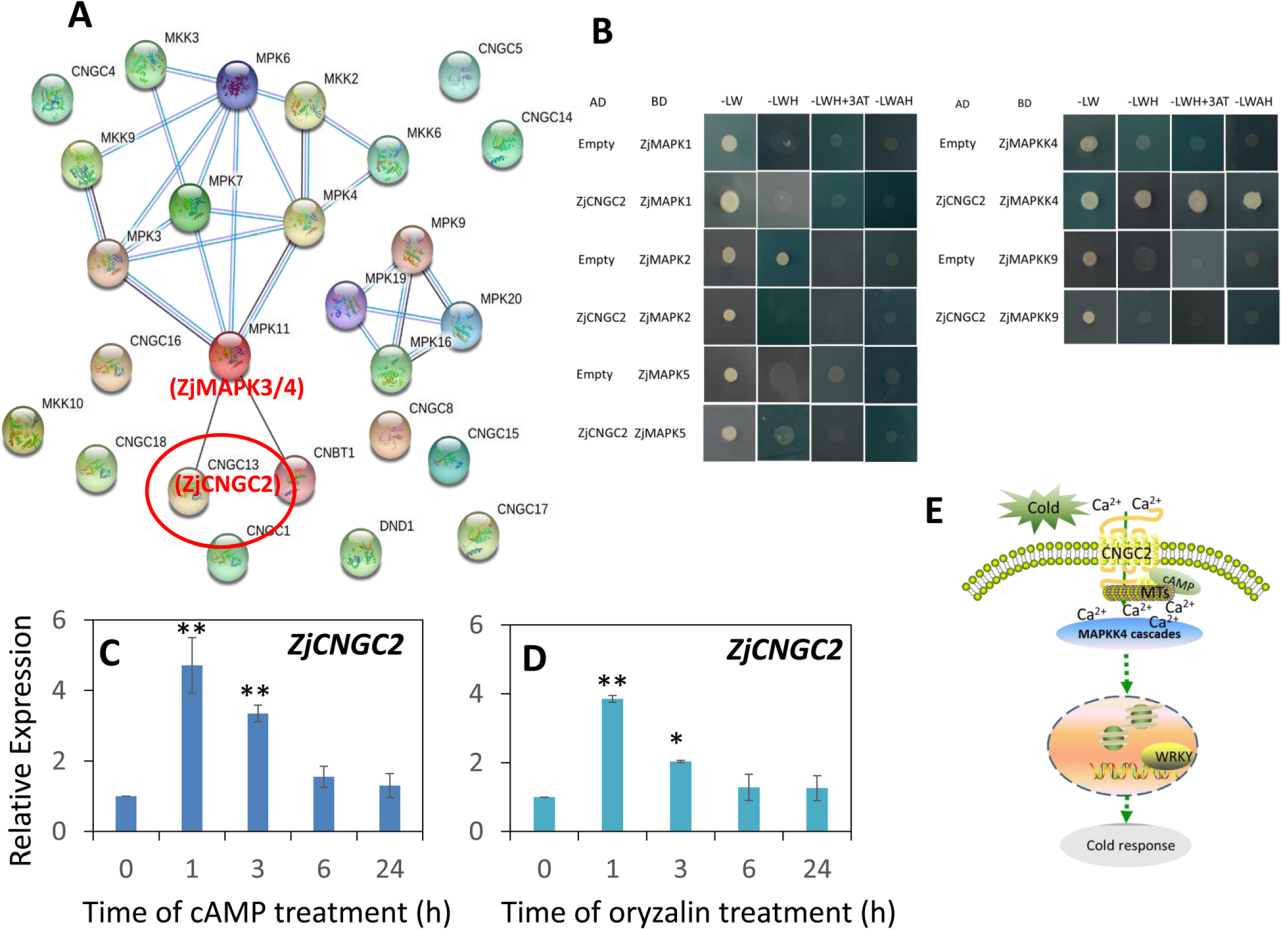
*ZjCNGC2*-mediated signalling cascades in response to cold stress. **a** Protein-protein interaction analysis among 15 ZjCNGCs, 10 ZjMAPKs and 5 ZjMAPKKs by the STRING database. ZjCNGC2 is orthologous to AtCNGC13, and ZjMAPK3/4 are orthologous to AtMAPK11. **b** Yeast two-hybrid screening of ZjCNGC2 and ZjMAPKK4. Relative expression profiles of *ZjCNGC2* in response to 50 μM cAMP (**c**) and 10 μM oryzalin (**d**) treatments at normal temperature. **e** Schematic and supposed model of the cAMP- and microtubules (MTs) regulated ZjCNGC2-ZjMAPKK4 signalling pathway involved in cold stress

Some *ZjCNGCs* were downregulated or upregulated in response to cold stress, indicating that they might play important roles in cold stress. Thus, to test whether the ZjCNGCs could regulate the ZjMAPK cascades during cold-induced signal transduction, the amino acids of 15 ZjCNGCs, 10 ZjMAPKs and 5 ZjMAPKKs were submitted to the STRING database for protein-protein interaction prediction. As shown in Fig. 10a, AtCNGC13, which is orthologous to ZjCNGC2, might interact with AtMAPK11, which is orthologous to ZjMAPK3/4, indicating that a protein-protein interaction existed between ZjCNGCs and ZjMAPK cascades. Then, the prediction was tested by yeast two-hybrid analysis, and ZjCNGC2 interacted with ZjMAPKK4 but not with ZjMAPKs (Fig. 10b). Moreover, to test whether cAMP or changes in microtubules (MTs) could regulate the activity of ZjCNGC2, the expression level of *ZjCNGC2* was analysed in the presence of cAMP or the MTs-disrupting drug oryzalin, respectively. The results showed that *ZjCNGC2* rapidly (in 1 h) responded to these two drug treatments (Fig. 10c and d). All these results might indicate that cAMP and MTs play important roles in *ZjCNGC2*-mediated *ZjMAPKK4* signal transduction under cold stress.

## Discussion

Plant cyclic nucleotide-gated channels (CNGCs) have important functions in Ca^2+^ signal transduction, including pollen tube growth, thermosensing, pathogen resistance [1, 9], and have been widely identified in plant systems. However, the genome-wide identification and functional analysis of *CNGCs* in Chinese jujube have not been reported. In the current study, 15 *ZjCNGCs* were identified. Among them, *ZjCNGC3* might be controversial because it only contains the ITP domain based on SMART analysis. However, based on InterProScan analysis, *ZjCNGC3* contains a cyclic nucleotide-binding-like signature. In addition, based on domain comparative analysis, the ZjCNGC3 protein contains a PBC and hinge region in the CNBD domain with only several amino acid discrepancies. These results could support that *ZjCNGC3* should be bona fide member of *ZjCNGCs*. The same observation could be found in the *OsCNGC* family in which the amino acid sequence of LOC_Os06g33610 was shorter than those of the other *OsCNGCs*; however, this protein contained the CNGC characteristic phosphate-binding cassettes and hinge regions and was finally designated as bona fide *OsCNGC3* [13]. For accuracy purposes, the *ZjCNGC3* gene should be cloned and sequenced for further analysis. Moreover, the phylogenetic and gene structure analyses showed that *ZjCNGCs* have similar structures and gathered with *Arabidopsis* and pear counterparts, and they all could be divided into five groups with significant bootstrap values. Together with the evolutionary analysis of *ZjCNGC15*, paralogous and orthologous homology duplication processes might have taken place during the evolutionary process of *ZjCNGCs*, implying that duplication events play a principal role in gene evolution.

Protein posttranslational modification could modulate protein activity and affect protein functions through different mechanisms in response to stresses, including protein-protein interactions, protein localization, degradation, cleavage or allosterically regulating enzyme activity [37]. Among them, cAMP/cGMP could target *ZjCNGCs*; of all reported plant species, cAMP had the highest concentration in Chinese jujube, and *CNGCs* have been confirmed to be the target of cAMP [6]. Therefore, the cAMP-regulated *ZjCNGC* signalling pathway might have important functions in response to various stresses. This conclusion has been demonstrated by our results that *ZjCNGC2* was rapidly induced with cAMP treatment, indicating that CNGC2 might be the target of cAMP in the jujube system. In addition, cyclic nucleotides have been suggested to be involved in plant responses to biotic and abiotic stresses, while based on *cis*-regulatory element analysis, nearly all *ZjCNGCs* contain several *cis*-regulatory element sites for biotic and abiotic factors. More interestingly, *ZjCNGC9* and *ZjCNGC11* have Ca^2+^/calmodulin-binding *cis*-regulatory elements, which could be interesting for further research on Ca^2+^/calmodulin- and *ZjCNGC*-mediated signalling pathways in Chinese jujube.

Based on the localization prediction analysis, all ZjCNGCs are located on the cell membrane, including the plasma membrane, chloroplast thylakoid membrane or mitochondrial inner membrane, as reported by Nawaz et al. (2014) [13] in the analysis of rice. In addition, regarding the CNGC proteins in *Arabidopsis*, 11 out of the 20 CNGC proteins were localized in the plasma membrane, while the other 9 were located in the chloroplast thylakoid membrane [13]. These results suggested that CNGC proteins were mainly localized in the cell membrane, and CNGC proteins might have important functions in the regulation of Ca^2+^ influx in response to biotic and abiotic stresses. Furthermore, calcium influx into the cytoplasm in response to cold stress has been demonstrated [38–40], and CNGCs could be one of the potential Ca^2+^-permeable channels [1, 2]. In the current study, *ZjCNGC4* was upregulated at an early time point, and *ZjCNGC2* was downregulated after 24 h of cold treatment, demonstrating that these two genes play different roles in the response to cold stress. Moreover, *ZjCNGC4* was also highly induced with salt and alkaline treatments, indicating that it might have an important function in calcium influx into the cytoplasm in response to abiotic stresses. *ZjCNGC4* was more homologous to *AtCNGC6*, while *AtCNGC6* could induce Ca^2+^ influx in response to heat shock and facilitate the expression of heat shock protein (HSP) genes to achieve thermotolerance [41]. In addition, MTs have been demonstrated to play an important role in the response to cold stress, and modulation of MTs with their corresponding drugs could improve plant cold resistance [40]. Our results showed that *ZjCNGC2* could be regulated by cAMP and oryzalin treatments, indicating that cAMP and MTs could regulate ZjCNGC2 activity for early signal transduction. Furthermore, ZjCNGC2 could interact with ZjMAPKK4, and ZjMAPKKs have been demonstrated to interact with ZjWRKYs [42]. Finally, we arrived at a supposed model of cAMP- and MTs - regulated ZjCNGC2 signalling pathways involved in cold stress (Fig. 10e). Under cold stress, Ca^2+^ influx into the cytosol was triggered by cold stress through the CNGC channel. However, the expression of *ZjCNGC2* was repressed by cold stress, indicating that it might play a negative role in calcium influx. The activity of ZjCNGC2 was regulated by cAMP and MTs depolymerization. Then, ZjCNGC2 could interact with ZjMAPKK4, which might further interact with ZjWRKYs to induce the cold response.

In comparison with their function in abiotic stress, some more valuable clues about the functional role of *ZjCNGCs* in plant immunity were obtained. Many studies have demonstrated that CNGCs play an important role in biotic stress. For example, *AtCNGC2* could control the Ca^2+^ concentration to regulate the hypersensitive response (HR) to avirulent pathogens [43]. Moreover, the *AtCNGC2* orthologue (*Arabidopsis Defense No Death* 1) in potato and tomato negatively regulated the resistance to late blight and powdery mildew and resulted in dwarfing and necrosis in tomato but not in potato [28]. In addition to the crucial role of *CNGC2* in plant immunity, dnd1 (null mutant of *CNGC2*) could lead to heat stress with the accumulation of heat shock proteins [44, 45]. According to our results, *ZjCNGC15* was downregulated in the phytoplasma-susceptible cultivar “Dongzao”, but there was no significant difference compared to *ZjCNGC15* in the resistant cultivar “T11”. However, in group IVB, *ZjCNGC13* and *14* were highly induced in the resistant cultivar but downregulated in the susceptible cultivar, demonstrating that these two genes might be the main channels to induce calcium influx into the cytoplasm in the resistant cultivar to obtain resistance to phytoplasma infection. Again, this hypothesis should be confirmed at the protein expression level for *ZjCNGC13* and *14*. Overall, these results imply that these genes play crucial roles in the response to biotic and abiotic stresses in Chinese jujube.

## Conclusions

Altogether, systematic informatics analyses of 15 *CNGCs* in Chinese jujube were firstly carried out, including phylogenetic, conserved motif and intron/exon analyses. Moreover, the expression profiles of *ZjCNGCs* were detected by qPCR in response to cold, salt and alkaline stresses and phytoplasma infection. *ZjCNGC2* was significantly downregulated in response to cold stress, and *ZjCNGC4* was highly induced in response to cold, salt and alkaline stresses. *ZjCNGC13* and *14* were highly induced in phytoplasma-resistant cultivars and downregulated in susceptible cultivars. Furthermore, *ZjCNGC2* could be regulated by cAMP and MTs disruptions and could interact with ZjMAPKK4, which suggested that cAMP and MTs might play important roles in *ZjCNGC2*-mediated *ZjMAPKK4* signalling transduction involved in cold stress.

## Methods

### Identification of *CNGC* genes in Chinese jujube

The reference genome and genome annotation of Chinese jujube (*Ziziphus jujuba* Mill.) was used for the identification of the candidate CNGC genes (https://www.ncbi.nlm.nih.gov/genome/15586). First, a total of 20 *Arabidopsis* and 21 pear protein sequences were obtained from the TAIR database (https://www.arabidopsis.org/) and pear genome database (http://www.peargenome.njau.edu.cn), respectively. Then, to find the potential *ZjCNGC* genes, BLASTP searches were performed in NCBI (http://www.ncbi.nlm.nih.gov/) against the whole jujube genome database with the 20 *Arabidopsis* and 21 pear CNGC proteins as queries. Moreover, all the retrieved nonredundant sequences were collected and subjected to domain analysis by using the InterProScan (http://www.ebi.ac.uk/Tools/pfa/iprscan5/) and SMART (http://smart.embl-heidelberg.de/) programs. In addition, all potential *ZjCNGC* genes were confirmed by HMMER analysis, which contains both the CNBD [cNMP_binding family (PF00027)] and the ion transport protein domain [ITP, Ion_trans family (PF00520)] [13, 14]. The protein sequences with six membrane-spanning regions, a pore region, a CNBD, a CaMBD or an IQD were recognized as ZjCNGC proteins [15].

### Protein characterization and amino acid properties

Information on protein and CDS lengths, chromosomal locations and exon numbers was obtained from the NCBI database. The molecular weight (kDa), instability indices and isoelectric points (pI) of individual amino acids were obtained by ProtParam analysis (http://web.expasy.org/protparam/) [46]. The post-translational modification (PTM) sites were predicted with the ScanProsite web server (http://prosite.expasy.org/scan prosite/) [47]. In addition, the subcellular localization of the ZjCNGC proteins was identified by the Plant-mPLoc database (http://www.csbio.sjtu.edu.cn/bioinf/plant-multi/) [48].

### Multiple sequence alignment and phylogenetic analysis

The protein sequences of 15 ZjCNGCs, 20 AtCNGCs and 21 PbrCNGCs were aligned by ClusterX software [49] with default parameters. The conserved CNGC-specific domains were manually checked. Then, the alignment of the above protein sequences was obtained, and phylogenetic analysis was performed by using MEGA 6.0 with the neighbour-joining (NJ) method. The number of bootstrap replications was 1000 with a Poisson distribution [50]. In addition, the phylogenetic tree and line chart for a lineage of gene groups for *ZjCNGC15* were analyzed in the Gcorn plant database (http://www.plant.osakafu-u.ac.jp/~kagiana/gcorn/p/) [51].

### Gene structure, conserved motif domain and *cis-*acting regulatory element analysis

The intron/exon structure information of *ZjCNGCs* was obtained from the NCBI database, and the detailed exon/intron structures of the *ZjCNGCs* were constructed with Gene Structure Display Server (GSDS 2.0; http://gsds.cbi.pku.edu.cn/) [52]. To confirm the structural divergence of the *ZjCNGC* protein sequences, the conserved motifs in the ZjCNGC protein sequences were identified with the MEME (Multiple Expectation Maximization for Motif Elicitation) web server (http://meme-suite.org/), with the following parameters: the optimal motif width was set between 6 and 200, and the maximum number of different motifs was set at 5 [53]. The 1000 bp DNA sequences upstream of *ZjCNGCs* were used to perform *cis*-acting regulatory element analysis by the “Signal Scan Search” program in the PLACE database (https://sogo.dna.affrc.go.jp/cgi-bin/sogo.cgi?lang=en&pj=640&action=page&page=newplace) [54].

### Plant materials and treatments

To investigate the function of *ZjCNGCs* in response to abiotic stresses, cold, salt and alkaline treatments were conducted on the callus tissues of *Ziziphus jujuba* Mill. ‘Guanyangchangzao’. For cold treatment, the calluses were transferred to 4 °C and then collected within 0, 1, 6 and 24 h. Calluses incubated at 25 °C were collected as a negative control. For the salinity and alkaline treatments, the callus tissues were subjected to 150 mM NaCl and NaHCO_3_-NaOH solution (pH 9.5) [55] for 0, 1, 6 and 24 h, respectively. The samples with sterile water treatment rather than NaCl and NaHCO_3_-NaOH solution were used as a negative control. In addition, to gain insight into whether *ZjCNGC2* could be regulated by cyclic adenosine monophosphate or microtubules, the same callus tissues were treated with 50 μM cAMP and 10 μM oryzalin at normal temperature (25 °C) for 0, 1, 3, 6 and 24 h. The samples with sterile water and the same amount of DMSO treatments were collected as a negative control.

For biotic stress, the leaves from the phytoplasma-susceptible cultivar *Ziziphus jujuba* Mill. ‘Dongzao’ and the resistant cultivar *Ziziphus jujuba* Mill. ‘T11’ that were grafted on rootstocks with JWB and healthy rootstocks [56] were collected in June 2018. Three independent replicates were performed for each treatment, and all the treated tissue samples were immediately frozen in liquid nitrogen and stored at − 80 °C for RNA extraction and qPCR analysis.

### RNA extraction and qPCR analysis

Total RNA extraction from the calluses or leaves and subsequent cDNA synthesis were performed as described by Liu et al. (2017) [57]. qPCR was carried out on a Bio-Rad iQ™ 5 instrument using TransStart Top Green qPCR SuperMix AQ131 (TransGen Biotech, China). The 20 μL reaction system contained 10 μL of 2 × SYBR Premix ExTaq™, 0.4 μL of each of 10 μM primers, 1 μL of diluted cDNA and 8.2 μL of ddH_2_O. The reactions were performed as follows: preincubation for 3 min at 94 °C, followed by 40 cycles of 5 s at 94 °C, 15 s at 55~63 °C and 15 s at 72 °C. Relative expression levels of *ZjCNGCs* were calculated with the 2^-ΔΔCt^ method [58] using *ZjActin* as an endogenous control for normalization [59]. The primer sequences of *ZjCNGCs* used for qPCR are shown in additional file S6.

### Heatmap construction

The expression profiles of all *ZjCNGCs* in response to salt, alkaline and phytoplasma stresses are illustrated by a colour gradient heatmap. The heatmap was constructed by heatmap software Heml 1.0 with Log2-based expression fold-changes.

### Protein-protein interaction prediction and yeast two-hybrid analysis

The amino acid sequences of 15 ZjCNGCs, 10 ZjMAPKs and 5 ZjMAPKKs were submitted to the STRING database (https://string-db.org/) for protein-protein interaction analysis. The orthologues of *Arabidopsis thaliana* were selected as references.

For the yeast two-hybrid analysis, ZjCNGC2 was fused to the Gal4 activation domain (AD), and ZjMAPKs/ZjMAPKKs were fused to the Gal4 DNA-binding domain (BD). Then, the constructs were cloned into the pGADT7 and pGBKT7 vectors, respectively, and the constructive vectors were co-transformed into the AH109 strain. Finally, the yeast clones were selected on synthetically defined medium lacking tryptophan and leucine (−LW) and medium lacking leucine, tryptophan, histidine (−LWH), −LWH medium supplemented with 7 mM 3-amino-1,2,4-triazole (3-AT; Sigma Aldrich) (−LWH + 3AT) and medium lacking leucine, tryptophan, histidine, adenine (−LWAH) as described by Xue et al. (2019) [42].

## Supplementary information

**Additional file S1. **The protein sequences of the CNGC genes from *Ziziphus jujuba* Mill.

**Additional file S2. **The CDS sequences of *ZjCNGCs*.

**Additional file S3.** Domain analysis of ZjCNGCs by using the InterProScan program.

**Additional file S4.** Domain analysis of ZjCNGCs by using the SMART program.

**Additional file S5. **The number of introns and exons of *ZjCNGCs*.

**Additional file S6. **The primer sequences for amplification of the *ZjCNGC* genes for qPCR.

## Data Availability

All data and materials are presented in the main paper and additional file.

## Abbreviations

AMD: Aidation site
ASN: N-glycosylation site
Ca^2+^: Calcium
CaM: Calmodulin
CaMBD: Calmodulin binding domain
cAMP/cGMP: Cyclic adenosine monophosphate/Cyclic guanosine monophosphate
CK2: Csein kinase II phosphorylation site
CNBD: Cyclic nucleotide-binding domain
CNGC: Cyclic nucleotide gated channel
HIs: Hmology indices
HSP: Hat shock protein
LEU: Lucine zipper pattern
MTs: Microtubules
MYR: N-myristoylation site
PBC: Posphate-binding cassette
PKC: Potein kinase C phosphorylation site
TM: Transmembrane
TYR: Trosine kinase phosphorylation site
