# Supplementary material for: Genome-wide identification of CNGC genes in Chinese jujube (Ziziphus jujuba Mill.) and ZjCNGC2 mediated signalling cascades in response to cold stress
Source: BMC Genomics. 2020 Mar 2;21:191. doi: 10.1186/s12864-020-6601-5 (PMC7053155; doi:10.1186/s12864-020-6601-5)
Supplement: Supplementary file 1 — Additional file S1. The protein sequences of the CNGC genes from Ziziphus jujuba Mill. [file 12864_2020_6601_MOESM1_ESM.pdf]

>ZjCNGC1
MNFQQDKFVRFQDWNSEKNTGVLYSPNNDTHPGRIRTTIDTVSDKFQRGLESSSERIKRIKQSLKSCSFG
SVMAKSWVSQKKVLDPQGPFLQKWNKIFVLSCVIAVSLDPLFFYVPVIDDKRKCLDLDGKMEITASVLRW
FTDLFYITHIIFQFRTGFIAPSSRVFGRGVLVEDPWAIAKRYLSTYFLIDILAVLPLPQVVILIIIPKLG
GSTSLNTKNLLKFVVLFQYIPRVIRIYPLYTEVTRTSGILTETAWAGAAFNLLLYMLASHVLGAFWYLFS
IERQTKCWQDACEMNNTLCNTASLYCENSSNIQLRTFLNDSCPIQEDSPKFNFGISLDALQSHVVDSTTD
FPQKLFYCFWWGLRNLSSLGQNLETSTYVWEICFAVFISIAGLVLFSFLIGNMQTYLQSTTTRLEEMRVK
RRDAEQWMAHRLLPENLRERIRRYEQYKWQETRGVDEENLICNLPKDLRRDIKRHLCLALLMRVPMFEKL
DEQLLDALCDRLKPVLYTEESYIVREGDPVDEMLFIMRGRLLTMTTNGGRTGFFNSEYLKAGDFCGEELL
TWALDPHSSSNLPISTRTVQALTEVEAFALKAEDLKFVASQFRRLHSKQLRHTFRFYSQQWRTWAACFIQ
AAWRRYSKKKLEESLREEENRLQDALARAGGSSPSLGATIYASRFAANALRALRRNSTRKARVPERIPPM
LLQKPAEPDFTSEER
>ZjCNGC2
MNSKGPKFVRFEDWKSEISVSYERESPTSNDGSYPGKAREHVKGVLRNIGRGFKRCYESIRSLRTKTLSS
RTTYATKRPEKDAASKKKIFDPQGAFLQQWNKIFVLSCVIAISLDPLFLYIPTIDTNQQCLSLDTPLEIT
ACVLRTFTDIFYVVHIIFQFRTGFIAPSSRVFGRGELIDDPVAIAKRYLSTYFIIDVLAILPLPQVVVLI
VIPQIKGPVALVTKDILEYVIFCQYVPRLIRIIPLYREVTRTSGLFTETAWAGAAFNLFLYMLASHVAGA
VWYLLAIERADRCWHDQFKNKPWDHSYLYCGEDRKDLPIADISLALNTSCPFIDPDEIKNSGIFNFGIFA
DALTSGIVDYDDFSTKIFYCFWWGLRNLSSLGQNLKTSTFVGEIIFAVCIAVFGLVLFSLLIGNMQKYLQ
STTVRVEEMRVRRRDAEQWMTHRMLPPNLKERIKRYEQYKWQETRGADEDNLIRNLPKDLRRDIKRHLCL
SLLTRVPMFEKMDEQLLDALCDRLKPVLYTEKSFVLREGDPVDEMVFIMRGNLATMTTNGGRTGFFNSVD
LKAGDFCGEELLTWALDPNSATSLPTSTRTVEALTEVEAFALMADDLKFVASQFRRLHSKQLRHDFRFYS
LQWRTWAACFIQVAWRRHCKRKLDKSLREAEDRLQNALANEVGSTPSLGATIYASRFAANALRTMRKNGA
VSARPPQRLLPLLPQKPAEPDFTAEGR
>ZjCNGC3
MVDLEERAVRDLEERAASKSEQSIIGKISKRMRPLKMPDFLEWNKIFALACVVAVSLDPLFIYVPVINEN
NKCLTMDKTLATTAVAVRFLADIIYVGDIIYNVNKSSLELKRRGTWKTNKFFKINNFFKNALAIWKLSWR
LILVDSMAILPIPQIIMLVSFKKVTGSGYLDERRLLSLILISQYVPRVFRIYISSKEATRVRDVLTDTVW
IRGAFNFFLYILASHVFGAFWYFFAVQRETACWFQACERSGIANCGLNTFCDDHESAPTNYITLVHSKCP
INVDPDDTIEELFDFGIFLDAIKSGLLGSYDFPRKLAYCFWWGLRNLSSLGSNLKTSSYFWETYFAISVS
IVGLLLFLYLIGNMQTYLQLATTRSEERRLKMKQKEPQIDAWISRNNLDKEKKTIMEKVQHSLEVDKDIN
VETLVNALPHEYRRYITRELCRGALKKYIILHQNNVNEEAIEKLVEAICEHMKPVAYKEKSNIIRVGDPC
QMVLITQGTVVVNTGSSGSNGGTCTNASAKQLNKDDIYGDNLLLTPEHDSPSGVTISNECVESLTEVEGF
AVNAKDLMNVISNSHWGLSCSILP
>ZjCNGC4
MFDCSYKSQYVGGQREKFVRLDDLDSRLSSSSDTGGRKCGFNIEGLSGAGRAGDTTSRSFKRGMRRGSEG
LKSIGRSLRFGVSRAVFPEDLKVSEKKIFDPQDKFLQLWNKLFVVSCILAVSVDPLFFYLPVINSSSNCL
GIDRKLAITATTLRTIVDAFYLIHIALQFRTAYIAPSSRVFGRGELVIDPAQIAKRYLRWHFIIDFLSVL
PLPQIVVWRFLARSNGSDVLSTKQALFFIVLIQYVPRLLRILPLTSELKRTAGVFAETAWAGAAYYLLLY
MLASHIIGALWYLLAVERNDTCWQKACKESNESSGTECITSFLYCGNQGIPGYDAWNRTNHPIFNGTCSG
DDGVDQFDFGIYTNALTSGIVSSNKLLSKYCYCLWWGLQNLSTLGQGLETSTYPGEVIFSIALAISGLIL
FALLIGNMQTYLQSLTIRLEEMRVKRRDSEQWMHHRLLPQDLRERVRRYDQYKWLETRGVDEESLVQSLP
KDLRRDIKRHLCLALVRRVPLFENMDERLLDAICERLKPSLFTEHTYIVREGDPVDEMLFIIRGRLESVT
TDGGRSGFFNRGLLKEGDFCGEELLTWALDPKSGSNLPTSTRTVKALTEVEAFALIAEELKFVAGQFRRL

HSRQVQHTFRFYSQQWRTWAACFIQAAWRRYSKRKTMELLRRKEEEEAEGSEGARTSTSGGTYSLGATLL
ASRFAANALRGVHRNRNAKTARELVKLQKPPEPDFSAEDAD
>ZjCNGC5
MFDSGYKSQYIGGQREKFVRLDDLDSTLSASASAVRMKRTRFNIEGLPFTSRSRKNAAKSFRFEMKKGSD
GLKTIGRSLKTGVTKMVFPEDLKVSEKMIFDPQDKSLLFWNRLLVISCIFAVSIDPLFFYLPVFNHKSNC
LGMDTSLSTTTTTLRTIMDSFYLVRMFFQFRTAFIAPSSRVFGRGELVIDSKEIANRYLHRYFFVDLLAV
LPLPQFVVWRYITKSDGSNVLSTKQALLKIVCFQFFPRFIRLIPLTSDLKKSAGAFAESAWAGAAYYLLW
FFLSGQITGAIWYLLAVERNDTCWRDACSRTGTCKIEYLYCDNKHVEGYRQWQKISKDVLNSRCSVFDDD
SSFNYGIYTQAISSRIVESRAFFSKFFYCLWWGVQNLSTLGQGLQTSTYPLEVLFSIAIGIAGLTLFALL
IGNIQTNLQSMTIRLEEMRIKRRDSEQWMHHRLLPQDLRERVRRYNQYKWLETRGVDEESIVHSLPKDLR
RDIKRHLCLNLVRRVPLFANMDERLLDAICERLKPSLYTEHTYIVREGDPVDEMLFIIRGRLESVTTDGG
RSGFFNRGFLKEGDFCGEELLTWALDPKAGSSLPSSTRTVNTLTEVEAFALEAEELKFVASQFRRLHSRQ
VQHTFRFYSQQWRTWAAIFIQAAWRRHSRRKLAEQRRKEEEEEDEEEFGYSKEEDRKALLARVSSTSRLH
ATFFASRFAANALRGHRLRDASSTSNIILRKPSEPDFSIYDAN
>ZjCNGC6
MEFKKEKLVRFQSDGKHHHKKSLWGRPEPVRLEKSSSTYKVSSSSLLKPDNGLFGDRSKFAETLRIGRSKV
FPEDHEPWRKRILDPGSEIVLQWNWVFIVSCLVALFIDPLYFYLPMVSVNDNSWCMKTDMNLRIIVTCLR
TVADFFYLLHMIIKFRTAYVAPSSRVFGRGELVMDPKKIAWRYIRSDFFIDLVATLPLPQMVIWFIIPAT
RSSRTDHNNNALALIVLLQYIPRLYLMFPLSSQIIKATGVVTKTAWAGAAYNLLLYMLASHVLGAAWYLL
SIDRYTSCWKSFCKKEVTPIKCDLHYLDCGSFNKNNLKEWFNATSVFDNCAANDESKFNYGIFENAVKKS
VVSSNFIEKYFYCLWWGLQNLSSYGQNLMTSTFIGETSFAILIAILGLVLFAHLIGNMQTYLQSMTVRLE
EWRLKQRDTEEWMRHRQLPEDLRRRVRRFVQYKWLATRGVDEESILHSLPADLRRDIQRHLCLDLVRRVP
FFSQMDDQLLDAICERLVSSLSTAGTYIVREGDPVTEMLFIIRGTLDSSTTNGGRTGFFNSITLRPGDFC
GEELLAWALLPKSTVNLPSSTRTVKALNEVEAFALRAEDLKFVANQFRRLHSKKLQHTFRFYSHHWRTWA
ACFIQAAWRRYKKRMMARDLMRESFAAMDGKEAYET
>ZjCNGC7
MAHGQSDSVRHHDDLELPKYTSNRGGHLIFKLISKVTGKIRADFRSRKVKKDEIGKAFRGKVLSRVFSED
YEVVEKLILDPRGPTVNRWNKIFLVACLISLYVDPLFFYLPVAKGTMCMDVCVTLEVVLTVIRSLVDAFY
IIQILVRFKTAYVAPSSRVFGRGELVIDPSKVASRYIHKDFWLDLVAAQPLPQVLIWIAIPHLRGSRVRS
TRHVLRLIILFQYLLRLYLIFPLSSKIIKATGVVTETAWAGAAYNLILYMLASHVLGSCWYLLAIERQEE
CWNKVCSLQHPECQYWYIDCHKVNDPGRAAWFKSSNISGLCGASSEFFDFGIYSDALKFTVIEPRFLNKY
FYCLWWGLRNLSSLGQNLLTSTYVGEINFAIIIAVLGLVLFGLLIGNMQTYLQSTTMRLEEWRIRRTDTE
KWMCHRQLPHELKQSVRKYEQYRWIATRGVDEEAVLKGLPMDLRRDIKRHLCLDLVRQVPLFNQMDERML
DAICERLKPSLCTPNTCLVREGDPVTEMLFIIRGHLDSCTTNGGQSGFFNLCHLGPGDFCGEELLTWALD
PRPSVVLPSSTRTVGSISEVEAFALIAEDLKFVAAQFRRLHSKQLRHTFRFHSHQWRTWAACFIQAAWFR
YKRRKEASELKKTQSFLVSSNAPGIEQTNTPLPSMGSGFSYVAKLAASTRRGGSKRCGHEFDMLSSLQKP
VEPDFTVEER
>ZjCNGC8
MGYDNSRSVRFQDDLELAKLPAVDGDGVVKLKYKIDGTQIPEPSSKKGEKEVFGKTTTSLKAKVLSRVFS
EDYERVKKKILDPRGPAIRRWSKIFLVTCLVSLFVDPLFFYLPVVQDEVCIDIGIPLEIILTIIRSLADI
FYVIQIFIKFRTAYIAPSSRVFGRGELVIDSSKIARRYFRQSFVIDLIAALPLPQVLIWMVIPNLSGSTM
TNTKNFLRFIIIFQYIPRLFLIFPLSSQIIKTTGVVTETAWAGAAYNLMLYMLASHVLGACWYLLSIERQ
EACWRSICHLENSLCQYKYFDCHWLKGLDRQTWFKLSNVTAQCNPDESYYDFGIYGDALTFDVTTSSFFN
KYFYCLWWGLRNLSSLGQNLSTSTYVGEITFAIIIATLGLVLFALLIGNMQTYLQSTTVRLEEWRIRRTD

TEQWMHHRQLPPELKQSVRKYDQYKWIATRGVDEETLLKGLPMDLRRDIKRHLCLELVRGVPLFDQMDER
MLDAICERLKPALCTEGTFLVREGDPVNEMLFIIRGHLDSFTTNGGRTGFFNSCKIGPGDFCGEELLTWA
LDPRPSVILPSSTRTVKALSEVEAFALVAEDLKFVASQFRRLHSKQLRHKFRFYSHQWRLWAACFIQAAW
RRYKKRKEVAELRAKESPTAAEPEQPKQESGLAMYAARLAASTRRGVNNKNHSGSDSGVVSLQKPTEPDF
SVCEE
>ZjCNGC9
MNRIISKRAAKFRPFRRLSSKIRDDAAAAATANEEEYPHLILWRYQILAPDSDIVAHWNHIFLVTCIVSL
FIDPLYFFLPSVGGPACLSTDTGQAITITCFRTVTDLFFILHIVMKFRTAFVAPSSRVFGRGELVMDPRE
IALRYLKKDFLIDLAAALPIPQIVIWFVIPATRNSRADHANNTLALFLLFQYVPRLFLIFPLNQRIIKTT
GVVAKTAWAGAAYNLLLYLLASHILGSAWYLLSIGRQFSCWQNECRKEHESKLVSCLYNFLDCKSKDQPE
RQYWLNVTKVISKCDARDNGIEFQFGMFADAFTNDVVSSPFIDKYLYCLWWGLRNLSSYATNVDTSRYIG
ETTFCIFTCIIGLILFSHLIGNMQTYLQSMTIRLEEWRIKRRDTEEWMRHRQLPPDLQERVRRFVQYKWL
TTRGVDEDSILRSFPLDLRREIQRHLCLALVRRVPFFSQMDGQLLDAICERLVSSLSIQGTYIFQEGDPL
DEMLFIIRGKLESSTTDGGRSGFFNSITLESGHFCGEELLTWALMPNPSINLPTSTRTVRALTEVEAFAL
RAEDLKFVAGQFKRLHSKRLQHAFRYYSHQWRTWGACYIQVAWRRYIKRKMAKDLSQQESFCYLNIPSQE
SNYADELEHGNYNYGTGDSLVEDNASHFQHLGATVLASRFAANTRRGVHHKAEGGSSASTSLKMPKLFKP
EEPNFSIDDEDEV
>ZjCNGC10
MELKKAEKLVRFYHDGKTDPHFTWMTNETQQHLEKPLPVYKVSGPILLKTEGGGVVDGNSINIVKFVKFK
VFQENHEPWQQRILDPGSDIFLQWNRVFLIFCLVALFLDPLFFYLPSVQNFGTTSCLETDLNLRIVVTCF
RTLADVFYLLHMVIKFRTAYVSPSSRVFGKGELVMDPKLIARRYLRSDFFIDLIAALPLPQILIWFTMPA
IRSSHSDHANNALVLIVLLQYVPRLYLIFPLSSEIIKATGVVTKTAWAGAAYNLLLYMLASHVLGASWYL
LSVERYATCWKSICRKELSPVRCFAKYLNCDTLDDGDRQKWINGTSVFSSCTPGDSTIFNYGIFENAVTN
NVVSSEFIEKYFYCLWWGLQNLSSYGQSLATSTFIGETSFAILIAILGLVLFAHLIGNMQTYLQSITVRL
EEWRLKRRDTEEWMQHRQLPQNLRERVRRFVQYKWLATRGVDEESILRGLPTDLRRDIQRHLCLDLVRRV
PFFAQMDDQLLDAICERLVSSLSTQGAYIVREGDPVTEMLFIIRGKLESSTTNGGRTGFFNSITLRPGDF
CGEELLAWALVPKSTLNLPSSTRTVRSLDEVEAFALRAEDLKFVANQFRRLHSKKLQHTFRFYSYHWRTW
AACFIQAAWRRYKKRTMAKILSMTESSYTLDPQVPGEMEREEEDEDTNSSPSSQAKQNLGVTILASMFAA
NTRRGAQKIKDVEMPKLQKPEDPDFTMEPDDE
>ZjCNGC11
MHSFPSYLPAPSSRFPHFSTPFSLRKKIPWWYQILDPGSDLVSRWNQIFLITCLIAMFIDPLYFYLPVIS
KSDSACMDIDINLGVLVTLLRTFTDFFYLLHITMKFRTAFIAPSSRVFGRGELVTDPRAIAIHYLKSDFF
IDFAATLPLPQIVIWFVIPALKNSTAAHANHTLSLIVLIQYIPRFFLIFPLNRRIIKTTGVIAKTTWAGA
AYNLLLYMLASHVTGASWYVLSIQRQYQCWKMECRKEMNGTHSPSCHASFLDCTNKDNPERDLWLGRTNI
VVHCDALNDDRNFDFGMFADAFTSQIAKSNFKEKYFYCLWWGLKSLSAYGQNIIASTRSAETLFSILICT
AGLILFSHLIGNMQSYLQSTSARLEEWRVKRKDTEEWMRHRQLPPELQNRVRRFVQYKWIATRGVDEEAI
LQDLPLDIRRQIQRHLCLALVRRVNFFAQMDNQLLDAICERLVSSLNTKDTYIVREGDPVNEMLFIIRGQ
LESSTTDGGRLGFFNCITLRPGDFCGEELLTWALMPTSNLSLPTSTRTVRSLTEVEAFALRAEDLKFVAS
QFKRLHSKRLQHAFRYYSHQWRTWGACFIQVAWRRYKKRKMTIDLARQEELYYGNNLDNESPYADYNESG
GHSSSRDDHRAQHLGATVLASKFAANTRKGVAHKVASLDPDATCLNMPKKLFKPEDPTFLADPDDS
>ZjCNGC12
MAAYXKDEMPMLSNNHSWSSDDNVNSPFERFGRTQSASFAISMNSTESYASEPKLVGHTGPLRSQRTSFI
PMSGPLNVHQPNNLLRPSRSVAVKKEAELLMVLELLMAIAKVLSQCRGVASTCISPKSSSTNRSHIACAV
ASDALIYSAPAVESATQLCLTETHENTPLPKLKAYPDFNSLQFHNAFYGDAKGWARRIFSFMSPYIPGVM

NPHAKVIQKWNKFFVIACLVAIFMDPLFFFLLSVRQDHKCIVINWPMTTVIVFFRSLYDIIYLLHILLQF
KLAYVAPESRVVGAGELVDHPKKIARNYLQGYFLLDFFNVLPLPQIVLLLIVRKSLSLSGVDYAKNLLRA
AILVQYGPRLYRFLPLLAGQSPSGFTFESAWANFNINLLIFVLSSHVVGSFWYLFGLQRVNQCLRNACHH
SDINYCMKFIDCGYGDNVDSFNKSYPIEWPNWTDNINANSCFSTDGFPYGIYIQAVNLTTERGVITRYVY
SLFWGFQQVSTLAGNQTPSYFVWEVLFTRLGLLLFALLIGNMQNFLQALGKRRLEMSLRCRDVEQWMSNR
RLPEELTRDIKQHRLNFSGMTIDLMVLESFTSHXLMEHVLKKKRFRMIPXVRIFSLMDEHILDAICERLR
QKMYIGGSKILYRGCLIEKMIFIVRGKMESIGEDGIGVPLSEGDVCGEELFTWCLEHSSVNKDRKKVRLP
GQRLLSTRLVQCLTNVEVFSLRAADIEEVTSLFSGFLRNPRVQGSIRYESPYWRGLAAIHIQVTWRYRKK
RLSRAKISQSDHSSTQ
>ZjCNGC13
MATEHEISQTTSHMHYNLSDCSDSEEVEEEEANQEEEDEDDNGSEISSVAGEGGFCNSLAYVCGGVGRRR
RKPKGWSLGQVLDPRAKWVQEWNRVFLLVCATGLFVDPLFFYALSISDTCMCLFVDGWFAITVTVLRCMT
DALHVWNMWLQLKMAKRSFSLVGVEGRNGLGDTTARSVALRYLKAKKGFLFDLFVILPLPQIVLWVAIPS
LLERGSITNVMTVFLIIFLFQYLPKIYHSVCLLRRMQNLSGYIFGTVWWGIALNMIAYFVASHAAGACWY
LLGIQRAAKCLKEQCRATSGCGMRILSCKEPIYYGTTSMVKDRARLAWAVNKQARSTCLENSDNYDYGAY
KWTVQLVTNDSRLEKILFPIFWGLMTLSTFGNLESTTEWLEVVFNIIVLTSGLLLVTMLIGNIKVFLHAT
TSKKQAMQLKMRNIEWWMRKRHLPLGFRQRVRNYERQRWAAMRGVDECEMIRNLPEGLRRDIKYHLCLDL
VRQVPLFQHMDDLVLENICDRVKSLIFTKGETITREGDPVHRMLFVVRGHLQSSQLLRDGIKSCCMLGPG
NFVGDELLSWCLRRPFIERLPPSSSTLITLETTEAFSLEAEDVKYVTQHFRYTFVNEKVKRSARYYSPGW
RTWAAVAIQLAWRRYKHRLTLTSLSFIRPRRPLSRCSSLGEDRLRLYTALLTSPKPNQDDFDF
>ZjCNGC14
MFSISRWTELFQRKSFQNRNSSVSGSRNDNDIVVVVPKECYQCTQAGIPFFHSTLCPDPSHHPHWEASAG
SSFRPAPTTTVPTRTHSQAQDHHHHHHDPLFPLGRVVDPRSKTVKRWNRVVLLARGMALAVDPLFFYVVW
MSGDGSPCFYMDVALAMIVTVVRTCLDAMHLGYVWLQFRMAYVSNESMVVGCGKLVWDARAIASRYLRSL
RGFWLDAFVILPIPQIVIWIVVPKLLREEQIKWITRTFLLSFLFQFLPKIYHSIYLMKKLQKVTGYLFGS
IWWRFNLNAIAYLIASHVAGACWYVLATERLVSCLQQQCAISRKCNLNLYCNNSQAFGNTDGVNYSSINS
KCLDVDGPFNYGIYNPVLLVFSSNSLAVRILYPVFWGLLNLSSFGNELEPTCNWLELIFSCCITLAGLIL
FVTLIGNIQVFLHTVMANKKKMQLKYRDMEWWMKRRQLPNPLRDRVRNFQRHSWSAMEGQDEMELIQH
LPDGLRRDIKRFLCIDLVKKVPLFHMLDDLILDNICDRVKPLIYSAGEKIIREGDPVQRMVFIVRGRVNRRQ
GLSKGFIANSVVEPGGFFGDELLSWCLRIPAANWLPVSLATYTCIDSAEGYAIDAHQLRYVVDHFRYKFA
SESLKKTARYYSCNWRTWGAVVIQLAWRRRRLRNGTNLPILQNGDPEDRLRQYAAFFLSIRPHDHLE
>ZjCNGC15
MPSHPNPFHFSIQRWINNGRQKQTNQNTSQSDSDDNDDDSPISSSVECYACTRMGAPVFHSTTCDKANQP
QWEACAGTSLIPIHSRTDLKKGLGSAARLQRPTGPFGRVLDPRSKRVQKWNRTFLLARGMALAVDPLFFY
ALSIGRGGTPCLYMDGGLAAIVTVLRTCVDAVHLCHLWLQFRLAYVSRESLVVGCGKLVWDTRAIATHYL
RSFKGFWFDAFVILPVPQAVFWLVLPKLIREERIKLIMTILLLIFLFQFLPKVYHSICLMRRMQKVTGYI
FGTIWWGFGLNLIAYFIASHVAGGCWYVLAIQRVASCLRQHCERSTKCNLSLSCSEEVCYQFLLRSDTVG
NPCGGNSTVARRPLCLDVNGTFNYGIYQWALPVISSNSVSVKILYPIFWGLMTLSTFGNDLEPTSHWLEV
IFSICIVLSGLLLFTLLIGNIQVFLHAVMAKKRKMQLRCRDMEWWMRRRQLPSRLRQRVRHFERQRWAAM
GGEDELEMLKDLPEGLRRDIKRYLCLDLIKKVPLFHNLDDLILDNICDRVRPLVFSKDEKIIREGDPVPR
MIFIVRGKIKRSQNLSKGMVATSVLEPGGFLGDELLSWCLRRPFNDRLPSSSATFLCVESTDAFGLNSED
LRYITDHFRYKFANERLKRTARYYSSNWRTWAAVNIQLAWRRYRLRTRGPVIPAVSTDNGGTDRKLMQYA
ALFMSIKPHDHLE
