# Supplementary material for: Genome-wide identification of CNGC genes in Chinese jujube (Ziziphus jujuba Mill.) and ZjCNGC2 mediated signalling cascades in response to cold stress
Source: BMC Genomics. 2020 Mar 2;21:191. doi: 10.1186/s12864-020-6601-5 (PMC7053155; doi:10.1186/s12864-020-6601-5)
Supplement: Supplementary file 2 — Additional file S2. The CDS sequences of ZjCNGCs. [file 12864_2020_6601_MOESM2_ESM.pdf]

>ZjCNGC01
ATGAATTTCCAGCAAGATAAATTTGTGAGGTTTCAGGATTGGAATTCAGAGAAAAACACCGGGGTGTTAT
ATTCTCCAAATAATGATACACACCCAGGAAGAATCAGAACAACGATAGACACAGTTTCAGACAAGTTTCA
AAGAGGATTGGAATCTAGTTCTGAGAGGATTAAAAGGATTAAGCAATCCTTGAAATCATGTTCTTTTGGT
AGTGTCATGGCCAAAAGCTGGGTTTCCCAGAAGAAAGTTCTTGATCCACAAGGGCCATTTCTTCAGAAGT
GGAATAAGATATTTGTGCTCTCATGCGTGATTGCAGTTTCTTTGGATCCTTTGTTCTTTTATGTCCCAGT
AATTGATGATAAGAGAAAGTGCTTAGACTTGGACGGCAAGATGGAAATCACAGCAAGTGTTTTGCGTTGG
TTCACAGATTTATTTTATATAACACATATAATTTTTCAGTTTCGTACTGGGTTTATTGCTCCCTCTTCTC
GAGTATTTGGAAGAGGTGTTTTAGTTGAAGATCCTTGGGCCATAGCAAAGAGATATCTGTCAACTTACTT
CCTAATTGACATTCTTGCTGTCCTTCCTCTCCCGCAGGTGGTGATTTTAATTATCATTCCAAAATTGGGA
GGCTCAACATCGTTGAATACAAAGAACTTGTTGAAATTTGTTGTTTTGTTCCAATATATACCGAGGGTCA
TTCGAATATATCCACTGTATACAGAAGTTACAAGGACATCGGGCATACTCACTGAAACTGCCTGGGCAGG
AGCTGCATTTAATCTTCTACTTTACATGCTTGCAAGTCATGTACTCGGAGCCTTTTGGTACTTGTTTTCT
ATCGAACGACAAACCAAATGCTGGCAAGATGCCTGCGAGATGAATAACACTTTATGTAACACTGCTTCTT
TGTATTGCGAAAACTCGAGTAACATACAATTGCGGACATTTTTGAATGATTCCTGTCCAATACAAGAAGA
TAGTCCAAAATTCAATTTCGGAATATCTCTTGATGCCCTTCAATCTCATGTTGTTGATTCAACGACTGAT
TTTCCTCAGAAGTTGTTCTATTGTTTCTGGTGGGGCCTGCGAAATTTGAGTTCTCTTGGTCAAAACCTTG
AAACTAGTACCTATGTCTGGGAAATCTGCTTTGCAGTCTTCATTTCCATTGCTGGGTTGGTGCTATTCTC
ATTCCTTATAGGAAATATGCAGACATATTTACAGTCCACAACTACAAGATTGGAGGAGATGAGAGTGAAA
AGAAGAGATGCAGAACAATGGATGGCCCACCGATTGCTCCCTGAGAATCTGAGGGAACGCATTAGGCGGT
ATGAACAATACAAATGGCAGGAAACCAGGGGTGTGGATGAAGAGAATCTAATCTGTAATCTTCCAAAGGA
TCTTAGAAGGGACATAAAGCGCCATCTCTGCTTGGCTTTGCTCATGAGAGTGCCAATGTTTGAAAAATTG
GATGAACAGTTACTGGATGCATTGTGTGACCGTCTCAAGCCAGTGCTTTACACAGAGGAAAGCTACATTG
TTCGAGAGGGAGACCCAGTTGATGAGATGCTTTTCATTATGCGAGGCAGGCTACTGACTATGACTACGAA
TGGTGGAAGAACAGGTTTCTTCAACTCTGAATATCTCAAAGCCGGTGACTTCTGTGGAGAAGAACTTCTT
ACATGGGCATTGGATCCCCACTCCTCATCTAATCTTCCTATCTCAACCAGAACTGTCCAAGCCCTCACAG
AAGTTGAAGCCTTTGCATTAAAGGCAGAAGATTTGAAGTTTGTAGCCTCTCAATTCCGGCGGCTTCACAG
CAAGCAGCTCCGCCACACTTTCAGGTTTTACTCTCAGCAGTGGAGGACTTGGGCTGCTTGTTTCATTCAA
GCAGCATGGCGCCGCTATAGTAAGAAGAAACTCGAAGAGTCTCTTCGTGAAGAGGAGAATAGGTTGCAAG
ATGCACTGGCCAGGGCTGGTGGCAGCTCACCAAGTTTAGGTGCCACCATCTATGCCTCACGATTTGCTGC
TAATGCACTCCGTGCTTTAAGGCGTAATAGTACACGGAAGGCAAGGGTTCCTGAGAGAATACCACCCATG
TTGCTTCAGAAGCCTGCAGAGCCTGATTTCACTTCGGAAGAACGATAGTTACAAGAGAGTAGCGATTGAT
TATATGCATGTTTTTGTTGTATAACATGTAAAATGCAACTATTTTAAGATATAAGCTACAGTTTGTTAAG
TGTCCAAGGTCTGTATATTATCTATTTTTTTATATGGAAATTCCCGATTGTTTTTTTGGAGTTA
>ZjCNGC02
ATGAATTCCAAGGGACCTAAATTTGTAAGGTTTGAAGATTGGAAATCAGAAATCTCTGTAAGTTATGAAAGG
GAATCACCCACAAGTAATGATGGGTCATATCCAGGAAAAGCCAGAGAACATGTGAAAGGAGTTTTGAGAA
ATATTGGGAGAGGTTTTAAGAGATGTTATGAGAGCATTAGAAGCCTGAGAACAAAAACATTAAGCTCTCG
TACTACTTATGCGACTAAGCGGCCGGAAAAAGATGCGGCATCTAAGAAAAAAATTTTCGACCCGCAAGGA
GCTTTTCTTCAGCAATGGAACAAGATATTTGTGCTCTCTTGTGTTATAGCAATATCGTTGGACCCGTTGT
TTTTGTACATTCCCACCATTGATACCAACCAGCAATGCCTCAGTCTGGACACACCGTTGGAGATCACTGC
CTGTGTTCTTCGAACATTTACCGATATCTTTTATGTTGTTCATATTATCTTTCAATTCAGAACCGGGTTT
ATTGCCCCTTCTTCTAGAGTATTCGGAAGGGGTGAGTTAATTGATGATCCTGTGGCTATAGCAAAAAGAT
ACTTATCCACCTATTTCATCATCGATGTTCTAGCAATTCTCCCACTGCCACAAGTGGTAGTTTTGATTGT

CATACCTCAGATTAAAGGTCCAGTTGCACTGGTCACAAAGGACATTTTGGAGTATGTAATTTTCTGTCAG
TATGTGCCAAGACTTATAAGGATTATTCCACTATACAGAGAAGTAACTAGAACTTCTGGCCTTTTCACTG
AGACAGCATGGGCTGGAGCTGCTTTCAATCTCTTTCTCTATATGCTTGCTAGTCATGTGGCTGGAGCAGT
TTGGTACTTGCTCGCCATTGAGCGAGCAGATAGGTGTTGGCATGATCAATTTAAGAACAAGCCTTGGGAC
CACAGTTATTTATACTGCGGTGAAGACAGAAAAGACTTACCGATTGCAGACATTAGTTTAGCACTAAATA
CTTCTTGCCCTTTCATTGATCCTGATGAGATTAAAAATTCTGGCATCTTTAACTTTGGAATATTTGCTGA
TGCTTTGACATCTGGCATTGTTGACTACGATGATTTTTCTACCAAGATTTTCTACTGTTTTTGGTGGGGA
CTGCGCAATCTTAGTTCTCTTGGCCAGAACTTAAAAACAAGCACTTTCGTGGGAGAAATAATTTTTGCAG
TCTGCATCGCTGTCTTTGGATTGGTTCTATTTTCATTACTCATTGGGAATATGCAGAAATATCTGCAATC
CACAACTGTGAGAGTTGAAGAAATGAGAGTGAGAAGACGAGATGCAGAGCAGTGGATGACTCACCGCATG
CTTCCTCCAAACTTGAAGGAACGCATTAAAAGATATGAACAGTACAAATGGCAAGAAACAAGAGGTGCTG
ACGAAGATAATCTAATTCGTAACCTTCCAAAGGACCTTAGGAGGGACATAAAGCGTCACCTTTGTCTTTC
TCTTCTCACTAGAGTACCAATGTTTGAAAAAATGGATGAACAACTCTTGGATGCTTTGTGTGATCGTCTC
AAACCAGTTTTGTACACAGAGAAAAGTTTCGTTCTCCGGGAGGGTGATCCTGTTGATGAAATGGTCTTTA
TAATGAGAGGAAATCTAGCAACAATGACTACCAATGGTGGAAGAACTGGCTTTTTCAACTCAGTTGATCT
TAAGGCTGGTGACTTCTGTGGAGAAGAGCTACTTACATGGGCCTTGGATCCCAACTCTGCCACCAGTCTC
CCTACATCCACCAGGACAGTGGAAGCTCTAACAGAAGTAGAGGCTTTTGCTCTCATGGCTGATGATTTGA
AGTTCGTTGCCTCCCAATTCAGGAGGCTTCACAGTAAACAACTCCGGCACGATTTCAGATTCTACTCCCT
GCAATGGAGGACATGGGCTGCCTGTTTTATACAAGTGGCATGGCGGCGACATTGCAAGAGGAAGCTTGAC
AAGTCTTTACGTGAAGCAGAAGACAGGCTGCAAAATGCTTTGGCAAACGAAGTTGGGAGCACACCAAGTC
TTGGTGCTACTATTTATGCATCAAGGTTTGCTGCCAATGCACTGCGAACCATGCGAAAAAATGGTGCTGT
AAGTGCCAGACCACCACAGAGATTGCTACCATTGCTGCCTCAAAAACCAGCTGAGCCTGATTTCACTGCA
GAAGGTCGTTAG
>ZjCNGC03
ATGGTTGATCTGGAAGAACGAGCTGTGAGGGATCTGGAAGAAAGAGCTGCGAGCAAATCAGAACAGAGT
ATCATTGGAAAAATTTCAAAGAGAATGCGTCCTTTAAAGATGCCAGACTTTCTAGAGTGGAATAAGATAT
TTGCATTGGCATGTGTGGTTGCGGTCTCACTGGATCCTTTGTTCATTTATGTTCCTGTCATCAATGAAAA
TAATAAATGTCTTACCATGGACAAGACATTGGCGACAACAGCTGTTGCTGTCAGATTCTTAGCAGATATA
ATTTATGTCGGAGATATTATCTATAATGTTAATAAATCTTCTCTGGAATTGAAAAGACGTGGGACATGGA
AAACAAACAAGTTTTTTAAGATAAACAACTTTTTTAAGAATGCTTTGGCAATATGGAAGCTTTCCTGGCG
TCTTATACTTGTTGACTCTATGGCTATTCTTCCGATTCCACAGATAATAATGTTAGTCTCCTTTAAGAAA
GTGACGGGCTCGGGATATTTGGATGAAAGGAGGTTACTGAGCTTAATTCTTATATCCCAATATGTTCCAA
GGGTTTTTCGAATATACATATCATCTAAGGAAGCTACAAGGGTTCGGGACGTCCTCACGGATACTGTTTG
GATTAGGGGTGCTTTTAACTTTTTTCTCTACATTCTCGCAAGCCATGTGTTTGGAGCCTTTTGGTACTTT
TTCGCTGTTCAAAGAGAAACAGCATGCTGGTTCCAAGCCTGTGAACGATCAGGAATAGCAAACTGTGGAC
TCAATACTTTTTGTGATGATCATGAAAGTGCACCAACAAATTATATCACACTTGTACATTCTAAATGTCC
CATAAACGTGGATCCCGATGATACAATAGAGGAACTCTTCGATTTTGGGATATTCCTGGATGCCATCAAG
TCTGGTCTGCTGGGATCATATGATTTTCCACGAAAGTTGGCTTATTGTTTTTGGTGGGGACTGCGGAATT
TGAGTTCTCTTGGTTCAAACCTCAAAACAAGCAGTTACTTTTGGGAAACGTACTTTGCAATTTCTGTTTC
TATTGTTGGCTTGCTACTGTTCTTGTATCTCATTGGAAACATGCAGACTTATCTACAATTGGCGACTACA
AGATCCGAGGAGAGAAGACTAAAGATGAAACAGAAAGAACCTCAGATTGATGCGTGGATATCAAGAAATA
ACCTTGATAAAGAGAAGAAGACGATCATGGAAAAAGTACAACACAGTTTGGAAGTAGACAAAGATATTAA
CGTCGAAACTCTGGTCAATGCACTCCCTCATGAATATAGGAGGTATATAACGCGCGAACTCTGCCGTGGT
GCGCTTAAAAAATACATTATTTTGCATCAAAATAATGTAAATGAGGAGGCAATAGAGAAATTGGTGGAGG

CGATATGCGAACATATGAAGCCGGTAGCATACAAAGAGAAAAGTAACATCATAAGAGTAGGAGACCCATG
TCAGATGGTCCTCATAACACAAGGCACTGTTGTGGTGAACACAGGAAGCAGCGGATCAAATGGTGGCACT
TGTACTAATGCTAGTGCGAAACAACTGAACAAAGATGATATATATGGAGATAATTTGTTGCTCACGCCGG
AGCATGATTCGCCTTCAGGTGTCACAATCTCAAACGAATGTGTAGAATCGCTTACAGAAGTTGAAGGCTT
TGCTGTCAATGCCAAGGACTTGATGAATGTGATCTCTAACAGCCACTGGGGGCTTAGCTGCTCGATTCTC
CCCTGA
>ZjCNGC04
ATGTTTGATTGCAGTTACAAGTCACAGTACGTGGGTGGCCAACGAGAGAAGTTTGTGCGGTTGGATGA
CTTGGACTCAAGATTATCATCATCTTCTGATACGGGAGGAAGAAAATGTGGATTTAATATTGAGGGTCTA
AGTGGTGCTGGACGTGCAGGTGATACAACATCTAGATCTTTTAAGAGAGGGATGAGAAGGGGATCTGAAG
GACTGAAGTCAATTGGCCGATCACTTAGATTTGGTGTTTCTCGGGCAGTGTTCCCAGAAGACCTTAAAGT
GTCAGAGAAGAAGATATTCGACCCTCAAGACAAATTTCTTCAATTATGGAATAAACTTTTTGTTGTATCA
TGTATTCTGGCAGTATCTGTGGACCCTCTTTTCTTTTATCTTCCAGTTATCAACAGTTCATCAAATTGTC
TTGGTATTGATAGAAAATTAGCAATCACTGCAACAACATTGCGGACAATAGTTGATGCTTTCTACCTTAT
TCACATAGCTCTCCAGTTCCGAACAGCATATATTGCTCCTTCATCCCGGGTTTTTGGGCGAGGTGAACTT
GTGATAGATCCAGCACAAATAGCTAAGAGATACTTGCGCTGGCATTTCATCATTGATTTTTTATCTGTGC
TACCCCTACCGCAGATTGTGGTTTGGAGATTTCTTGCAAGGTCCAATGGTTCTGATGTGCTTTCTACGAA
GCAAGCCTTGTTTTTCATTGTCTTAATTCAGTATGTTCCTAGACTGCTGCGAATCCTACCTTTAACTTCA
GAACTGAAAAGAACAGCTGGTGTTTTTGCTGAGACAGCATGGGCAGGTGCTGCATACTACTTGCTGCTGT
ACATGCTTGCTAGCCATATAATTGGTGCTTTATGGTATTTGTTAGCTGTCGAGCGCAATGATACATGCTG
GCAGAAGGCTTGTAAAGAAAGTAATGAGAGTAGTGGGACTGAATGCATAACAAGTTTCTTATATTGCGGC
AACCAAGGTATACCAGGTTATGATGCTTGGAACAGAACCAATCATCCAATTTTCAATGGAACATGTTCAG
GAGATGATGGGGTTGATCAATTTGATTTTGGAATCTATACAAATGCTTTGACATCTGGTATTGTTTCGTC
GAACAAGTTACTTTCTAAATACTGTTACTGTTTATGGTGGGGACTACAAAATTTGAGTACACTTGGTCAG
GGGCTCGAGACCAGCACCTATCCTGGAGAGGTTATATTTTCCATAGCACTGGCTATATCTGGACTTATCC
TCTTTGCGCTTTTGATTGGCAACATGCAGACCTATCTTCAGTCCCTTACTATACGGCTTGAGGAAATGAG
GGTCAAAAGGCGGGATTCAGAGCAGTGGATGCATCATCGCTTGCTCCCACAAGACCTCAGGGAGCGGGTT
AGGCGTTATGACCAATACAAATGGTTGGAAACACGTGGGGTTGACGAAGAGAGTTTGGTTCAAAGTCTAC
CCAAGGATCTTAGAAGAGATATCAAGCGGCACCTCTGTCTTGCTTTAGTGAGGAGGGTTCCTCTGTTCGA
GAATATGGATGAGAGGCTGCTGGATGCCATTTGTGAGCGGCTAAAACCGAGTTTATTCACAGAGCATACT
TACATAGTTAGGGAAGGAGATCCAGTAGATGAGATGCTTTTCATCATTCGTGGTCGCCTTGAGAGTGTAA
CCACAGATGGCGGAAGAAGTGGGTTCTTCAACCGCGGTTTGCTAAAAGAAGGTGATTTCTGTGGTGAGGA
GCTACTGACCTGGGCGCTGGATCCAAAATCTGGTTCTAATCTCCCTACATCCACTCGGACAGTAAAGGCA
TTAACAGAGGTTGAGGCATTTGCCCTGATAGCTGAAGAGTTGAAATTTGTGGCTGGTCAGTTCAGGCGCC
TTCACAGTAGACAAGTTCAACACACCTTCCGTTTCTATTCGCAGCAGTGGAGGACTTGGGCTGCTTGCTT
CATCCAAGCAGCATGGCGTCGCTATTCCAAGAGAAAAACCATGGAACTCCTCCGTAGGAAGGAAGAGGAA
GAAGCAGAAGGTTCAGAGGGGGCTCGCACCAGTACCAGTGGAGGTACATATAGTCTAGGTGCCACTTTGT
TAGCTTCCAGGTTTGCGGCAAATGCTCTTCGTGGAGTTCATCGGAATCGGAATGCGAAGACCGCTAGGGA
GTTGGTGAAATTACAGAAGCCCCCAGAGCCTGATTTCAGTGCTGAAGATGCAGATTGA
>ZjCNGC05
ATGTTTGATTCTGGTTACAAGTCTCAGTATATTGGCGGTCAAAGGGAGAAATTCGTTAGGTTGGACGATTTA
GATTCTACATTGTCAGCGTCAGCATCAGCTGTTAGGATGAAGAGAACCAGATTTAATATAGAGGGCTTGCCT
TTCACTTCTCGTTCGAGAAAGAATGCAGCAAAATCCTTCAGGTTCGAAATGAAGAAAGGGTCAGATGGACT
GAAAACCATAGGTAGATCATTAAAGACTGGTGTCACTAAGATGGTTTTCCCAGAAGATCTTAAAGTTTCTGA

GAAAATGATATTTGATCCTCAAGACAAGTCTCTCCTGTTTTGGAATAGACTGTTGGTCATTTCATGCATTTTTG
CAGTTTCTATTGATCCTTTGTTTTTCTATCTTCCTGTCTTCAATCATAAATCAAATTGCCTTGGTATGGATACAAG
TCTATCAACCACAACAACTACTCTGCGGACAATAATGGATTCATTCTACCTCGTTCGCATGTTTTTTCAATTCC
GAACAGCTTTTATAGCACCATCTTCTAGAGTTTTTGGAAGAGGTGAACTTGTAATTGATTCTAAAGAGATAGC
TAATCGGTATCTTCATCGTTATTTCTTTGTTGATTTGTTAGCTGTCCTACCCTTACCTCAGTTTGTGGTATGGAG
ATATATTACGAAATCAGATGGTTCCAATGTATTGTCTACAAAACAGGCATTGCTAAAAATTGTGTGCTTTCAGT
TCTTTCCAAGATTTATTCGATTGATTCCTTTAACTTCAGATCTTAAGAAGTCAGCTGGTGCTTTTGCTGAAAGT
GCTTGGGCTGGTGCTGCTTACTATTTGCTATGGTTTTTCCTTTCTGGTCAAATCACAGGGGCTATTTGGTA
CTTATTAGCTGTGGAACGTAATGACACATGCTGGAGAGATGCTTGTTCTAGAACAGGAACATGTAAGATA
GAATATTTGTACTGTGACAATAAGCATGTGGAAGGCTATAGACAATGGCAAAAAATAAGTAAGGATGTTC
TTAATAGCCGCTGCTCTGTGTTCGATGATGATTCATCTTTTAATTACGGCATCTATACACAAGCTATATC
GTCTCGCATTGTTGAATCCAGGGCTTTTTTCTCCAAATTTTTTTATTGTTTGTGGTGGGGAGTGCAAAAT
TTGAGTACACTTGGTCAAGGGCTTCAAACAAGTACCTATCCTTTAGAGGTTCTCTTTTCCATAGCTATAG
GCATTGCTGGCCTCACCCTTTTTGCGCTTCTAATTGGAAATATACAGACAAACCTTCAGTCCATGACAAT
TCGTCTTGAAGAAATGAGGATCAAAAGGCGTGACTCTGAGCAGTGGATGCATCACCGCTTGCTTCCACAA
GATCTTAGGGAAAGAGTTCGACGCTATAATCAATACAAGTGGTTGGAGACTCGAGGTGTAGATGAGGAGA
GCATAGTCCATAGTCTACCCAAAGATCTCAGGAGGGACATCAAACGACATCTTTGTTTGAATTTGGTGAG
ACGGGTTCCTCTTTTTGCCAATATGGATGAGCGGTTACTTGATGCCATTTGCGAGCGTTTAAAGCCAAGT
TTATACACTGAACATACGTATATAGTCCGAGAAGGTGATCCAGTTGATGAGATGCTATTCATCATCCGAG
GCAGGTTAGAAAGTGTGACTACAGATGGTGGAAGGAGTGGATTTTTCAACAGAGGTTTTTTAAAAGAAGG
TGATTTCTGCGGAGAAGAGCTTTTAACATGGGCCCTGGACCCGAAAGCTGGTTCGAGTTTGCCATCTTCT
ACACGAACTGTGAATACTTTAACTGAGGTGGAGGCTTTTGCCTTGGAGGCAGAGGAGTTGAAGTTTGTTG
CCAGTCAATTTAGACGTCTTCATAGCAGACAGGTTCAGCATACATTTCGTTTCTACTCCCAGCAGTGGAG
AACGTGGGCTGCCATTTTTATCCAAGCTGCGTGGCGGCGGCATTCGAGGAGAAAATTGGCAGAGCAACGG
CGTAAAGAAGAGGAGGAAGAAGATGAAGAAGAGTTTGGTTATAGTAAGGAAGAAGATAGGAAGGCTTTA
CTTGCTAGAGTTAGTAGCACATCGAGGCTTCATGCCACTTTTTTCGCTTCCCGTTTTGCAGCAAATGCTCT
TCGTGGTCATAGGCTTCGCGATGCAAGTAGCACCAGTAATATAATTCTGAGGAAGCCTTCTGAACCTGAC
TTCTCGATTTATGATGCTAATTGA
>ZjCNGC06
ATGGAATTCAAGAAGGAAAAGCTTGTCAGGTTTCAGTCAGATGGAAAACACCATAAAAAATCTTTATGGGG
AAGACCTGAGCCAGTGCGGCTTGAAAAATCATCATCTACATATAAGGTTTCATCGTCTTCATTACTGAAACCT
GATAATGGACTGTTTGGGGACAGAAGTAAATTTGCTGAAACTCTCCGAATTGGGAGGTCTAAAGTGTTTCCA
GAGGATCATGAGCCTTGGCGCAAGAGAATTCTTGACCCAGGTAGTGAGATTGTGTTGCAATGGAACTGGGT
TTTCATTGTCTCATGCTTGGTGGCGCTTTTTATTGATCCGTTATATTTTTACTTGCCTATGGTGAGTGTGAATGA
TAACTCATGGTGTATGAAGACAGACATGAATTTGCGAATTATTGTCACTTGTCTTAGGACTGTCGCGGATTTT
TTTTATTTGTTGCATATGATTATAAAGTTCAGAACTGCTTATGTTGCACCAAGCTCTCGAGTATTTGGGAGAGG
TGAGCTTGTCATGGATCCAAAGAAGATTGCTTGGAGGTATATTAGATCTGATTTCTTCATTGATCTAGTTGCCA
CATTGCCTCTCCCTCAGATGGTCATATGGTTTATTATACCTGCAACGAGAAGCTCGAGAACTGATCATAACAAC
AATGCCCTTGCATTAATTGTCCTACTCCAATATATTCCCAGATTATATTTGATGTTTCCATTAAGTTCTCAA
ATAATTAAAGCAACTGGAGTGGTCACAAAGACTGCCTGGGCAGGAGCTGCATATAATCTGCTATTGTACA
TGTTGGCTAGTCATGTTTTAGGGGCAGCTTGGTATTTACTGTCAATTGACCGATATACCTCATGCTGGAA
ATCCTTTTGTAAAAAGGAAGTTACCCCAATAAAATGTGATTTGCACTATTTAGATTGTGGTTCTTTCAAC
AAGAATAATCTGAAGGAATGGTTCAATGCCACAAGCGTTTTTGATAACTGTGCTGCCAATGATGAAAGCA
AATTCAATTATGGGATATTTGAAAATGCAGTGAAAAAAAGTGTTGTTTCCTCAAACTTCATTGAAAAGTA

TTTCTATTGTTTATGGTGGGGCTTACAAAACTTGAGTTCATATGGGCAAAATTTGATGACAAGCACTTTT
ATTGGAGAGACATCATTTGCAATTCTCATAGCCATTTTGGGTCTTGTATTATTTGCTCACTTGATTGGGA
ATATGCAGACATATCTGCAATCTATGACTGTGAGGCTTGAGGAGTGGAGGCTTAAGCAAAGAGACACAGA
GGAATGGATGAGGCATCGCCAACTTCCTGAAGATTTGAGAAGGCGAGTTCGACGTTTTGTTCAATATAAG
TGGCTTGCAACTCGAGGAGTTGATGAAGAATCCATCCTTCATAGCTTACCTGCTGATCTTCGTCGTGACA
TCCAACGTCACCTATGCCTAGATCTGGTCCGCCGGGTACCATTCTTTTCCCAGATGGATGATCAGCTGCT
CGATGCAATATGCGAGCGTCTAGTTTCCTCTCTAAGCACCGCAGGCACCTACATTGTTCGTGAGGGTGAC
CCTGTAACGGAGATGCTTTTCATTATCAGAGGTACACTGGATAGTTCCACTACCAACGGAGGCAGGACTG
GTTTCTTCAACTCAATCACATTGAGACCGGGAGATTTTTGTGGGGAGGAGCTACTTGCATGGGCATTGCT
CCCAAAATCCACTGTCAACTTGCCCTCTTCAACTAGAACGGTTAAAGCACTTAATGAAGTTGAAGCTTTC
GCATTGCGAGCTGAAGATCTCAAGTTTGTGGCCAACCAGTTTAGGCGCCTCCACAGCAAGAAGCTTCAAC
ATACCTTCCGGTTTTACTCCCACCATTGGAGGACATGGGCAGCGTGCTTCATACAGGCTGCTTGGCGTCG
GTACAAGAAGAGGATGATGGCGAGGGACTTAATGAGGGAATCATTTGCTGCCATGGATGGGAAAGAGGCT
TATGAGACT
>ZjCNGC07
ATGGCTCATGGTCAATCAGATTCTGTAAGACATCATGATGATCTTGAATTGCCAAAGTACACATCAAACAGAG
GAGGTCATTTGATATTTAAACTGATCTCCAAAGTCACTGGAAAAATTAGAGCTGACTTTAGATCAAGGAAGG
TTAAAAAGGATGAGATAGGGAAGGCCTTTCGTGGAAAAGTGTTATCTAGAGTCTTCTCTGAGGATTATGAG
GTAGTGGAGAAGCTGATATTGGATCCTCGAGGACCTACTGTGAACAGATGGAACAAGATTTTCTTAGTAGC
ATGTTTGATTTCTCTTTATGTGGACCCTCTCTTTTTTTACTTGCCAGTGGCAAAGGGTACCATGTGCATG
GACGTATGTGTAACCCTTGAAGTCGTTCTTACTGTCATTAGGTCACTGGTTGATGCATTTTACATTATTC
AGATTCTTGTTCGGTTCAAAACAGCTTATGTTGCTCCATCCTCTCGTGTATTTGGGAGAGGAGAGTTGGT
TATTGACCCTTCAAAGGTGGCTTCAAGGTATATTCACAAGGATTTCTGGCTTGACCTTGTGGCTGCCCAA
CCCCTTCCACAGGTTTTGATTTGGATAGCAATACCACATTTAAGGGGTTCAAGAGTGAGAAGTACTAGAC
ATGTCCTTCGCTTAATTATCTTATTTCAGTATCTCCTGAGGCTTTATCTTATCTTTCCCCTTTCATCTAA
AATTATCAAGGCTACCGGGGTTGTTACAGAAACAGCATGGGCTGGGGCAGCGTATAATCTGATTCTCTAC
ATGTTGGCAAGCCATGTTTTAGGATCTTGTTGGTACCTCCTGGCAATTGAAAGGCAAGAAGAATGCTGGA
ACAAAGTTTGTAGTCTTCAGCATCCAGAATGCCAATATTGGTATATTGATTGCCATAAAGTTAATGACCC
CGGTAGAGCTGCATGGTTCAAATCAAGCAATATTTCAGGTCTTTGTGGTGCAAGTAGTGAATTCTTTGAT
TTTGGCATTTATAGTGATGCCTTAAAATTTACTGTTATAGAACCAAGGTTCTTGAACAAGTACTTCTATT
GTCTTTGGTGGGGCCTCAGGAATTTAAGCTCTCTGGGACAGAATCTTTTAACAAGTACATATGTTGGAGA
AATAAATTTTGCTATTATCATTGCAGTTCTAGGATTGGTGCTTTTTGGACTGCTTATTGGGAATATGCAA
ACATACCTCCAATCCACAACCATGCGACTGGAAGAGTGGAGGATTAGGAGGACTGATACAGAAAAATGGA
TGTGTCATAGGCAGCTACCACATGAATTAAAACAGAGTGTGCGTAAGTATGAACAGTACAGATGGATTGC
AACGCGAGGAGTTGACGAGGAAGCTGTTCTGAAAGGTCTCCCCATGGATCTCAGGCGAGATATCAAGCGC
CATCTCTGTCTTGATCTAGTTAGACAAGTTCCACTCTTTAATCAAATGGATGAAAGGATGCTGGATGCAA
TATGTGAAAGGCTAAAACCATCTCTGTGCACTCCAAACACTTGCCTAGTTCGTGAAGGTGATCCTGTCAC
TGAGATGCTCTTCATAATCCGAGGCCATCTAGATTCTTGTACAACAAATGGTGGTCAGTCCGGCTTCTTC
AATTTATGCCACCTTGGCCCTGGTGACTTCTGTGGTGAGGAATTGCTGACATGGGCCTTGGACCCTCGGC
CAAGTGTTGTCCTGCCCTCATCCACGCGAACAGTGGGATCCATTAGTGAAGTTGAAGCTTTTGCTCTTAT
TGCAGAGGACTTGAAGTTTGTAGCTGCTCAATTCCGGAGGCTACATAGCAAGCAACTAAGGCACACATTT
AGATTTCACTCGCACCAATGGAGGACATGGGCTGCATGCTTCATACAAGCTGCTTGGTTCCGCTATAAAA
GACGGAAAGAGGCTTCTGAGCTCAAGAAAACACAGAGCTTTTTGGTATCTTCTAATGCTCCTGGAATAGA
GCAAACTAATACACCATTGCCTTCAATGGGATCAGGCTTTTCATACGTGGCAAAACTGGCAGCAAGTACT

AGAAGGGGTGGGAGCAAGCGATGTGGACACGAATTTGACATGCTGAGTTCTTTGCAAAAGCCAGTTGAAC
CTGATTTTACAGTTGAAGAGAGATGA
>ZjCNGC08
ATGGGTTATGATAATTCAAGATCTGTAAGATTTCAAGATGATCTGGAACTAGCAAAGCTCCCA
GCAGTCGATGGAGATGGTGTGGTTAAGCTCAAATACAAGATTGACGGGACACAGATACCAGAGCCAAGCA
GCAAGAAGGGTGAGAAGGAAGTGTTTGGAAAGACAACAACATCTTTAAAAGCTAAAGTACTGTCCAGAGT
CTTTTCTGAGGACTATGAGAGAGTGAAGAAGAAGATATTGGATCCTCGGGGACCAGCAATCCGGCGATGG
AGCAAGATTTTCTTAGTGACTTGTTTAGTTTCTTTGTTCGTGGACCCTTTGTTCTTTTACCTGCCAGTGG
TCCAGGATGAAGTGTGCATCGATATTGGAATACCTCTTGAGATCATTCTTACGATAATTAGATCACTAGC
TGATATTTTTTACGTGATTCAAATATTTATTAAATTTCGTACAGCTTATATTGCACCTTCCTCTCGTGTA
TTTGGGAGAGGAGAGCTTGTTATCGACTCTTCAAAGATTGCAAGGAGGTATTTTCGCCAGAGCTTTGTGA
TCGACCTCATTGCGGCCCTGCCTCTTCCTCAGGTGTTAATTTGGATGGTTATCCCCAATCTTAGTGGTTC
AACCATGACAAACACCAAAAACTTTCTTCGGTTCATCATAATCTTTCAGTACATACCAAGACTATTTCTG
ATATTCCCACTCTCATCGCAAATTATCAAGACCACTGGTGTTGTGACAGAGACAGCATGGGCTGGGGCTG
CATATAACTTGATGCTCTACATGTTGGCAAGCCATGTTTTAGGAGCTTGCTGGTACCTTCTATCAATTGA
GAGACAAGAAGCATGCTGGAGAAGTATTTGTCATTTAGAGAATTCTCTTTGTCAGTATAAATACTTTGAT
TGCCACTGGCTAAAAGGCCTTGATAGACAAACCTGGTTCAAGTTGAGCAATGTCACTGCTCAATGCAATC
CAGATGAAAGCTATTATGATTTTGGGATTTATGGTGATGCATTGACATTTGATGTGACAACCTCATCATT
CTTCAACAAATACTTCTACTGTCTTTGGTGGGGCTTAAGAAATTTGAGTTCCTTGGGACAAAATCTTTCC
ACCAGCACATACGTTGGAGAAATAACTTTTGCCATAATTATTGCCACCCTTGGATTGGTTCTCTTTGCGT
TGCTCATTGGTAATATGCAGACATATCTACAATCCACAACTGTTCGATTAGAAGAGTGGAGGATCAGAAG
AACTGATACAGAACAATGGATGCATCACAGGCAGCTACCGCCAGAATTAAAACAATCAGTGAGAAAATAT
GACCAATACAAATGGATTGCAACTAGAGGAGTCGATGAAGAAACTCTCCTCAAAGGCCTGCCTATGGATC
TCCGTCGAGACATCAAGCGCCATCTTTGCCTTGAACTAGTTCGAGGAGTACCTTTGTTTGATCAAATGGA
TGAAAGAATGCTGGATGCAATATGCGAGAGGCTCAAACCAGCATTGTGTACTGAAGGCACGTTTCTTGTC
CGTGAAGGGGACCCTGTAAATGAAATGCTATTCATAATCCGAGGCCACCTTGATTCCTTCACCACAAATG
GTGGCCGAACTGGGTTCTTCAACTCATGCAAAATCGGACCAGGTGACTTCTGCGGAGAAGAACTGCTGAC
ATGGGCCCTGGACCCACGTCCAAGTGTCATCCTCCCATCTTCAACTCGCACTGTCAAAGCTCTCTCTGAA
GTAGAAGCATTTGCTCTTGTTGCAGAGGACTTGAAGTTCGTAGCTTCACAATTTAGAAGACTCCATAGCA
AACAGCTTAGACACAAGTTCAGATTCTATTCTCATCAATGGAGATTATGGGCTGCATGCTTCATTCAGGC
GGCTTGGCGGCGGTATAAGAAGCGAAAGGAAGTGGCTGAGCTCAGAGCTAAAGAGAGCCCCACGGCTGC
TGAGCCTGAGCAACCAAAGCAGGAATCAGGCTTGGCTATGTATGCAGCTAGGCTTGCGGCTAGTACCAGAA
GGGGTGTGAATAATAAAAACCATTCAGGGTCGGATTCTGGGGTTGTCAGCTTGCAGAAGCCAACTGAACC
TGATTTTTCTGTTTGTGAGGAATGA
>ZjCNGC09
ATGAATAGAATAATCTCCAAACGGGCTGCTAAATTCCGCCCCTTCCGGCGACTTTCTTCTAAAATCAGAGACG
ACGCTGCCGCCGCCGCCACTGCCAACGAGGAAGAGTACCCACACCTCATTCTATGGCGCTACCAAATCCTTG
CTCCCGACAGCGATATTGTTGCCCATTGGAACCACATCTTTCTTGTCACCTGCATCGTCTCTCTCTTCATAGAC
CCTCTCTATTTCTTCCTTCCCTCCGTCGGAGGCCCGGCTTGCTTGTCCACCGACACCGGCCAAGCCATCACCA
TCACCTGCTTTCGCACCGTCACCGATCTCTTCTTCATCCTGCATATCGTCATGAAGTTTCGCACCGCCTTTGTC
GCACCAAGCTCTAGAGTTTTCGGTCGCGGCGAGTTGGTCATGGACCCCAGAGAGATTGCTCTGCGCTACTT
GAAGAAGGATTTCCTCATCGACCTCGCTGCTGCTCTTCCAATTCCCCAGATTGTTATCTGGTTTGTAATCCCG
GCAACAAGAAATTCCAGAGCTGACCATGCCAACAACACTCTTGCACTTTTCCTTCTATTTCAATATGTTCC
TAGACTGTTTCTCATCTTCCCTCTGAACCAACGAATCATTAAAACTACAGGGGTTGTTGCTAAGACTGCA

TGGGCAGGAGCTGCATACAATCTCCTTCTTTACTTGTTAGCTAGTCATATATTAGGATCTGCATGGTATC
TACTGTCCATAGGACGCCAATTCTCCTGTTGGCAAAATGAGTGTAGGAAGGAGCATGAATCAAAGCTTGT
ATCTTGTCTTTATAATTTTCTGGATTGCAAAAGTAAAGACCAGCCTGAACGCCAATATTGGCTTAATGTC
ACTAAAGTTATTAGTAAATGCGATGCGAGGGACAATGGTATCGAATTCCAGTTTGGAATGTTTGCAGATG
CATTTACTAATGACGTTGTCTCTTCACCATTCATTGACAAGTACCTTTATTGTCTTTGGTGGGGTTTGAG
AAACTTAAGTTCATATGCGACGAATGTGGATACAAGCCGGTATATTGGCGAGACAACATTTTGTATTTTC
ACATGCATAATCGGTTTAATTCTGTTCTCCCATTTGATTGGAAACATGCAGACTTACTTGCAATCAATGA
CGATAAGACTTGAAGAATGGAGGATTAAGCGAAGAGATACGGAGGAATGGATGAGGCATCGCCAGTTACC
TCCAGATCTGCAAGAACGCGTTCGACGTTTTGTGCAATATAAATGGCTTACCACCAGAGGGGTTGATGAA
GATTCAATACTGCGTTCGTTTCCTTTGGATCTTCGCCGTGAAATTCAAAGGCATTTATGTCTTGCTCTTG
TTCGCCGTGTCCCCTTCTTCTCACAAATGGATGGCCAGCTTCTCGATGCCATATGCGAACGTCTTGTATC
ATCTCTAAGCATCCAAGGCACCTACATCTTTCAAGAAGGTGATCCACTTGATGAGATGCTGTTTATTATT
AGAGGAAAACTCGAGAGTTCCACAACGGATGGAGGAAGATCCGGGTTCTTCAATTCCATCACTCTAGAAT
CTGGTCACTTTTGTGGGGAAGAGCTATTGACTTGGGCCTTAATGCCAAACCCTAGCATCAATCTTCCTAC
TTCCACTAGGACTGTTAGAGCTCTTACCGAAGTTGAAGCTTTCGCACTTCGTGCTGAGGATCTTAAGTTT
GTTGCAGGTCAGTTCAAACGTCTCCATAGCAAGAGACTTCAGCATGCTTTTAGGTACTATTCTCACCAAT
GGAGAACATGGGGTGCTTGTTACATACAAGTTGCTTGGAGAAGGTATATCAAGAGGAAGATGGCAAAGGA
TTTGAGTCAGCAGGAGAGCTTCTGCTATTTGAATATTCCCAGCCAAGAAAGCAACTATGCTGATGAGCTA
GAACATGGAAATTATAACTATGGGACTGGTGACAGTTTAGTAGAGGACAATGCAAGCCATTTCCAGCATC
TTGGGGCCACAGTTCTGGCTTCGAGGTTTGCTGCGAATACAAGAAGAGGAGTTCACCACAAAGCTGAAGG
GGGTAGCTCTGCTTCTACCAGCTTAAAGATGCCCAAGTTGTTTAAGCCAGAAGAACCCAATTTCTCAATA
GATGATGAAGATGAAGTTTAG
>ZjCNGC10
ATGGAGTTGAAGAAAGCAGAGAAGCTGGTCAGGTTTTACCATGATGGGAAAACAGATCCTCATTTTACTTG
GATGACAAACGAAACCCAGCAGCATCTGGAAAAGCCATTGCCGGTTTATAAAGTATCAGGGCCAATTTTGTT
GAAAACAGAAGGAGGTGGTGTTGTTGATGGGAACAGTATCAATATTGTCAAATTTGTGAAGTTTAAGGTTTT
CCAAGAAAATCATGAGCCATGGCAGCAGAGAATTCTTGATCCGGGAAGTGATATTTTCTTGCAATGGAACCG
GGTTTTTCTCATCTTTTGCTTAGTAGCCCTCTTCCTAGACCCATTGTTTTTCTACCTTCCATCGGTACAAAATTT
TGGTACCACATCGTGTTTGGAAACTGATTTGAATTTGAGAATTGTGGTGACTTGTTTTCGAACTCTTGCCGA
TGTTTTCTATTTGTTGCACATGGTCATAAAGTTTAGGACAGCCTATGTCTCTCCAAGTTCAAGAGTTTTT
GGGAAAGGTGAACTTGTTATGGATCCAAAATTGATTGCAAGGAGGTATTTGAGATCAGATTTCTTCATAG
ATCTCATAGCTGCACTGCCTCTTCCTCAGATTCTGATATGGTTTACTATGCCTGCAATCAGAAGCTCACA
TTCTGATCATGCCAACAATGCCCTTGTTCTTATTGTTCTGCTCCAATATGTGCCTAGATTATATCTCATA
TTTCCATTAAGTTCAGAGATTATCAAAGCCACTGGTGTTGTCACAAAGACTGCGTGGGCTGGGGCTGCAT
ATAATCTCCTACTTTACATGTTGGCCAGTCATGTCTTAGGAGCATCATGGTATTTGCTTTCGGTAGAACG
GTATGCAACATGCTGGAAATCTATATGCAGAAAGGAACTCAGCCCTGTGAGATGTTTTGCTAAGTACTTG
AATTGTGATACTTTAGATGATGGCGATCGCCAGAAATGGATAAACGGTACTTCTGTCTTTAGTAGCTGCA
CTCCGGGAGATTCTACCATTTTCAACTATGGCATATTTGAAAATGCAGTGACCAATAATGTCGTCTCATC
GGAGTTCATCGAGAAATATTTCTATTGCCTTTGGTGGGGCTTACAGAACTTGAGTTCGTATGGACAGAGT
TTGGCCACAAGCACGTTTATTGGGGAGACTTCTTTTGCCATACTCATTGCCATTTTGGGTCTGGTTTTGT
TTGCTCACTTGATAGGAAACATGCAGACTTATTTGCAATCCATCACTGTGAGACTTGAGGAATGGAGGCT
CAAGCGTCGAGACACCGAGGAGTGGATGCAACATCGTCAACTCCCTCAAAATCTTCGAGAACGTGTAAGG
CGTTTTGTTCAATATAAGTGGCTTGCAACTCGGGGAGTTGATGAAGAATCAATCTTACGTGGATTACCAA
CAGATCTTCGTCGAGACATCCAACGCCACCTATGCTTGGACCTTGTTAGACGTGTCCCCTTTTTTGCGCA

AATGGATGATCAATTACTTGATGCCATATGTGAGCGTTTGGTATCCTCCTTAAGTACTCAAGGAGCTTAC
ATAGTTCGTGAAGGTGATCCTGTAACAGAGATGCTTTTTATCATCCGAGGGAAGCTAGAGAGCTCCACAA
CAAATGGAGGTCGAACAGGCTTCTTCAACTCAATTACTTTGAGGCCTGGAGACTTCTGTGGAGAAGAGCT
ACTTGCATGGGCATTGGTTCCGAAATCTACACTCAACTTGCCTTCTTCCACAAGAACAGTAAGATCCCTT
GATGAAGTGGAAGCATTTGCACTCCGAGCAGAAGATCTCAAATTCGTTGCCAATCAGTTTAGGCGGCTCC
ATAGCAAGAAGCTGCAGCATACCTTCCGTTTCTACTCTTACCATTGGAGGACTTGGGCTGCCTGCTTCAT
TCAGGCTGCTTGGCGACGATACAAGAAGAGAACGATGGCAAAGATCCTTAGCATGACAGAGTCCTCTTAC
ACTCTTGATCCGCAAGTACCTGGTGAAATGGAACGAGAAGAGGAAGACGAAGATACGAATTCTTCACCTT
CCTCACAAGCAAAACAGAACCTCGGGGTCACAATATTAGCTTCAATGTTTGCAGCAAACACAAGAAGAGG
AGCTCAGAAGATAAAGGATGTTGAAATGCCCAAGTTGCAGAAGCCTGAAGATCCAGACTTTACAATGGAG
CCTGATGATGAATAG
>ZjCNGC11
ATGCACAGCTTCCCATCTTACCTTCCAGCGCCTTCGTCTCGCTTCCCCCATTTCTCAACCCCTTTCTCTCTCCGC
AAGAAAATCCCATGGTGGTACCAAATCCTCGACCCGGGATCCGATTTGGTCAGCCGCTGGAACCAGATCTTC
CTCATCACTTGTCTCATCGCCATGTTCATCGACCCCCTCTACTTCTACCTCCCTGTAATTTCCAAGTCCGATTCA
GCTTGTATGGACATCGACATCAACCTCGGCGTCCTCGTCACCTTGCTCCGTACCTTCACCGACTTCTTCTATC
TCCTTCACATTACCATGAAATTCAGGACCGCTTTTATCGCCCCGAGTTCCCGGGTTTTCGGCAGGGGTGA
GCTTGTCACTGACCCAAGAGCCATTGCCATTCACTACCTCAAATCTGATTTCTTCATTGATTTCGCAGCC
ACTCTTCCTCTCCCACAGATTGTTATTTGGTTTGTAATTCCCGCATTGAAGAATTCAACAGCCGCTCATG
CTAATCACACTCTGTCTCTAATTGTTCTTATTCAATATATTCCTCGATTTTTCCTTATTTTCCCTCTGAA
CAGAAGGATTATCAAAACAACTGGTGTAATAGCCAAGACTACTTGGGCAGGGGCAGCTTACAATCTCCTT
CTATATATGCTTGCTAGTCATGTAACTGGAGCTTCATGGTATGTGTTATCCATCCAGAGACAGTACCAGT
GCTGGAAAATGGAATGTCGGAAGGAGATGAATGGCACGCATTCTCCTTCCTGTCATGCTTCGTTTTTAGA
TTGTACCAATAAGGATAATCCAGAACGCGATCTTTGGCTGGGCCGTACCAATATTGTTGTTCATTGCGAT
GCTCTAAATGATGACAGAAATTTCGATTTTGGAATGTTTGCGGATGCTTTTACTAGTCAAATTGCTAAAT
CAAACTTCAAGGAGAAGTATTTTTATTGCCTTTGGTGGGGTTTGAAAAGTTTAAGTGCATATGGACAAAA
TATTATTGCAAGTACCAGGAGTGCTGAAACATTATTTAGCATTCTTATTTGCACGGCTGGTCTCATTCTG
TTTTCACATCTCATAGGCAACATGCAGAGCTATCTGCAATCTACAAGTGCTAGACTTGAAGAATGGAGGG
TCAAACGAAAGGATACAGAGGAGTGGATGAGGCACCGTCAACTCCCTCCGGAATTGCAAAATCGTGTTCG
TCGGTTTGTTCAGTATAAGTGGATTGCCACAAGAGGTGTAGATGAAGAAGCCATTTTGCAAGATTTGCCT
TTGGATATTCGGCGTCAAATTCAAAGGCATCTCTGTCTTGCTCTTGTTCGTCGTGTTAATTTCTTTGCAC
AAATGGACAATCAGCTCCTGGATGCGATATGTGAACGTCTTGTTTCATCCTTGAATACCAAGGACACATA
CATTGTTCGGGAGGGTGATCCAGTGAACGAGATGCTTTTCATCATTAGAGGCCAACTTGAAAGCTCCACC
ACTGATGGAGGAAGGTTAGGATTCTTCAATTGCATTACCCTTAGACCAGGTGACTTTTGTGGCGAAGAGT
TGCTGACATGGGCCTTGATGCCCACTTCAAATCTGAGCCTTCCCACCTCTACTCGAACTGTAAGGTCACT
TACTGAAGTTGAGGCATTTGCACTTCGAGCAGAAGACCTCAAGTTTGTTGCCAGTCAGTTTAAGCGCCTA
CATAGCAAAAGACTGCAGCATGCTTTTAGGTATTACTCCCACCAATGGAGAACTTGGGGAGCTTGCTTTA
TACAAGTTGCTTGGCGACGGTATAAAAAGAGAAAGATGACAATTGATTTGGCAAGACAAGAGGAGTTATA
TTACGGTAACAATTTGGATAATGAATCTCCATATGCAGATTACAATGAGAGTGGTGGTCATAGTTCATCA
AGGGATGATCACAGGGCACAGCATCTTGGAGCTACAGTTTTGGCTTCGAAATTTGCTGCAAACACCAGAA
AAGGAGTTGCCCATAAGGTTGCTTCTCTTGACCCTGATGCCACCTGCTTAAACATGCCCAAAAAGCTTTT
CAAACCAGAGGATCCTACATTTTTAGCAGACCCTGATGACAGTTGA
>ZjCNGC12
ATGGCTGCTTATTAAAAAGATGAGATGCCGATGTTGTCTAACAATCATAGTTGGTCATCAGATGATAATG

TAAATTCGCCATTTGAAAGATTTGGCCGGACACAAAGTGCATCATTTGCCATTTCCATGAACTCCACGGA
ATCTTATGCAAGTGAGCCTAAGCTTGTAGGGCATACTGGCCCCTTGAGAAGTCAAAGAACTTCATTCATA
CCTATGAGTGGTCCATTAAATGTCCATCAACCCAATAACCTTTTACGGCCGAGTCGTAGTGTAGCAGTGA
AAAAGGAAGCAGAATTGTTAATGGTTTTGGAGCTCTTGATGGCAATAGCTAAAGTATTGTCACAATGTAG
AGGAGTGGCTTCAACTTGCATTTCACCAAAATCCTCAAGTACAAACCGAAGCCATATTGCTTGTGCAGTT
GCTTCTGATGCACTAATATATTCTGCCCCGGCAGTGGAGAGTGCAACACAGCTTTGTTTGACTGAAACCC
ATGAAAACACTCCACTTCCAAAACTGAAAGCATATCCAGATTTCAACTCTTTGCAGTTCCATAATGCTTT
CTATGGCGATGCGAAAGGTTGGGCAAGAAGAATTTTCTCTTTTATGAGTCCATATATTCCTGGAGTTATG
AATCCCCATGCTAAAGTTATACAAAAATGGAACAAATTTTTTGTCATAGCTTGTTTGGTGGCAATTTTTA
TGGATCCATTGTTTTTCTTCTTGTTATCTGTTCGACAGGACCATAAATGCATAGTTATTAATTGGCCCAT
GACAACAGTGATCGTATTTTTTAGAAGCTTATATGATATTATATACTTACTTCACATACTTCTTCAGTTT
AAGCTGGCTTATGTGGCGCCAGAGTCTAGAGTTGTTGGTGCTGGAGAGTTAGTTGACCATCCAAAAAAAA
TTGCTCGTAATTACCTTCAGGGATATTTTTTACTTGACTTCTTTAATGTATTACCACTTCCTCAGATTGT
GCTATTATTAATTGTACGAAAATCTTTAAGCTTATCTGGTGTAGACTATGCGAAAAATCTATTACGAGCT
GCAATTCTTGTTCAGTATGGTCCGAGATTGTACAGGTTTCTACCTCTGCTCGCTGGTCAGTCTCCTAGCG
GCTTCACATTTGAGTCCGCATGGGCTAATTTTAATATAAATCTTCTCATTTTTGTGTTGTCCAGCCATGT
GGTTGGCTCATTTTGGTACCTCTTTGGGTTACAGAGAGTTAATCAATGTCTTCGCAATGCCTGTCATCAC
TCTGATATCAACTACTGCATGAAATTCATAGATTGTGGATATGGGGATAACGTTGACAGCTTTAATAAGT
CATATCCAATAGAGTGGCCTAATTGGACAGACAATATAAATGCTAATTCTTGCTTCAGCACCGATGGCTT
TCCTTATGGAATCTACATTCAAGCTGTTAACCTTACCACGGAAAGAGGTGTCATCACCAGATATGTGTAT
TCACTCTTTTGGGGTTTTCAGCAAGTCAGTACTCTGGCTGGAAATCAAACTCCTAGCTACTTTGTTTGGG
AAGTCCTTTTTACTCGACTTGGCCTCTTGCTTTTTGCTCTTCTCATCGGAAATATGCAGAACTTTCTCCA
GGCTCTTGGAAAGAGGAGGCTAGAAATGTCTCTAAGGTGCCGTGATGTTGAACAATGGATGAGTAATCGC
CGCTTGCCTGAAGAGCTTACGAGGGATATCAAACAGCACCGTTTAAACTTTTCTGGCATGACTATTGATT
TAATGGTTTTAGAGTCTTTTACTTCGCATTAACTCATGGAGCATGTTCTGAAGAAAAAGAGATTTCGGAT
GATACCGTAGGTTCGGATATTTTCCCTTATGGATGAGCATATTTTGGATGCTATTTGTGAAAGACTAAGA
CAAAAGATGTACATCGGAGGAAGCAAAATTTTGTATCGTGGTTGTCTAATTGAGAAGATGATTTTTATTG
TTCGTGGAAAAATGGAGAGCATAGGAGAAGATGGGATAGGAGTTCCGTTATCTGAAGGAGATGTGTGCGG
TGAGGAGCTCTTCACATGGTGTCTTGAGCATTCTTCAGTAAACAAAGATAGAAAAAAAGTAAGGCTTCCT
GGACAGAGATTGTTGAGCACCAGGTTGGTGCAGTGCTTAACAAATGTAGAAGTATTTTCCCTCAGAGCTG
CTGATATTGAAGAAGTCACCAGCCTTTTCTCGGGATTCTTGCGCAACCCACGTGTTCAAGGATCTATAAG
GTATGAATCACCATACTGGAGAGGCCTGGCAGCAATACACATTCAAGTTACATGGAGATATAGAAAAAAA
CGCTTAAGTCGTGCTAAAATTTCTCAATCCGACCATTCATCAACTCAGTGA
>ZjCNGC13
ATGGCCACTGAGCATGAAATTTCACAAACCACTTCGCACATGCACTACAATCTCTCAGATTGCAGTGACA
GTGAAGAAGTAGAAGAAGAAGAAGCAAATCAAGAAGAAGAAGACGAAGATGATAACGGAAGCGAAATAA
GCTCCGTCGCCGGCGAAGGGGGTTTTTGCAACAGCTTGGCTTACGTGTGCGGCGGAGTAGGTCGACGACG
GAGGAAGCCGAAGGGGTGGTCGCTGGGTCAGGTACTGGACCCGAGAGCCAAATGGGTACAAGAATGGAA
CAGGGTGTTTCTATTGGTATGCGCGACAGGTCTGTTCGTGGACCCGCTCTTCTTCTACGCCCTATCGATAAG
TGACACGTGTATGTGTCTGTTCGTTGACGGTTGGTTCGCAATCACCGTGACGGTGCTCCGGTGCATGACC
GACGCGTTGCACGTGTGGAACATGTGGCTGCAGCTGAAGATGGCCAAGCGGTCGTTCTCGTTGGTTGGCG
TGGAAGGCAGGAACGGCCTCGGAGATACCACGGCTCGCTCTGTTGCTCTGCGGTATTTGAAAGCCAAGAA
GGGCTTCTTGTTTGATCTTTTTGTGATTCTTCCTCTGCCTCAGATAGTATTATGGGTAGCAATTCCTTCA
TTGCTAGAAAGAGGATCAATAACGAACGTGATGACGGTGTTCTTGATAATATTTCTGTTTCAATATCTGC

CGAAGATCTACCACTCAGTTTGTCTATTGCGTCGCATGCAAAACCTCTCCGGTTACATTTTTGGAACCGT
TTGGTGGGGAATTGCCCTCAACATGATTGCTTATTTTGTCGCATCCCATGCAGCAGGAGCATGTTGGTAT
CTACTAGGAATTCAAAGGGCAGCGAAATGCCTGAAAGAGCAATGCAGGGCTACAAGTGGCTGTGGTATGA
GAATACTATCTTGTAAAGAACCTATATATTATGGGACCACAAGCATGGTGAAAGATAGAGCAAGGCTTGC
TTGGGCTGTAAACAAACAAGCAAGGTCTACTTGCTTAGAGAATTCTGATAATTACGATTACGGTGCTTAT
AAATGGACTGTTCAGCTTGTCACCAATGATAGCCGCTTGGAAAAAATACTTTTCCCCATCTTTTGGGGCT
TAATGACTCTCAGCACATTTGGTAACTTGGAGAGCACAACAGAATGGTTAGAAGTTGTTTTCAATATCAT
TGTTCTAACCAGTGGACTCCTCTTGGTTACCATGTTGATTGGAAACATCAAGGTGTTTTTGCATGCAACG
ACGTCGAAGAAACAAGCAATGCAATTGAAAATGAGAAACATAGAATGGTGGATGAGGAAGAGACACTTGC
CTCTGGGTTTTAGACAGCGTGTGAGGAACTACGAGCGCCAACGCTGGGCTGCCATGCGTGGTGTTGATGA
ATGTGAGATGATTAGAAACCTCCCTGAGGGCCTTAGAAGGGACATCAAATACCATCTCTGCTTGGACTTG
GTTAGACAGGTACCTTTATTCCAACACATGGACGATCTGGTACTCGAGAACATCTGCGACCGTGTGAAGT
CTCTAATATTCACAAAGGGCGAAACAATTACAAGAGAAGGAGACCCAGTTCACAGAATGCTATTCGTAGT
GAGAGGTCACCTTCAGAGCAGCCAACTGCTAAGAGACGGTATCAAAAGTTGCTGCATGTTAGGCCCTGGA
AACTTCGTAGGAGACGAGCTCCTTTCATGGTGTTTGAGGAGGCCATTCATAGAAAGACTACCTCCTTCTT
CTTCAACCTTAATCACTCTCGAAACCACCGAGGCATTCAGCCTCGAAGCCGAGGATGTCAAATACGTGAC
GCAGCATTTTCGTTACACTTTCGTGAATGAAAAGGTTAAGAGGAGCGCAAGGTACTATTCTCCCGGTTGG
AGAACTTGGGCTGCTGTGGCTATCCAGTTGGCTTGGAGGAGGTACAAGCACAGGTTGACGCTCACTTCGC
TGTCGTTTATAAGGCCCAGGAGACCTCTCTCGAGATGTTCTTCATTGGGAGAAGATAGACTCAGGCTTTA
CACGGCTTTGTTAACTTCACCAAAGCCAAATCAAGATGATTTTGATTTTTGA
>ZjCNGC14
ATGTTCTCCATTTCAAGGTGGACTGAGTTATTCCAGCGCAAGAGCTTTCAAAACAGGAACAGTAGTG
TCAGTGGAAGCAGGAACGACAACGACATCGTCGTCGTTGTTCCCAAGGAATGCTACCAATGCACCCAAGC
TGGTATCCCTTTCTTCCATTCTACTCTCTGCCCCGACCCTTCCCACCACCCCCACTGGGAGGCCTCCGCC
GGGTCATCGTTCAGACCCGCCCCGACAACCACCGTTCCGACCAGAACCCATTCCCAAGCCCAAGATCACC
ATCATCATCATCATGATCCGTTGTTTCCTTTGGGGCGAGTGGTCGACCCGCGTAGCAAGACAGTGAAGAG
GTGGAACAGGGTGGTGTTGCTGGCACGTGGAATGGCGCTAGCCGTTGATCCGCTGTTCTTTTACGTGGTG
TGGATGAGTGGGGATGGGTCCCCTTGTTTCTACATGGACGTCGCGCTGGCGATGATCGTGACCGTCGTTC
GCACGTGCCTTGACGCGATGCATTTGGGGTACGTGTGGCTTCAGTTTAGGATGGCGTACGTGTCGAATGA
ATCCATGGTGGTGGGGTGTGGGAAGCTGGTGTGGGATGCTCGTGCCATTGCGTCTCGTTACCTTCGGTCT
CTCAGGGGTTTCTGGCTCGATGCCTTCGTCATCCTCCCTATTCCTCAGATAGTAATTTGGATTGTTGTAC
CAAAGCTGCTTAGAGAAGAACAAATCAAATGGATAACGAGAACATTTCTACTGAGCTTCTTATTTCAATT
CCTTCCCAAAATCTACCACAGCATTTACTTGATGAAAAAGCTACAAAAGGTTACAGGTTACTTGTTCGGC
AGCATTTGGTGGCGTTTTAATCTCAATGCCATTGCCTATTTAATTGCTTCCCACGTTGCTGGAGCATGCT
GGTACGTTCTTGCAACAGAGCGTTTAGTGTCATGCCTCCAGCAACAATGTGCAATAAGTAGAAAGTGTAA
TCTCAATCTTTATTGCAATAATTCACAAGCATTTGGAAATACTGACGGGGTTAATTATTCATCCATCAAT
TCCAAGTGCTTGGATGTAGATGGACCTTTCAACTACGGAATTTACAACCCGGTTCTTCTTGTCTTCTCCA
GCAATTCCCTCGCTGTTAGGATCCTTTATCCTGTGTTTTGGGGTTTGTTGAATCTAAGCTCTTTCGGTAA
TGAGCTAGAACCTACATGTAATTGGCTGGAGTTGATATTCAGTTGCTGCATAACACTTGCTGGTTTAATA
CTCTTTGTTACATTGATAGGGAATATACAGGTTTTCTTGCATACTGTGATGGCAAATAAGAAGAAAATGC
AGCTTAAATACAGAGACATGGAATGGTGGATGAAGAGGAGACAATTGCCAAACCCTTTAAGAGACAGAGT
CCGCAATTTTCAACGCCATAGTTGGTCAGCCATGGAAGGACAAGATGAGATGGAACTAATTCAACACTTG
CCCGATGGACTTCGAAGGGATATTAAACGTTTTCTTTGCATAGACCTTGTCAAGAAGGTACCTCTGTTCC
ACATGCTAGATGATCTTATTCTTGACAACATTTGTGATCGAGTCAAGCCTCTAATCTACTCTGCTGGTGA

AAAGATTATAAGAGAAGGAGACCCTGTTCAGAGAATGGTGTTCATAGTCCGTGGACGTGTAAACCGCAGG
CAAGGTCTAAGCAAAGGGTTCATAGCAAACAGTGTGGTTGAACCAGGTGGGTTCTTTGGGGACGAGCTAC
TTTCTTGGTGCCTACGCATTCCGGCTGCGAATTGGCTGCCTGTTTCTTTAGCCACATACACTTGCATTGA
CTCTGCAGAAGGATACGCCATAGACGCACACCAGCTTCGCTATGTTGTCGACCATTTTCGTTATAAGTTC
GCTAGCGAAAGTCTTAAGAAGACGGCCAGATACTATTCGTGTAACTGGCGAACATGGGGTGCAGTGGTCA
TTCAACTTGCTTGGCGCCGCCGTAGATTGAGGAATGGGACTAATCTTCCTATTTTGCAGAATGGAGACCC
TGAGGATCGGCTCAGGCAATATGCGGCCTTCTTCCTATCAATTAGGCCTCATGACCACCTTGAATAA
>ZjCNGC15
ATGCCCTCCCATCCCAACCCATTCCACTTCTCCATCCAAAGGTGGATTAACAATGGACGACAGAAACAAACTA
ACCAGAACACCTCACAGAGCGACAGCGACGACAACGACGACGACAGCCCAATCTCCAGCTCCGTCGAATG
CTACGCCTGCACACGTATGGGAGCTCCGGTGTTCCACTCCACCACGTGCGACAAGGCCAACCAGCCGCAGT
GGGAAGCCTGCGCTGGAACTTCCCTGATCCCAATCCACTCCCGAACCGACCTCAAGAAAGGCCTGGGCTCG
GCAGCCCGTCTCCAGCGGCCCACCGGCCCGTTCGGTCGGGTACTCGACCCCCGCAGCAAGCGCGTGCAGA
AGTGGAACCGCACGTTCCTTTTGGCGCGTGGGATGGCCTTGGCGGTCGACCCGCTTTTCTTCTACGCGCTG
TCGATTGGCAGAGGCGGTACTCCTTGCCTCTACATGGACGGCGGGTTGGCGGCCATCGTGACGGTGCTGCG
CACGTGCGTCGACGCCGTTCATCTCTGCCACCTGTGGTTGCAGTTTCGGCTGGCGTACGTCTCGAGGGAATC
TCTGGTGGTCGGTTGCGGGAAGCTGGTATGGGACACACGTGCCATCGCCACTCACTACCTTAGGTCTTTTAA
AGGCTTCTGGTTCGACGCTTTCGTCATTCTTCCAGTTCCTCAGGCTGTATTTTGGTTAGTTTTGCCAAAGTTA
ATAAGAGAAGAGCGGATTAAGCTGATAATGACAATACTTCTATTGATTTTTTTATTTCAATTCCTCCCAAAAGT
TTATCACAGCATATGCTTAATGAGAAGAATGCAAAAGGTCACTGGATACATATTTGGCACCATTTGGTGGGG
TTTTGGTCTCAATCTTATTGCCTACTTCATCGCTTCTCATGTCGCTGGAGGCTGCTGGTATGTCCTGGCA
ATACAACGAGTGGCTTCGTGCCTACGGCAACATTGTGAGAGAAGCACCAAGTGCAATCTCTCTTTGTCAT
GCTCAGAGGAAGTATGTTACCAGTTTTTATTACGTTCTGATACAGTAGGCAATCCTTGCGGTGGTAACTC
AACCGTGGCTAGAAGGCCGCTTTGCTTGGATGTCAATGGAACCTTCAATTATGGGATCTACCAGTGGGCT
CTTCCTGTCATATCTAGCAATTCAGTTTCTGTTAAGATTCTTTATCCTATCTTTTGGGGTCTAATGACTC
TCAGCACATTTGGCAATGATCTCGAACCTACAAGTCACTGGCTGGAAGTGATTTTCAGTATATGCATCGT
GCTAAGCGGCTTGCTTCTCTTTACACTATTAATTGGAAATATACAGGTATTCCTGCATGCAGTGATGGCA
AAGAAGAGGAAAATGCAGCTGAGATGTAGAGACATGGAATGGTGGATGAGAAGGAGACAATTACCGTCTA
GGCTGAGACAGAGAGTGCGCCATTTCGAAAGGCAGAGATGGGCAGCCATGGGAGGAGAAGACGAGTTG
GAAATGCTCAAAGATTTGCCTGAAGGTCTCCGGAGGGACATCAAACGCTACCTGTGCCTAGACCTCATTAAA
AAGGTTCCTCTGTTCCACAACTTGGACGATCTTATTCTTGACAATATATGCGACCGGGTCAGACCACTAG
TCTTCTCAAAAGATGAAAAGATAATAAGAGAAGGAGACCCTGTGCCTAGAATGATATTCATAGTTCGAGG
AAAGATTAAACGCAGCCAAAATCTAAGCAAAGGCATGGTGGCAACGAGTGTGCTTGAACCAGGAGGTTTT
CTAGGCGACGAGCTGCTTTCGTGGTGTCTTAGAAGACCATTTAACGACCGGCTTCCTTCATCATCTGCCA
CGTTCCTCTGCGTGGAATCCACGGATGCATTCGGGCTAAATTCGGAAGATCTGAGATACATAACGGATCA
TTTCAGGTACAAATTTGCGAATGAGAGGTTGAAAAGGACGGCGAGATATTATTCATCGAATTGGAGAACA
TGGGCAGCTGTAAATATACAACTTGCATGGCGTCGTTACAGGTTGAGGACCAGAGGTCCTGTGATTCCTG
CTGTTTCCACTGATAATGGAGGCACTGATCGGAAGCTAATGCAATATGCTGCTTTGTTCATGTCCATTAA
GCCACACGATCACCTCGAATAATCTTTAGTTTTTATTGAGTCCCAACCAAGTCGTATCCCATCCAATTTTCAATA
AAATCTTTGTTTTCCGGTCT
