# Supplementary material for: Genome-wide identification of CNGC genes in Chinese jujube (Ziziphus jujuba Mill.) and ZjCNGC2 mediated signalling cascades in response to cold stress
Source: BMC Genomics. 2020 Mar 2;21:191. doi: 10.1186/s12864-020-6601-5 (PMC7053155; doi:10.1186/s12864-020-6601-5)

## Slide 1
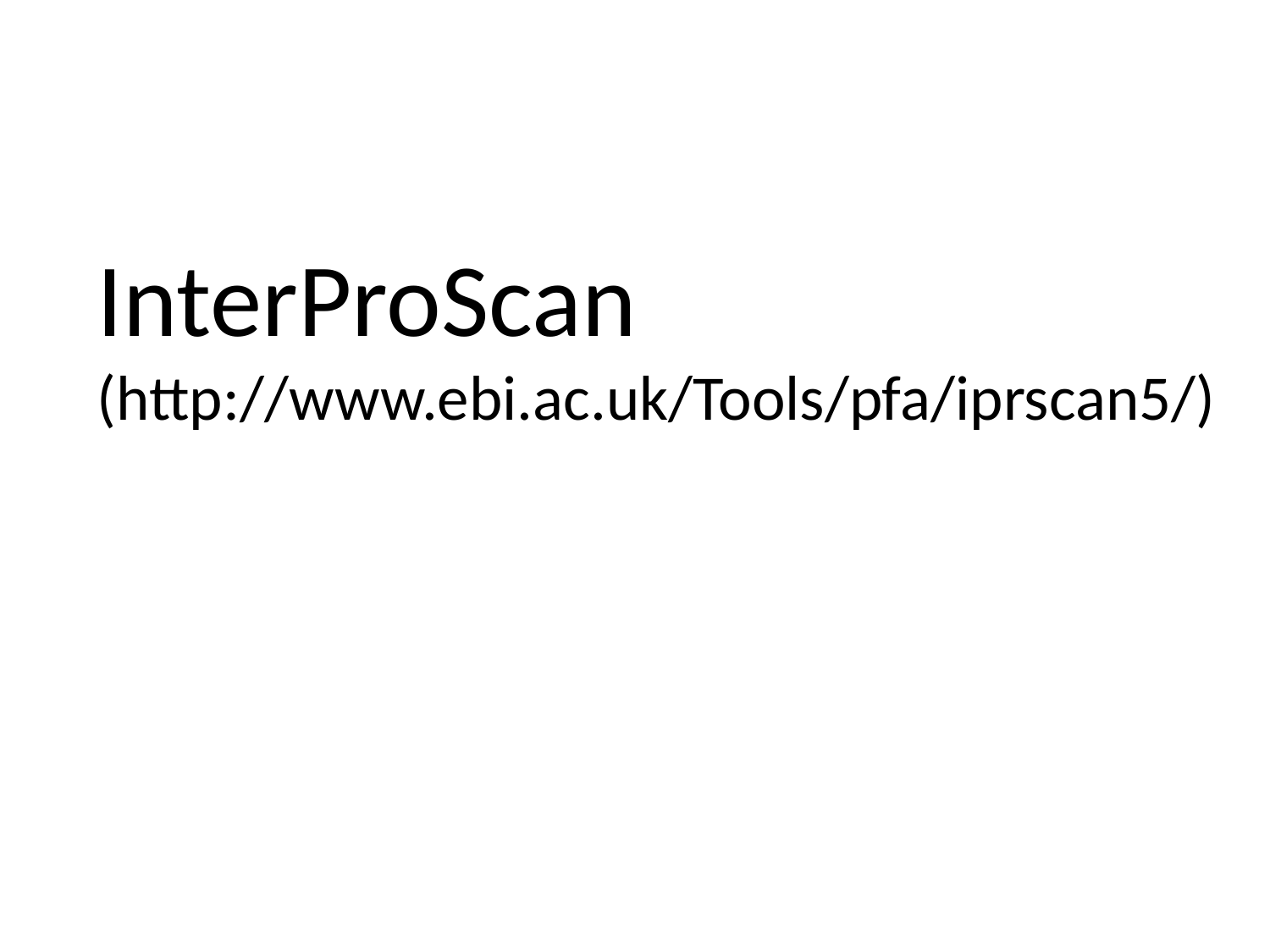

InterProScan
(http://www.ebi.ac.uk/Tools/pfa/iprscan5/)

## Slide 2
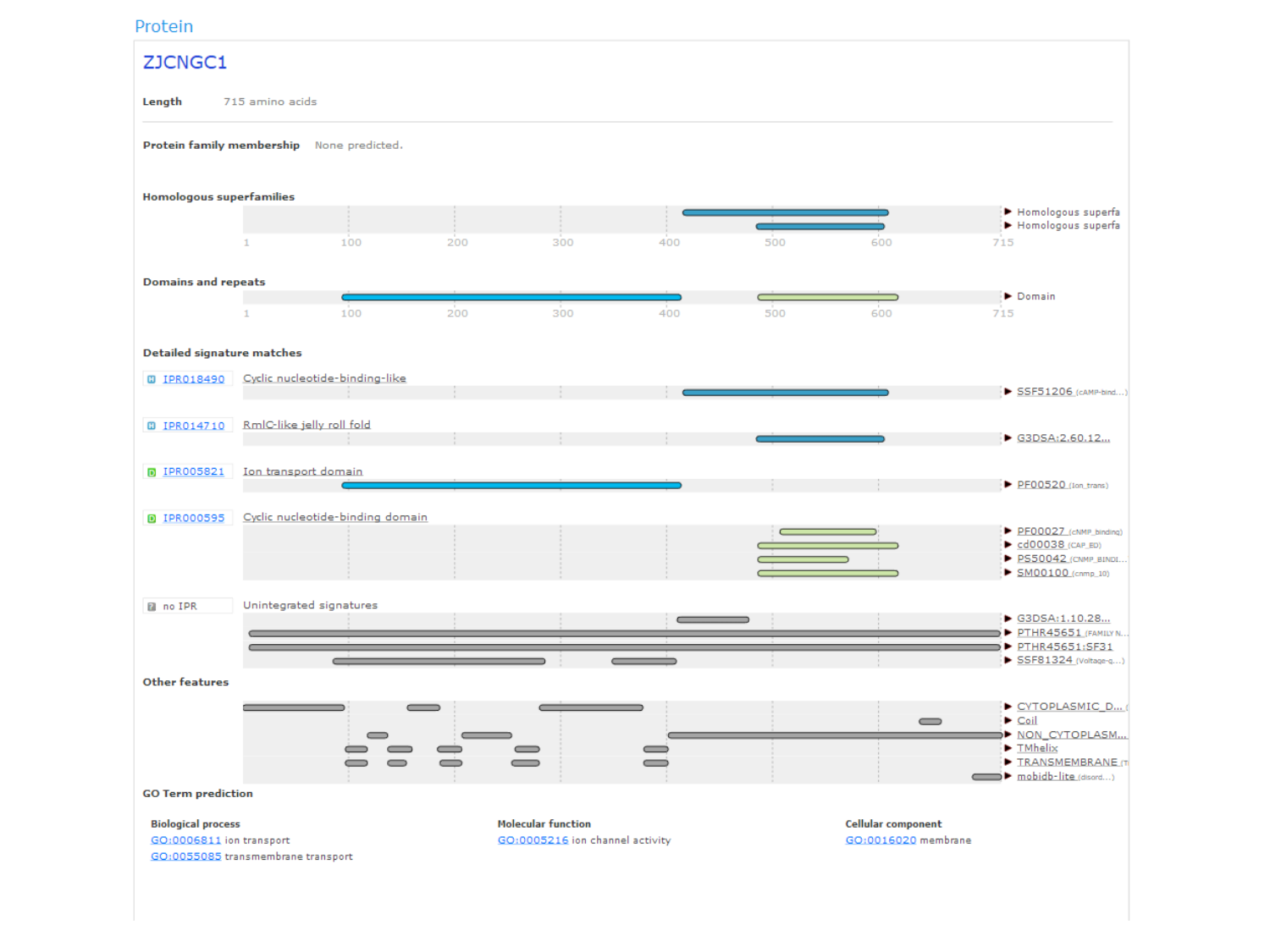

## Slide 3
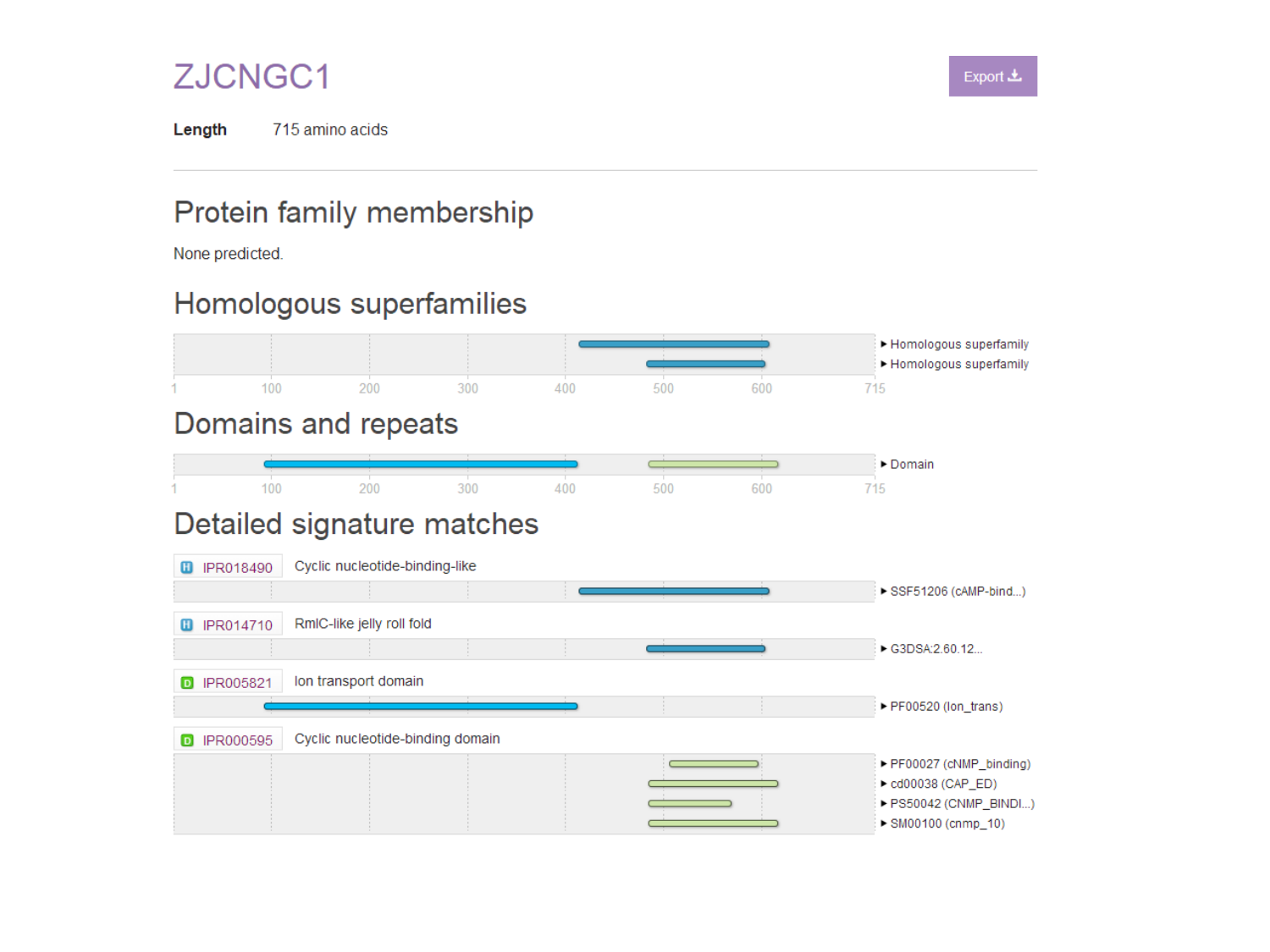

## Slide 4
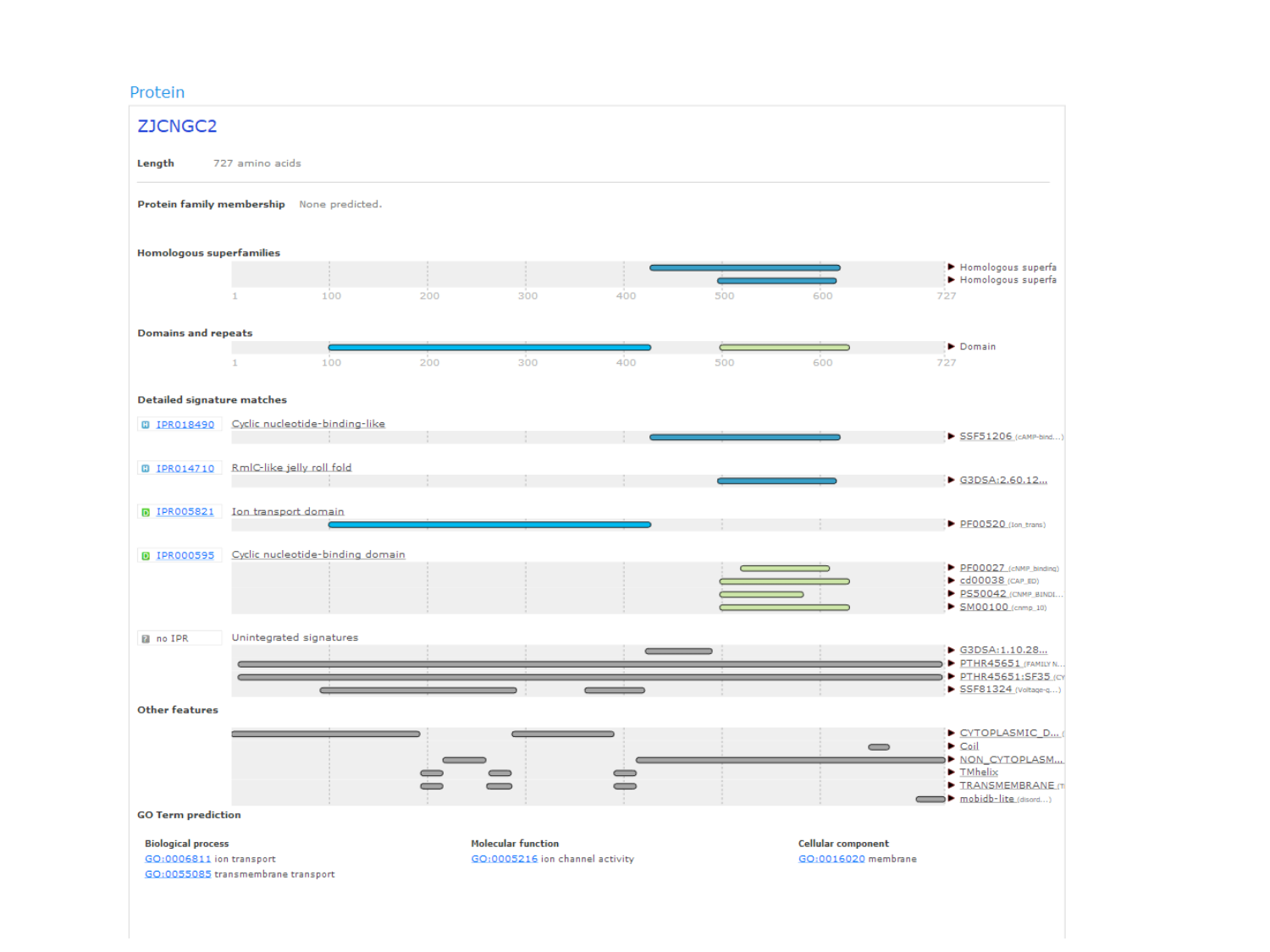

## Slide 5
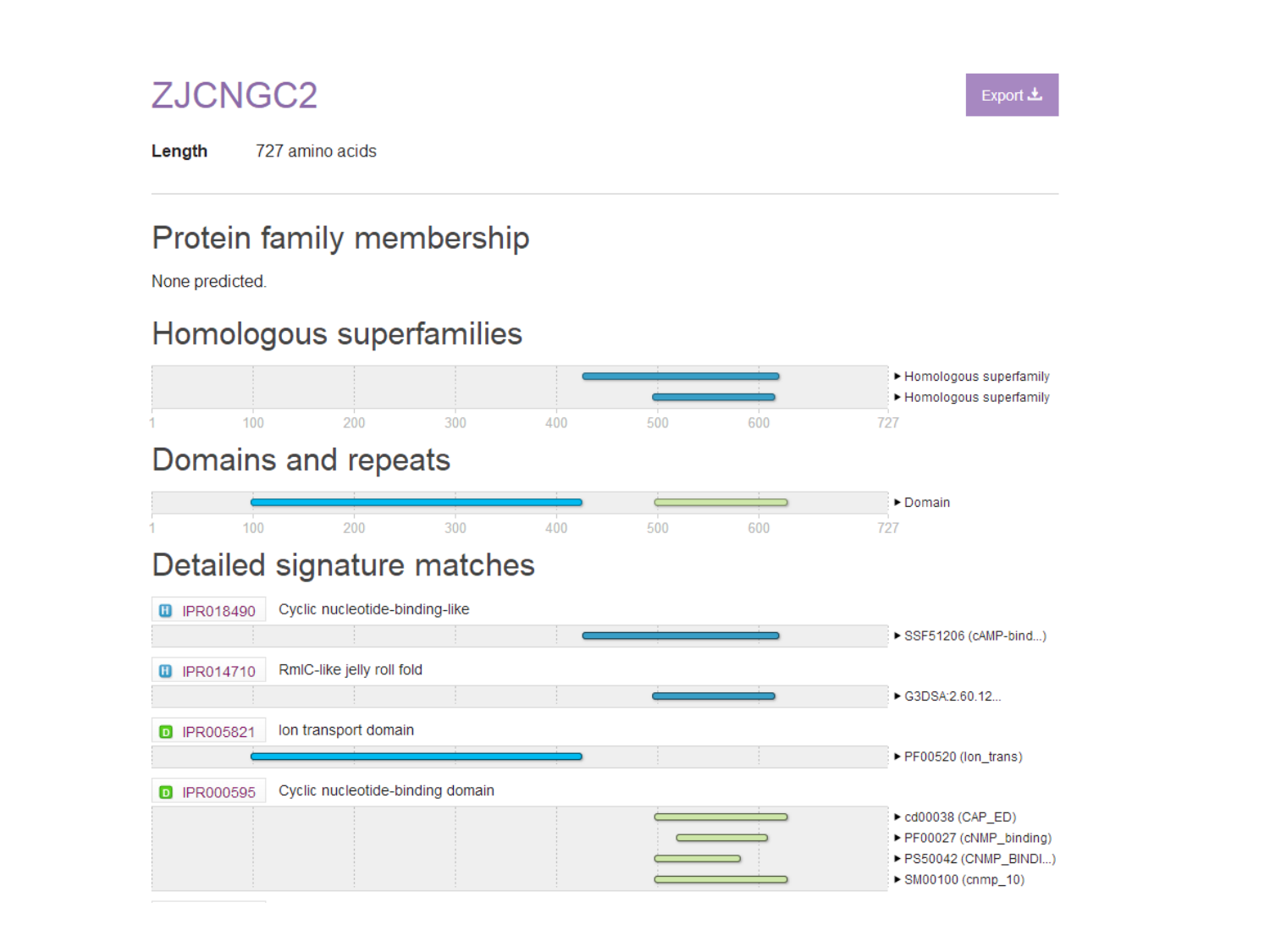

## Slide 6
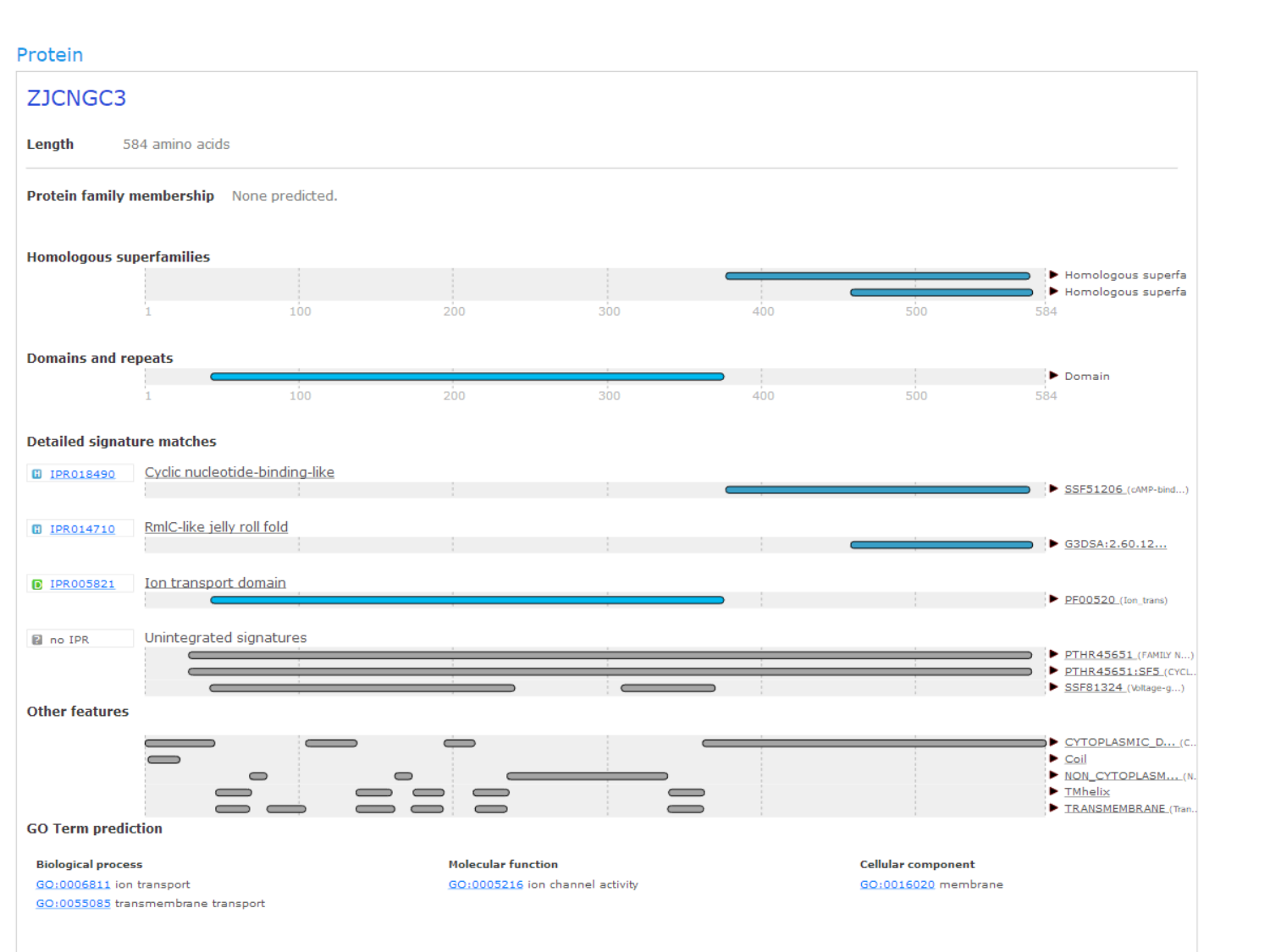

## Slide 7
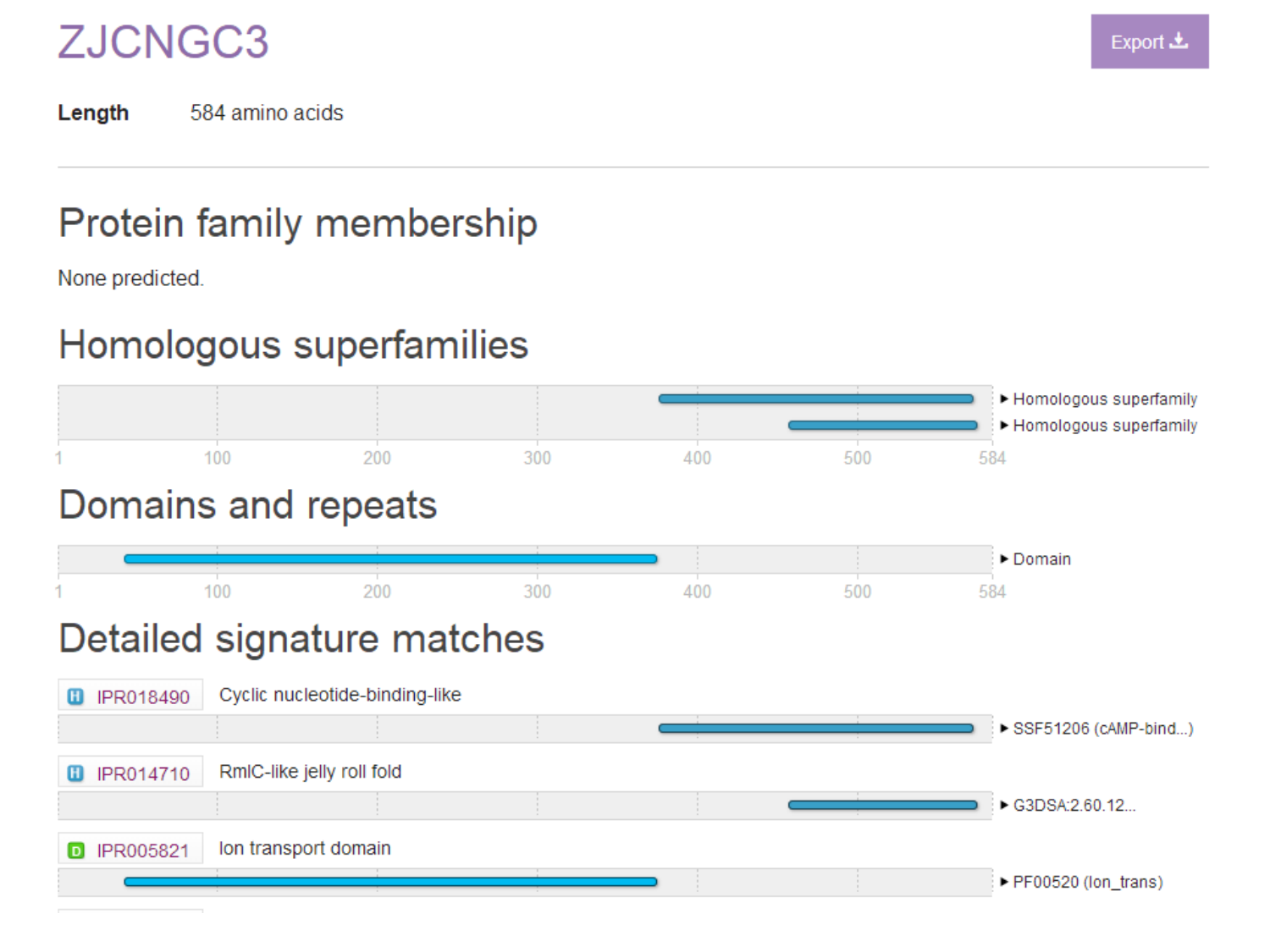

## Slide 8
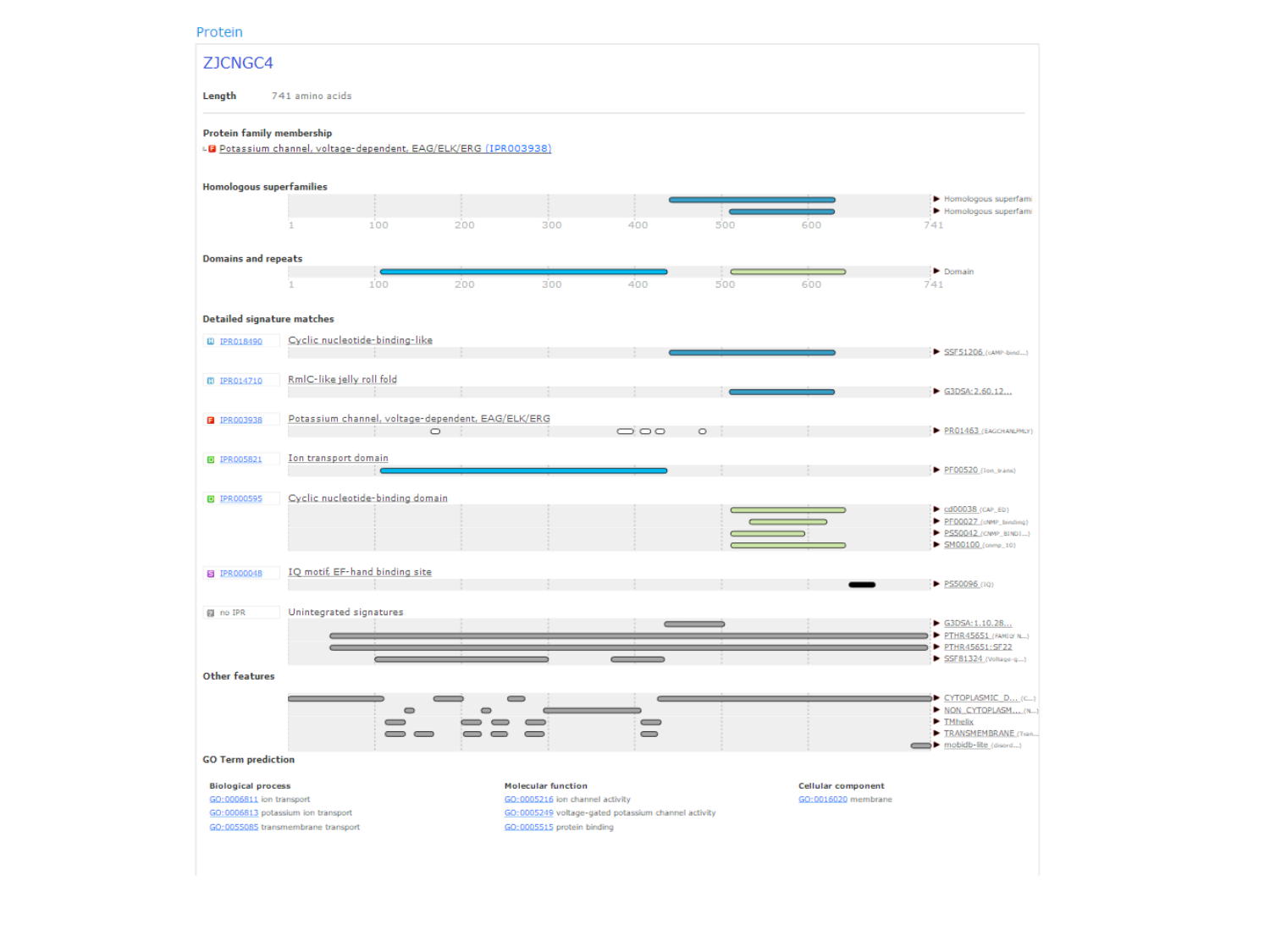

## Slide 9
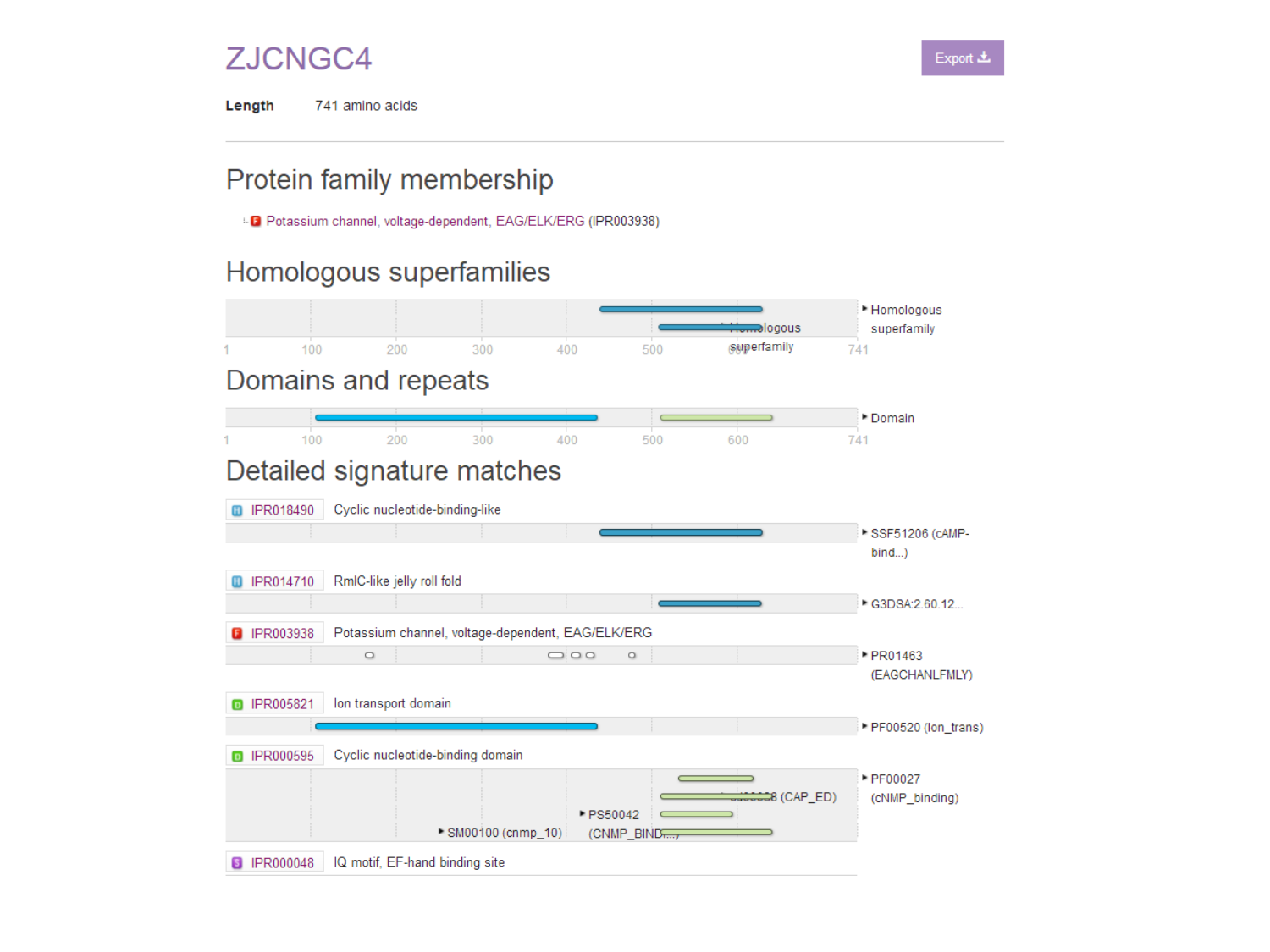

## Slide 10
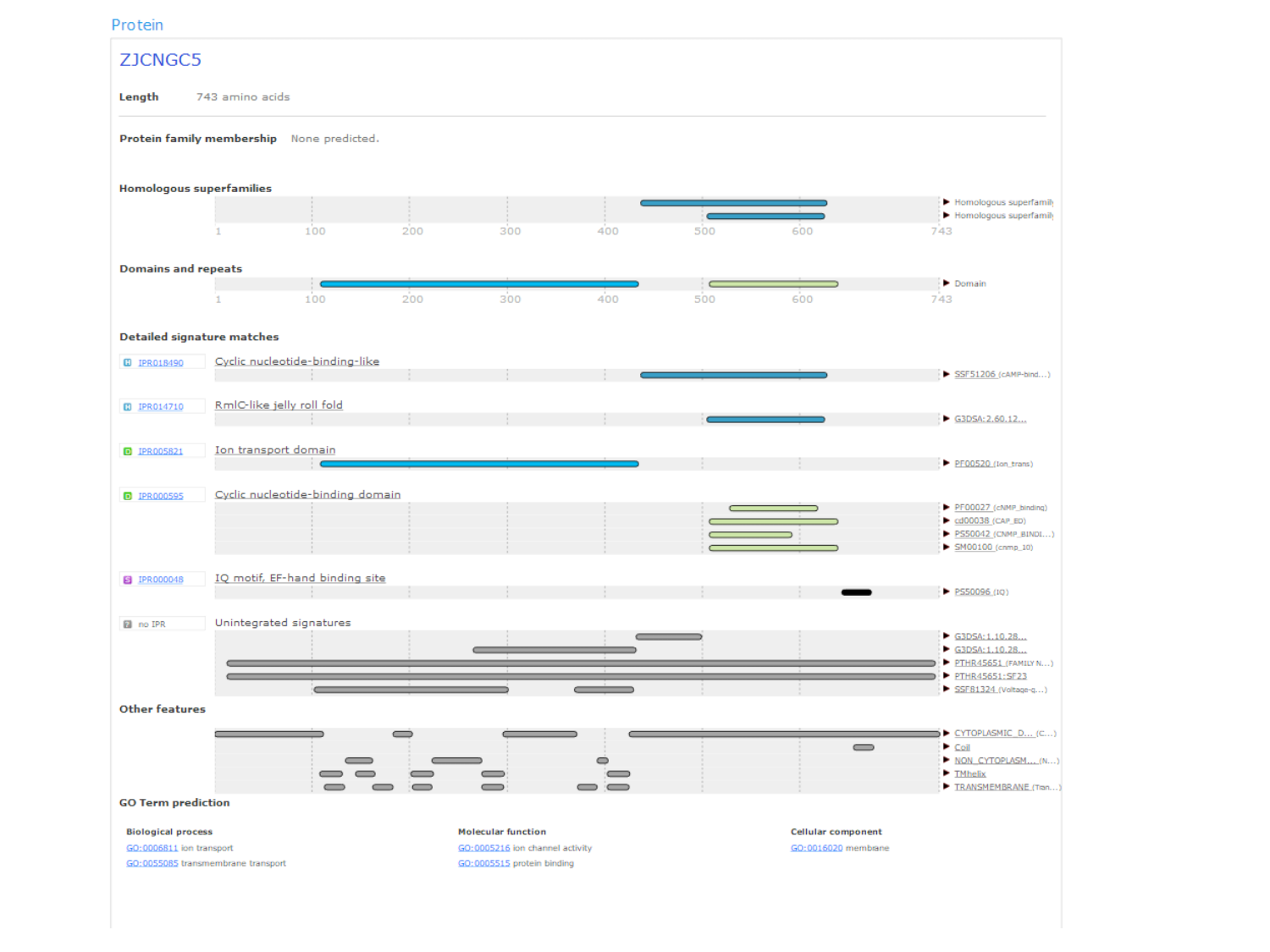

## Slide 11
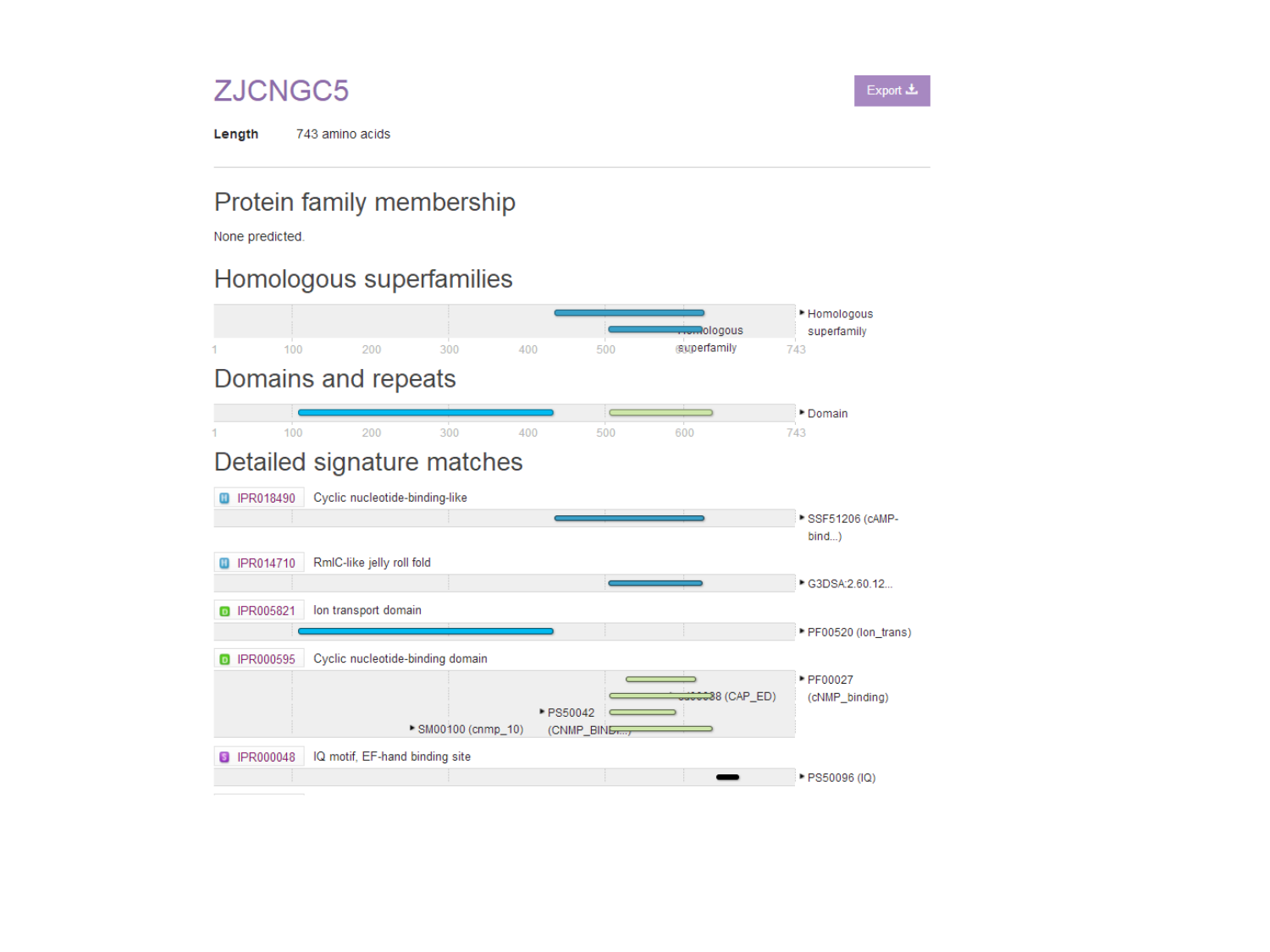

## Slide 12
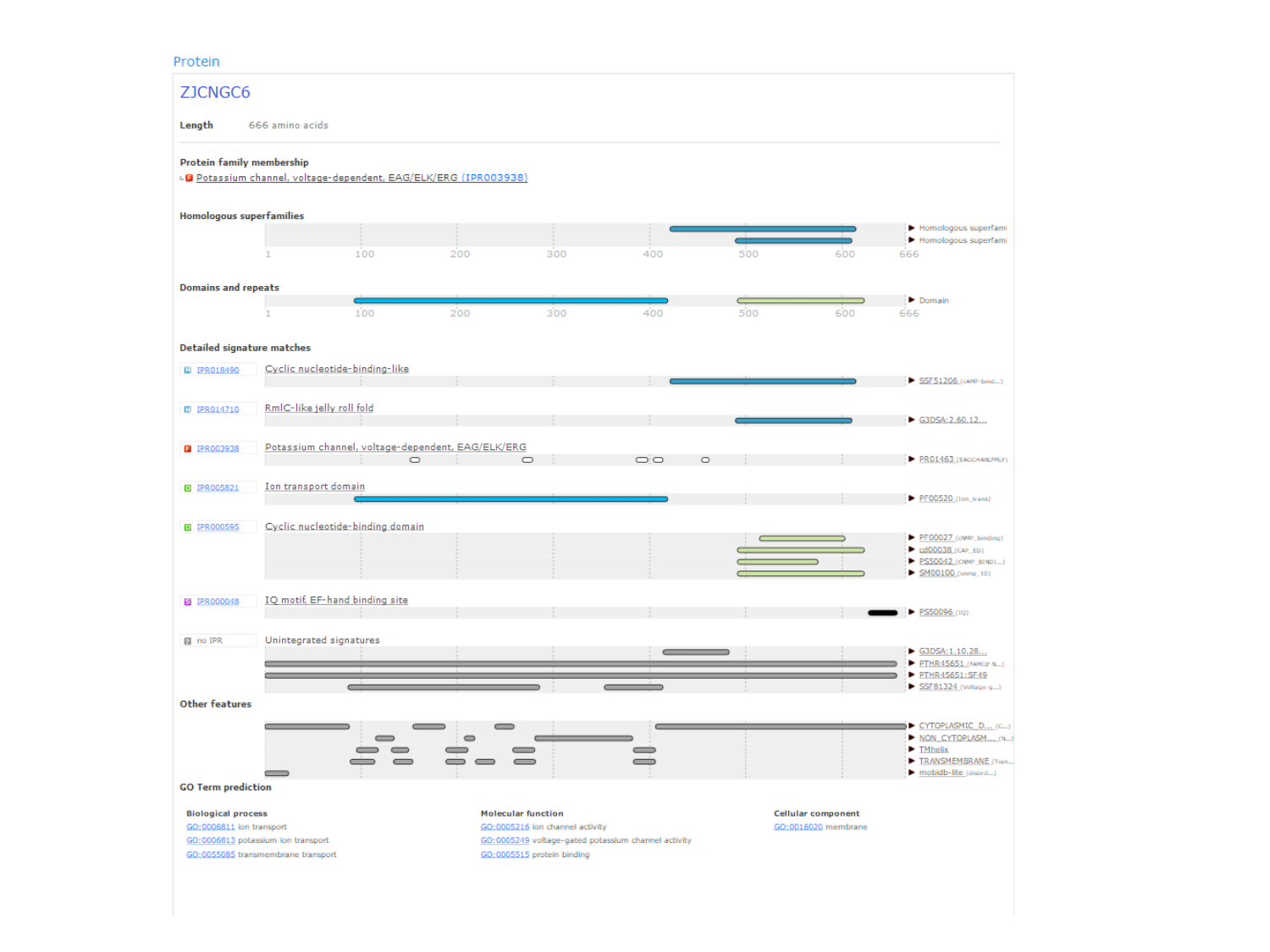

## Slide 13
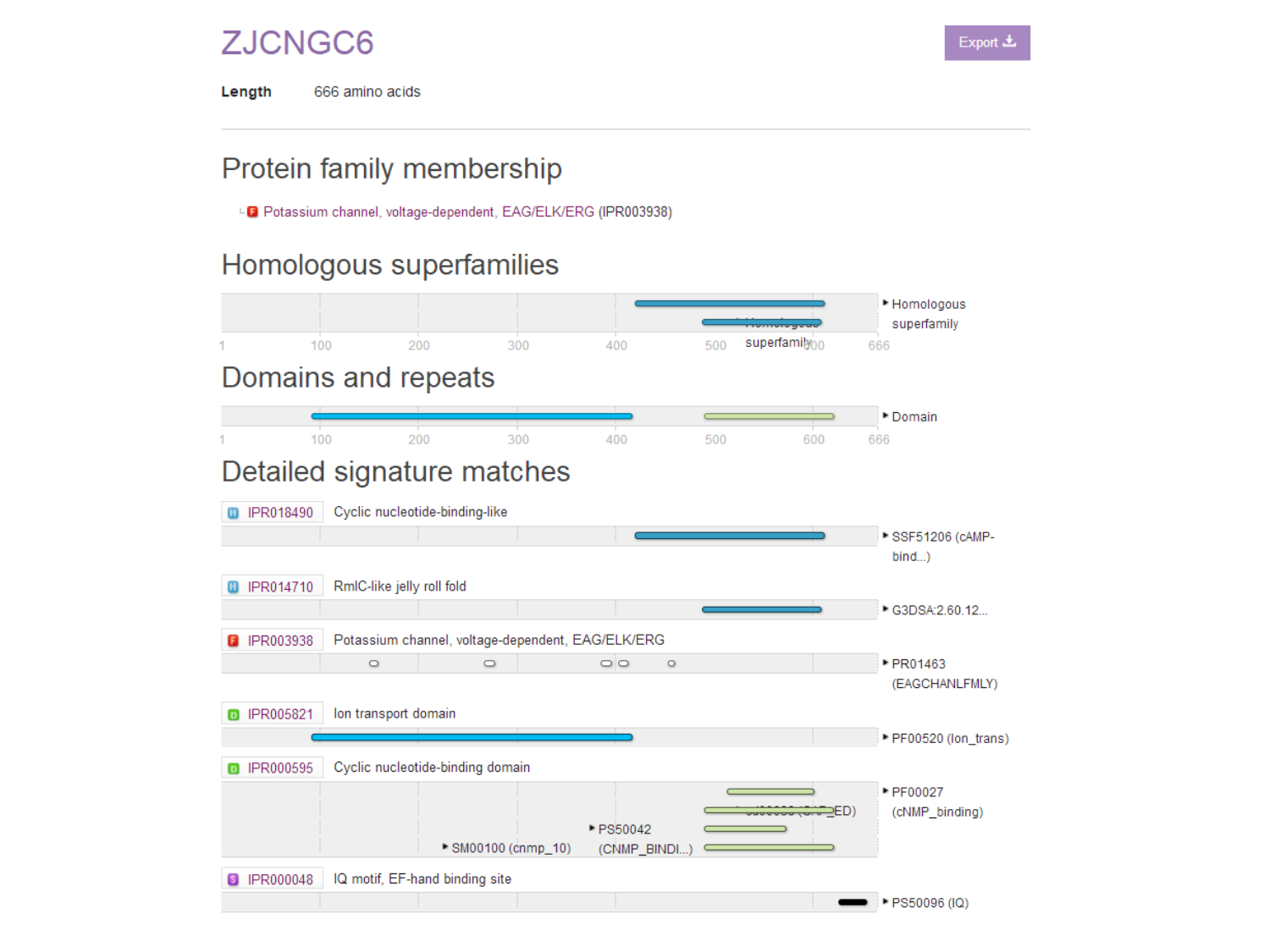

## Slide 14
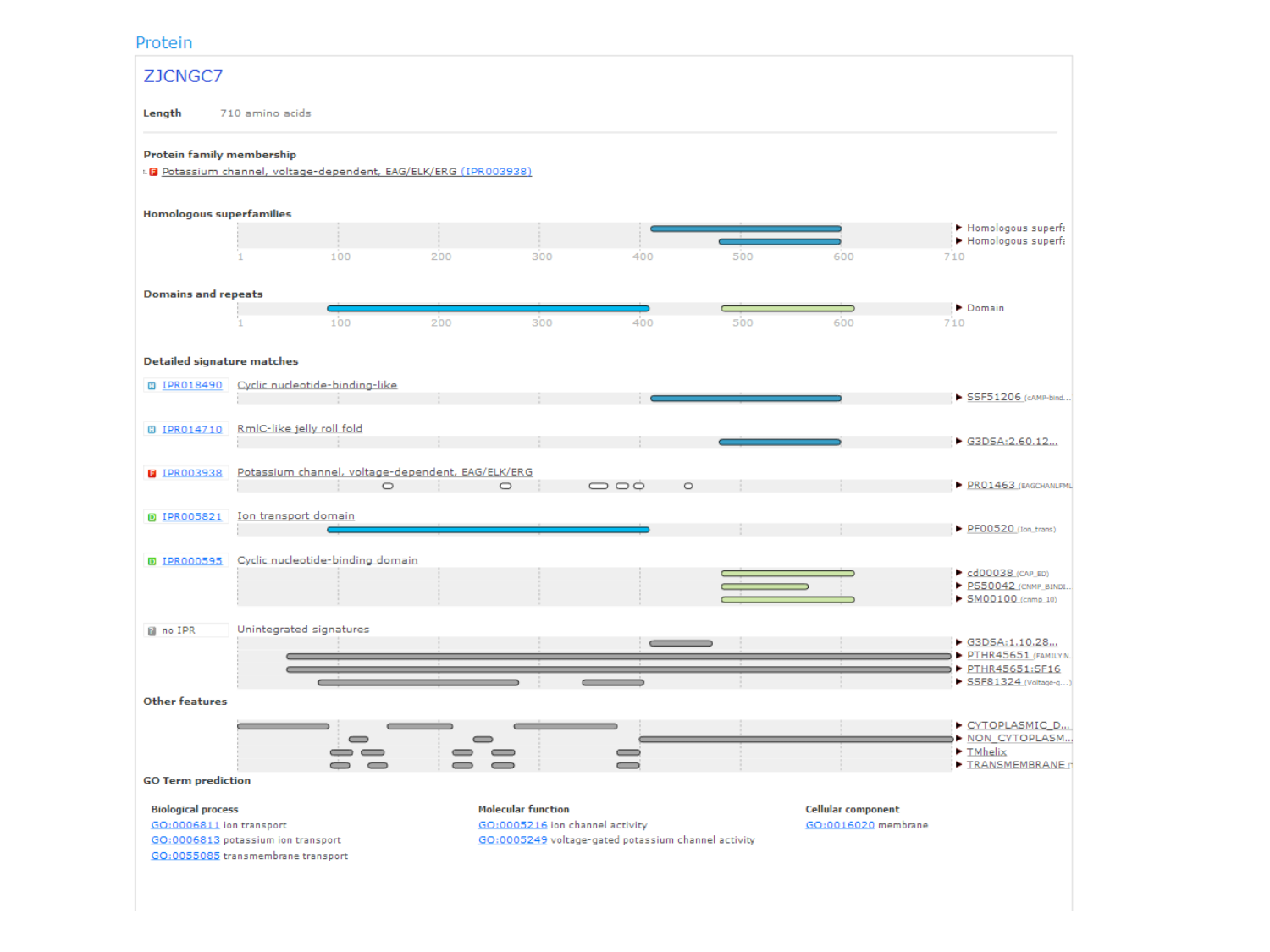

## Slide 15
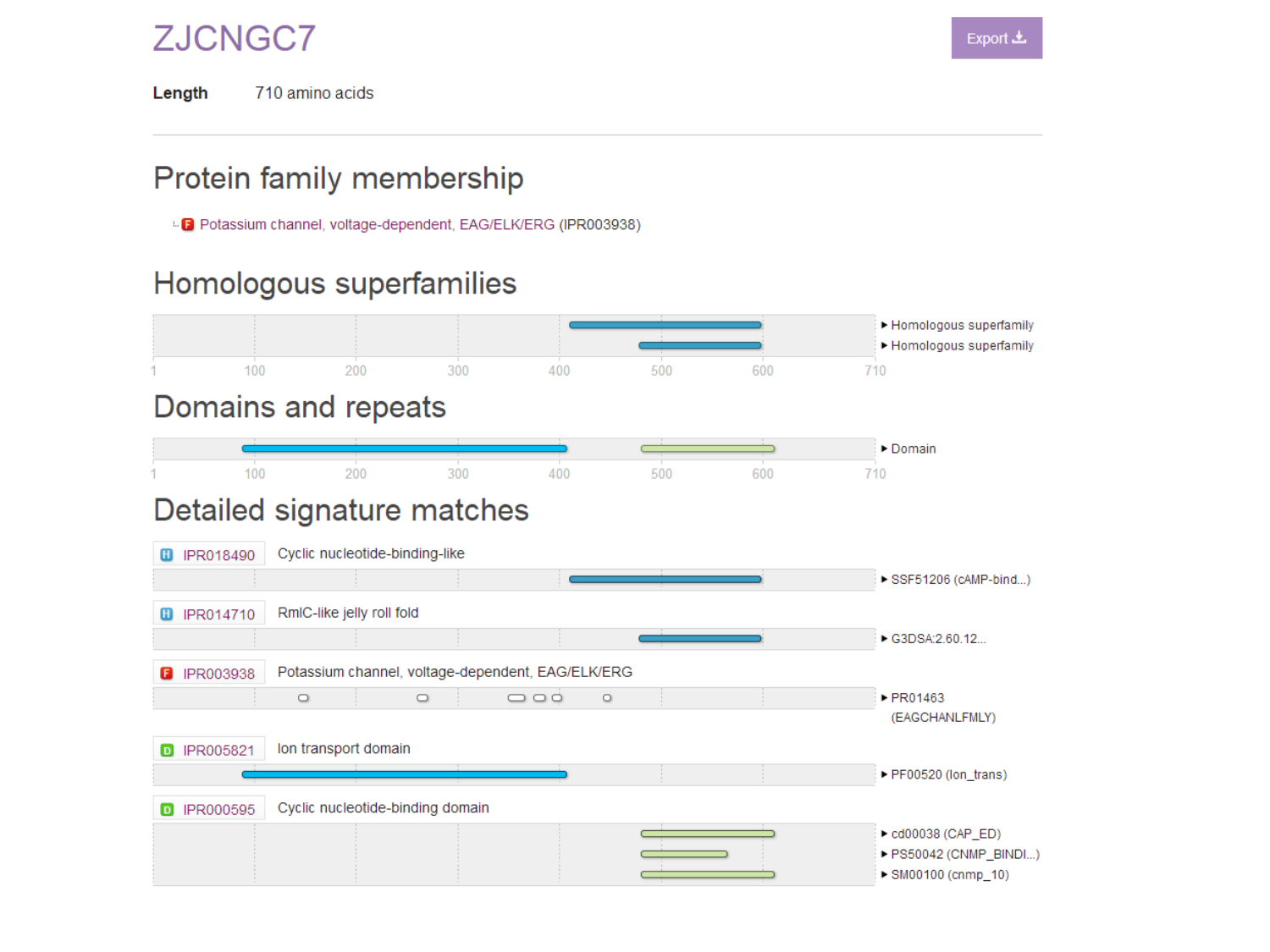

## Slide 16
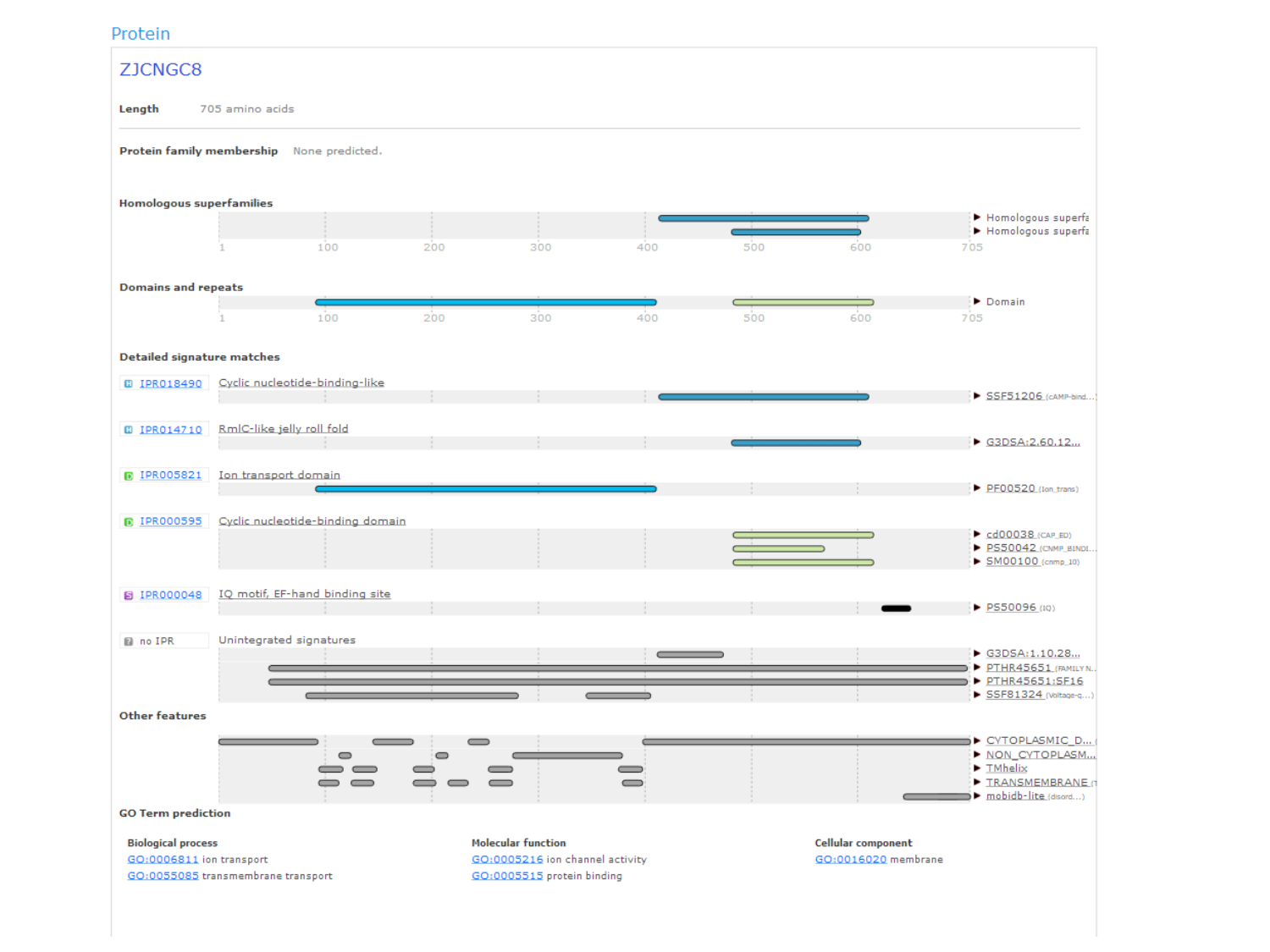

## Slide 17
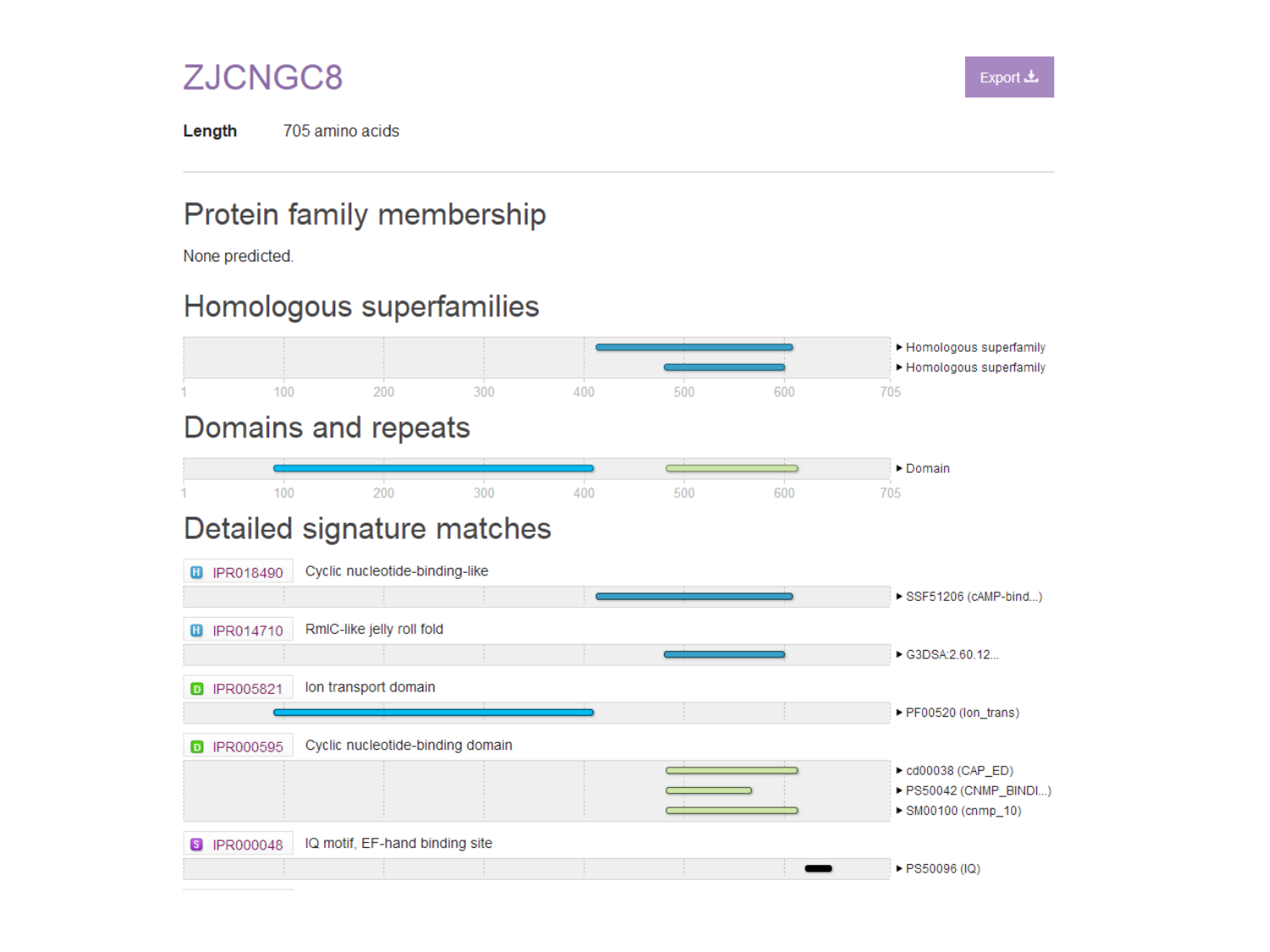

## Slide 18
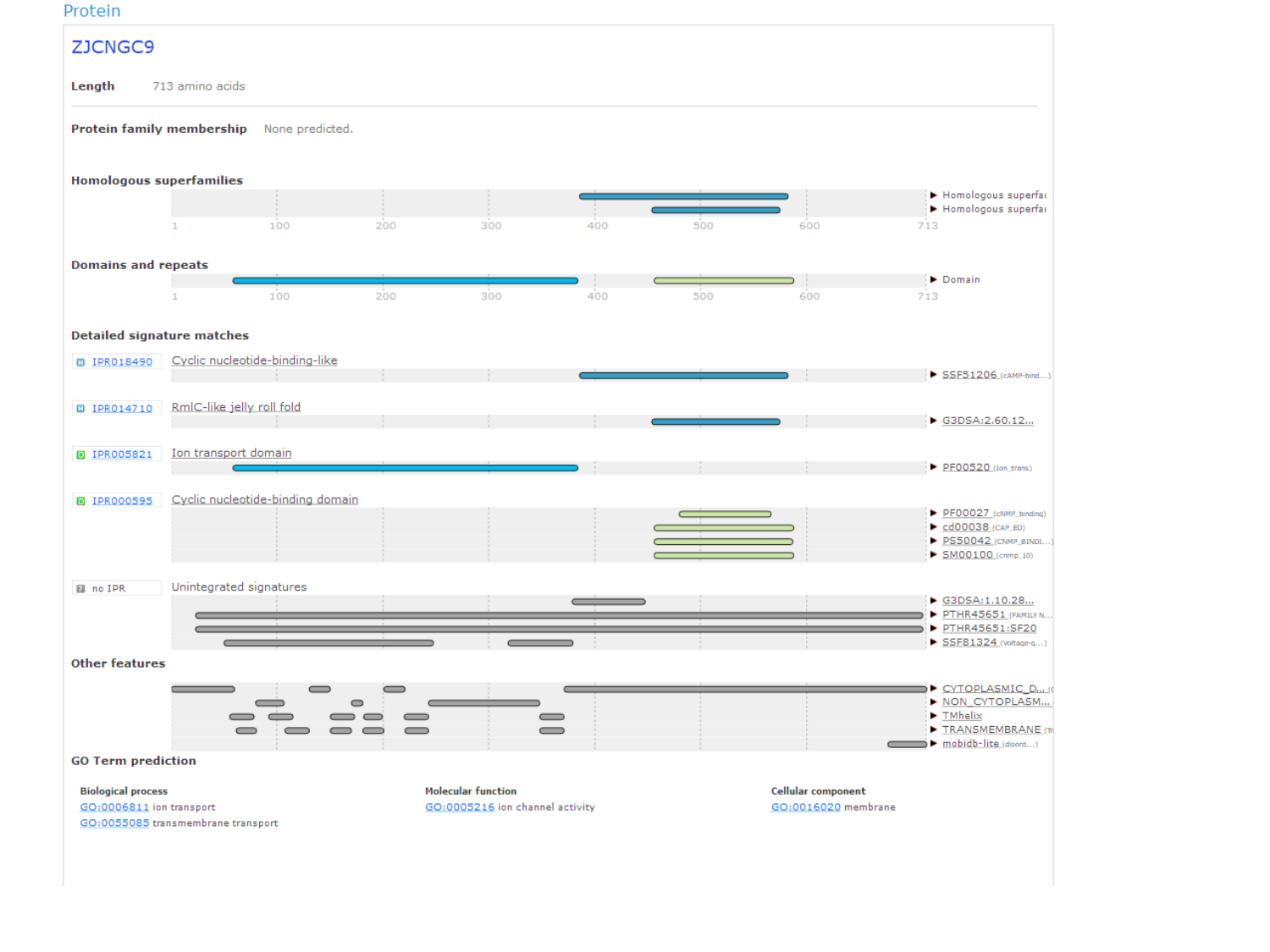

## Slide 19
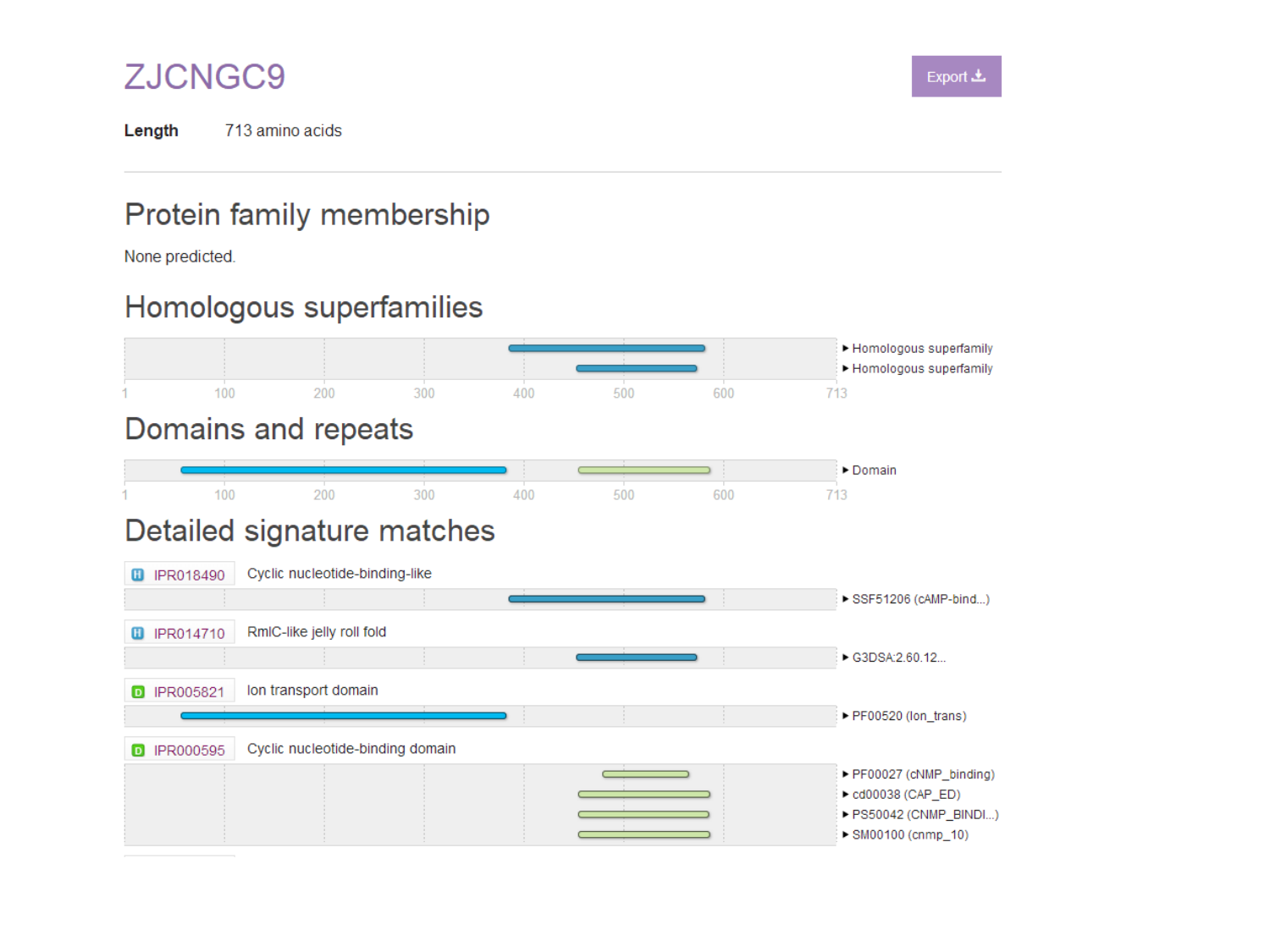

## Slide 20
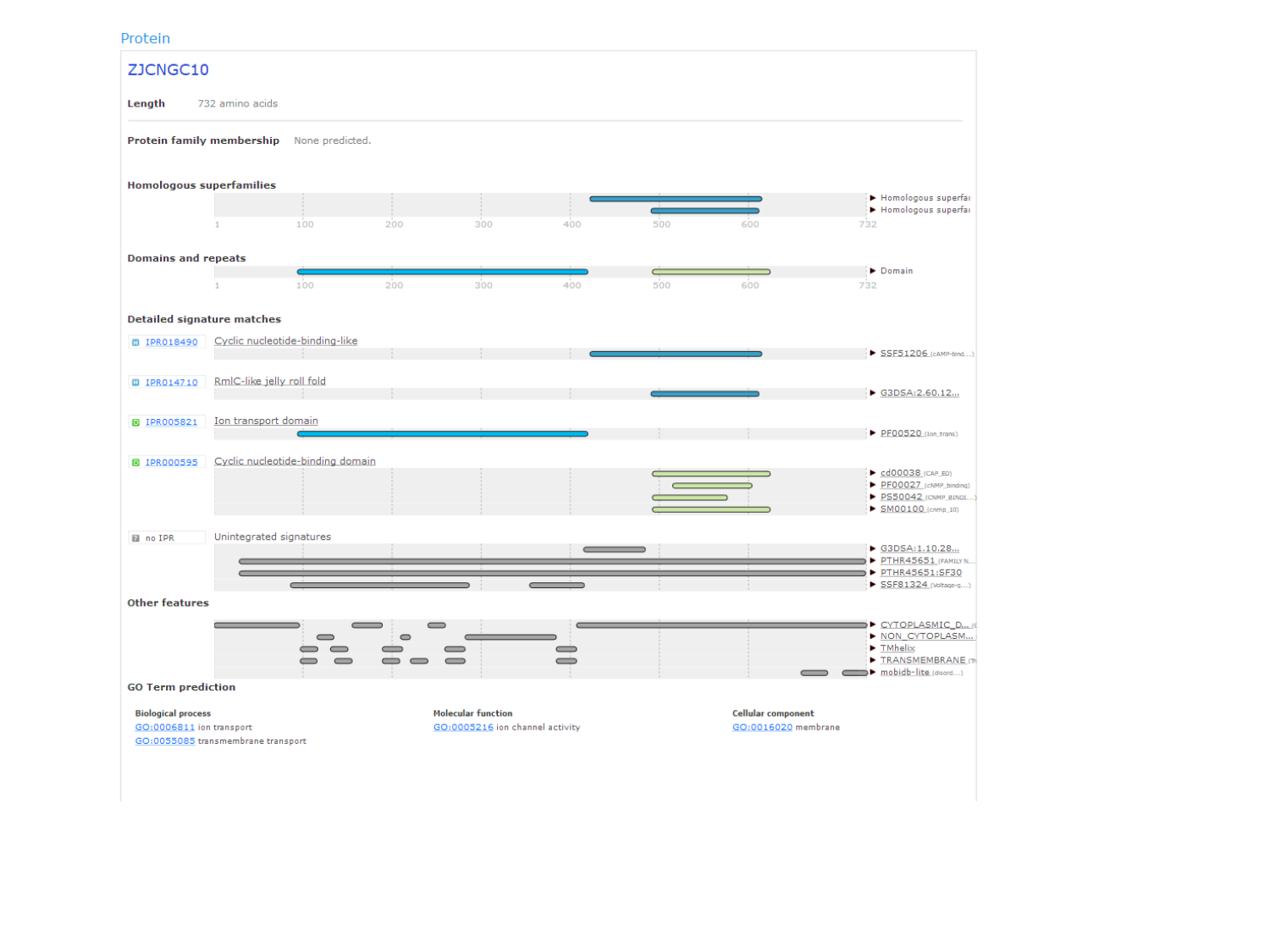

## Slide 21
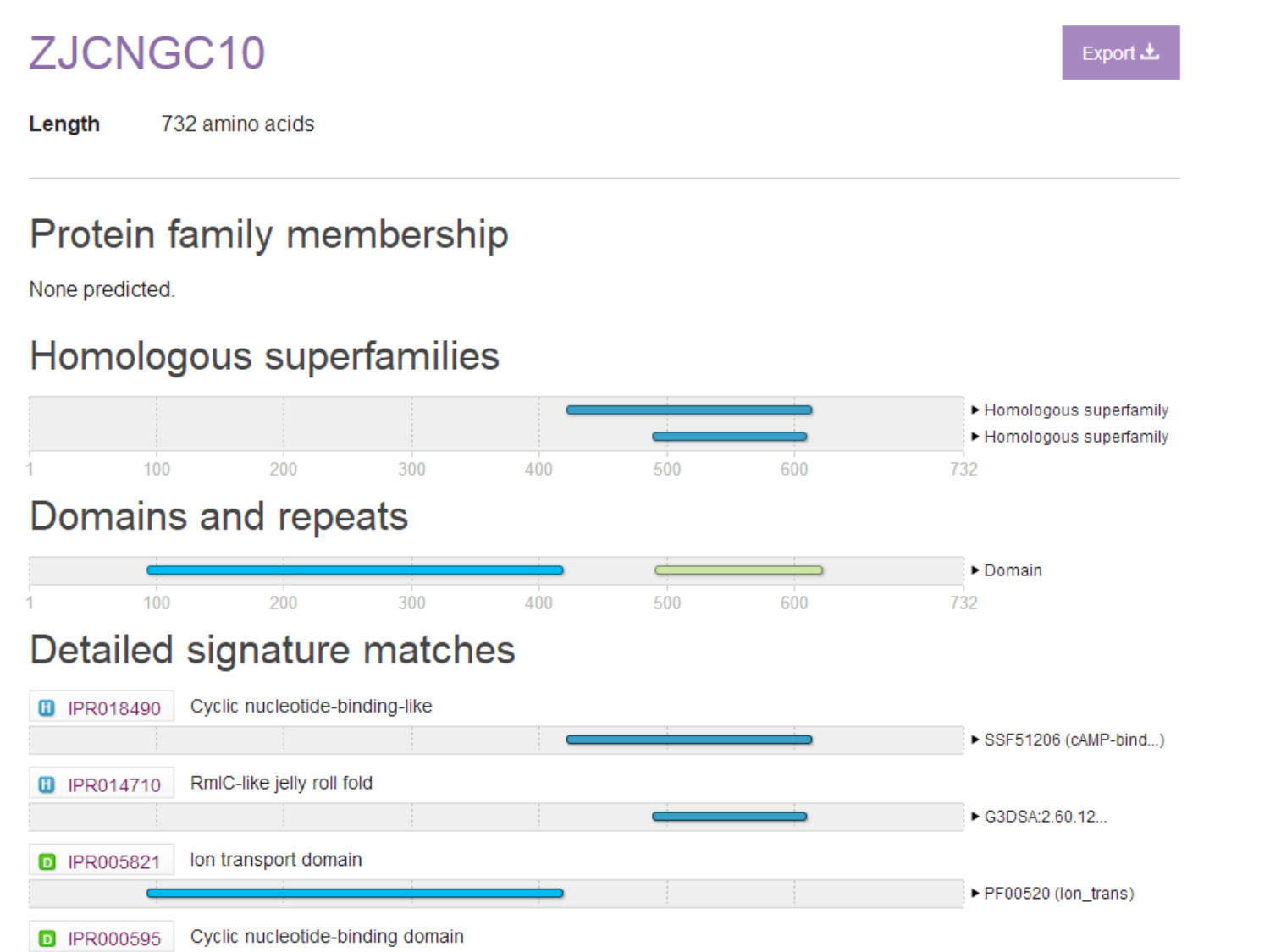

## Slide 22
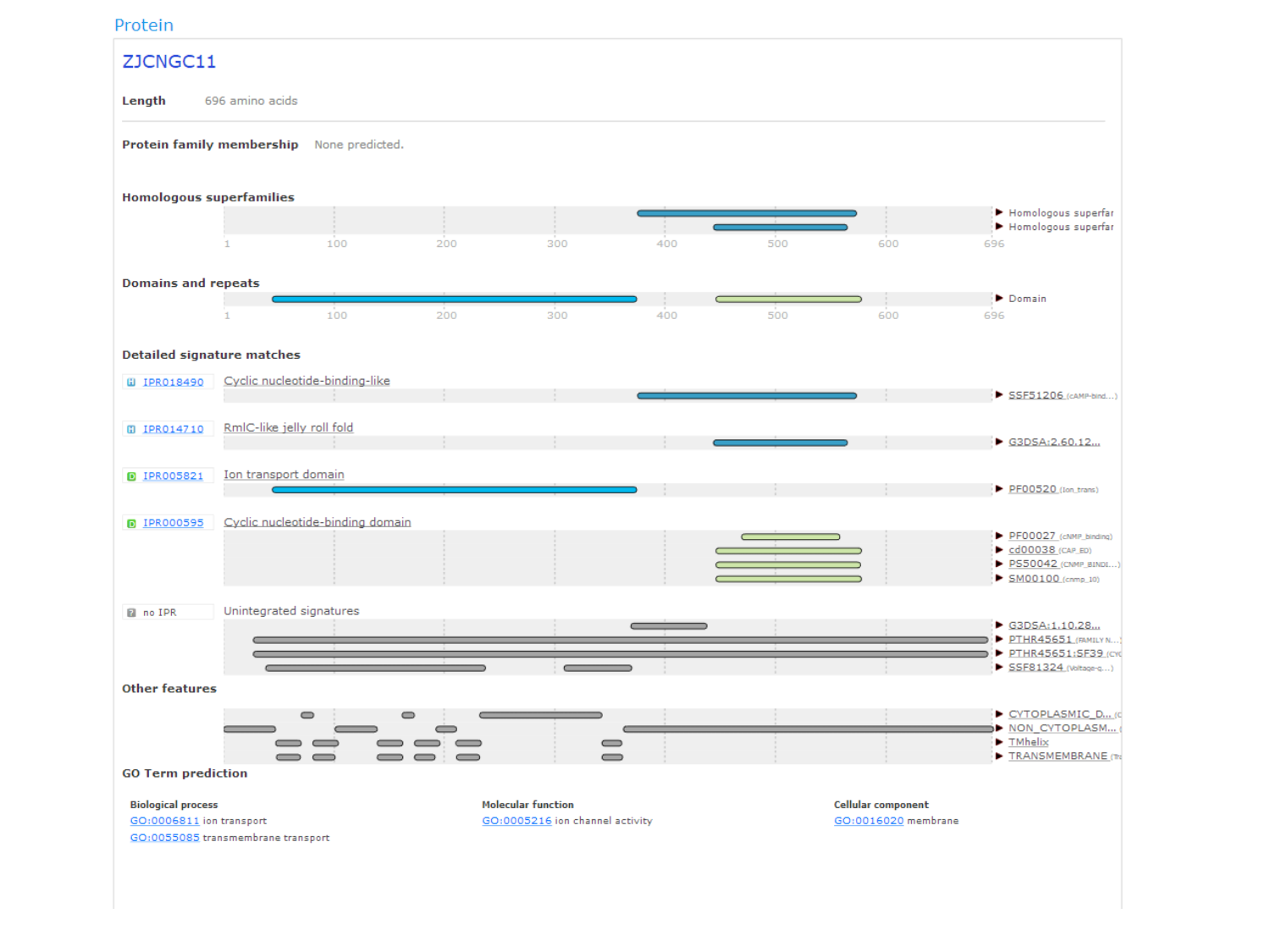

## Slide 23
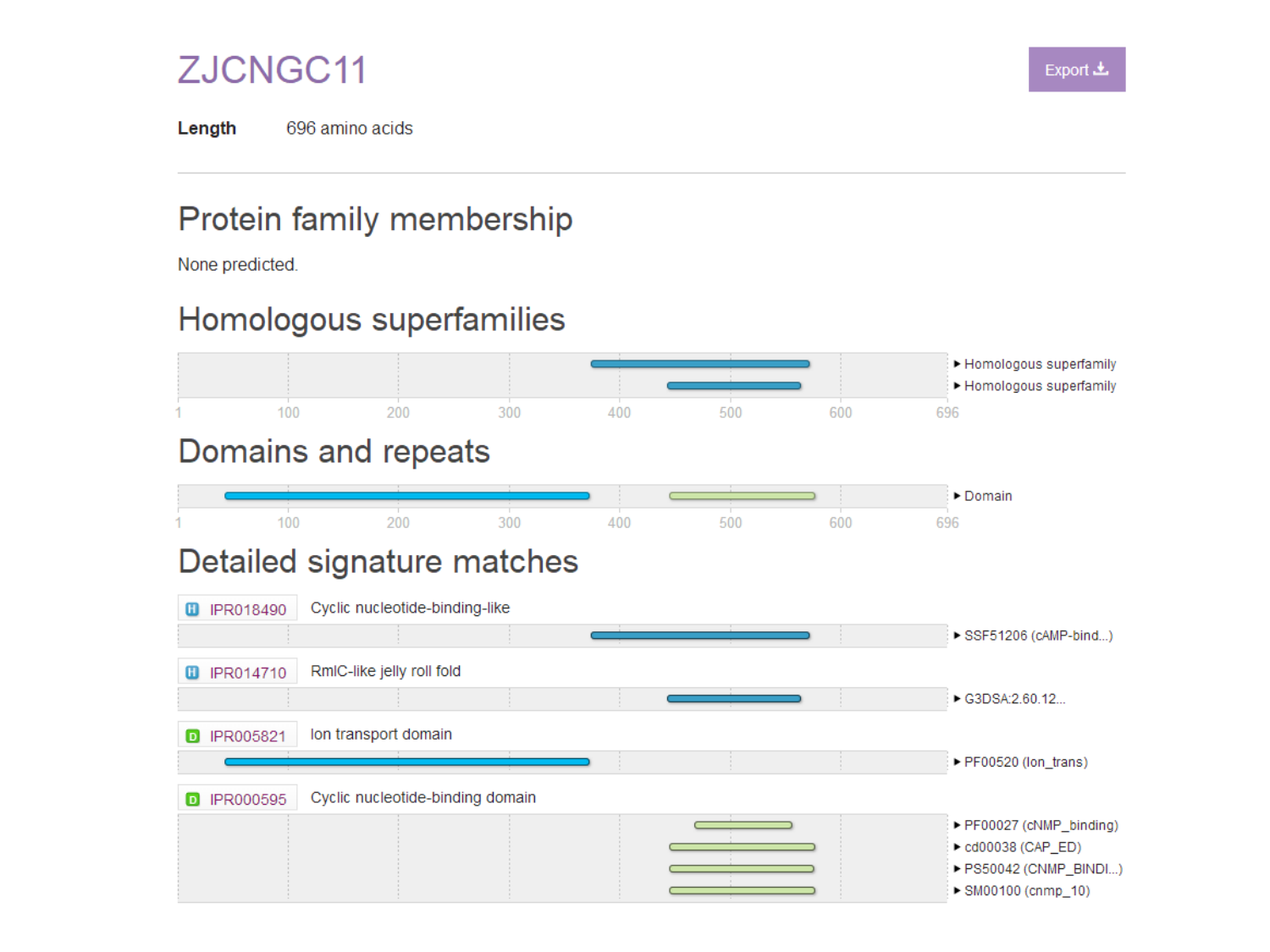

## Slide 24
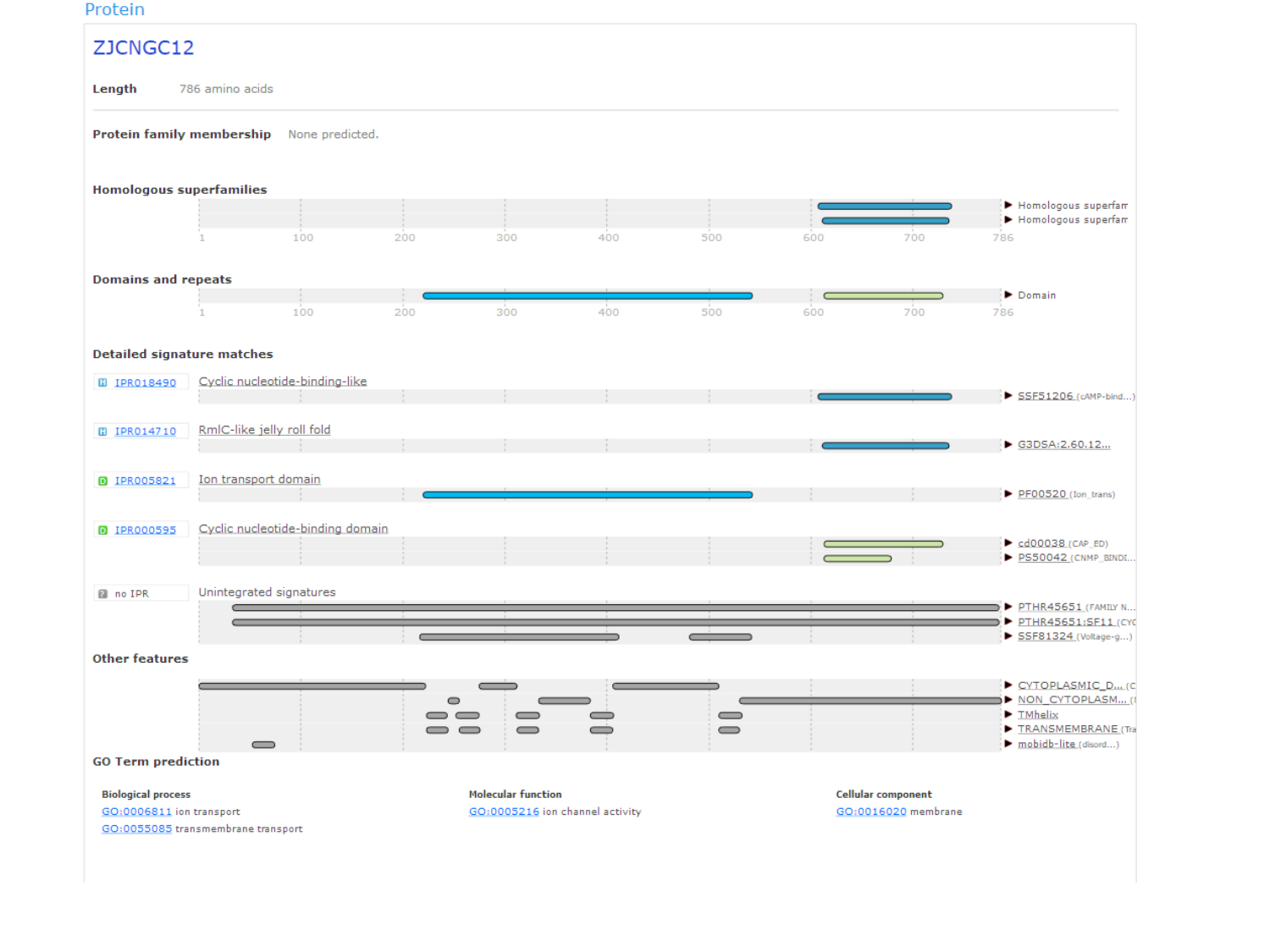

## Slide 25
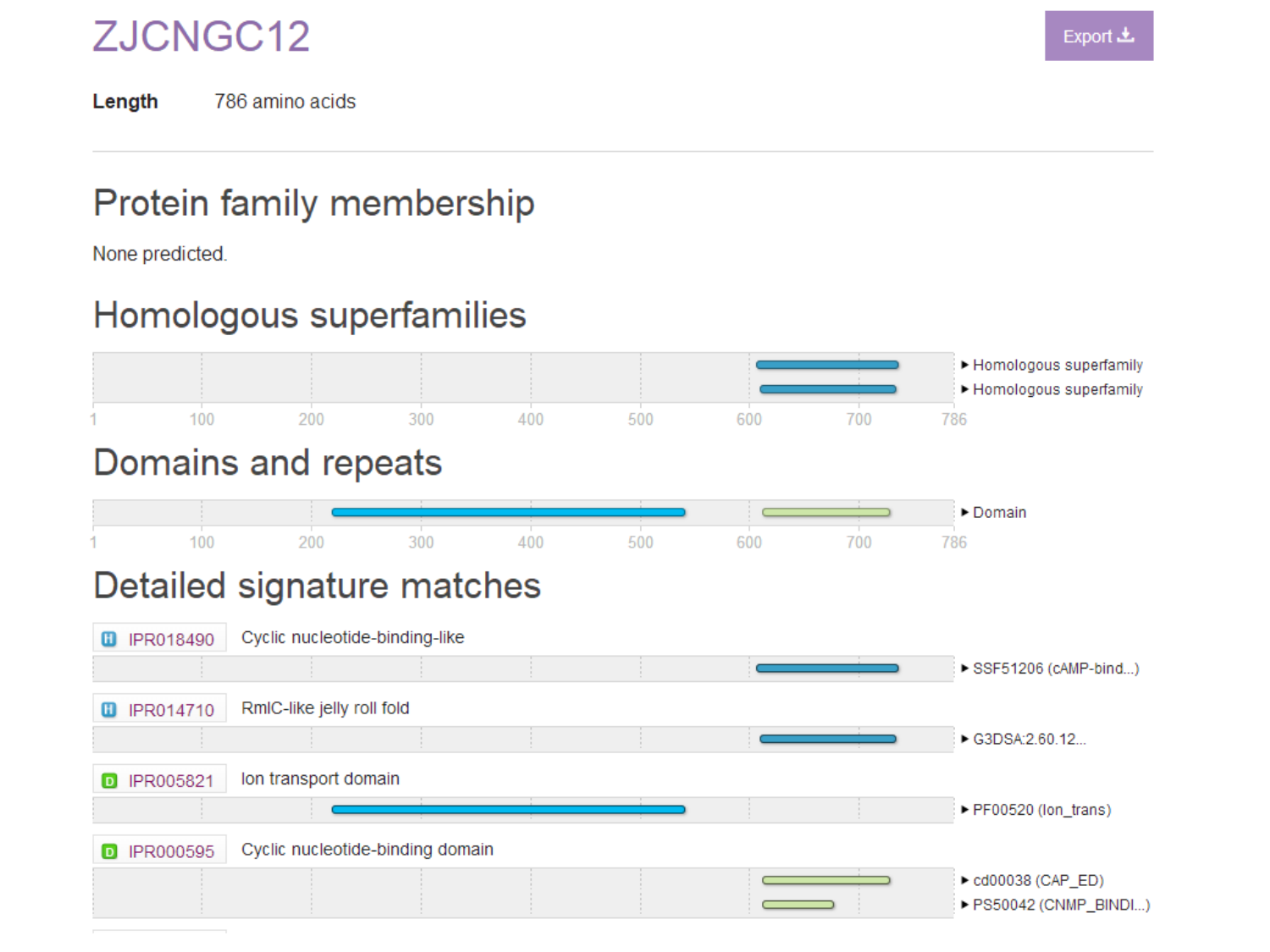

## Slide 26
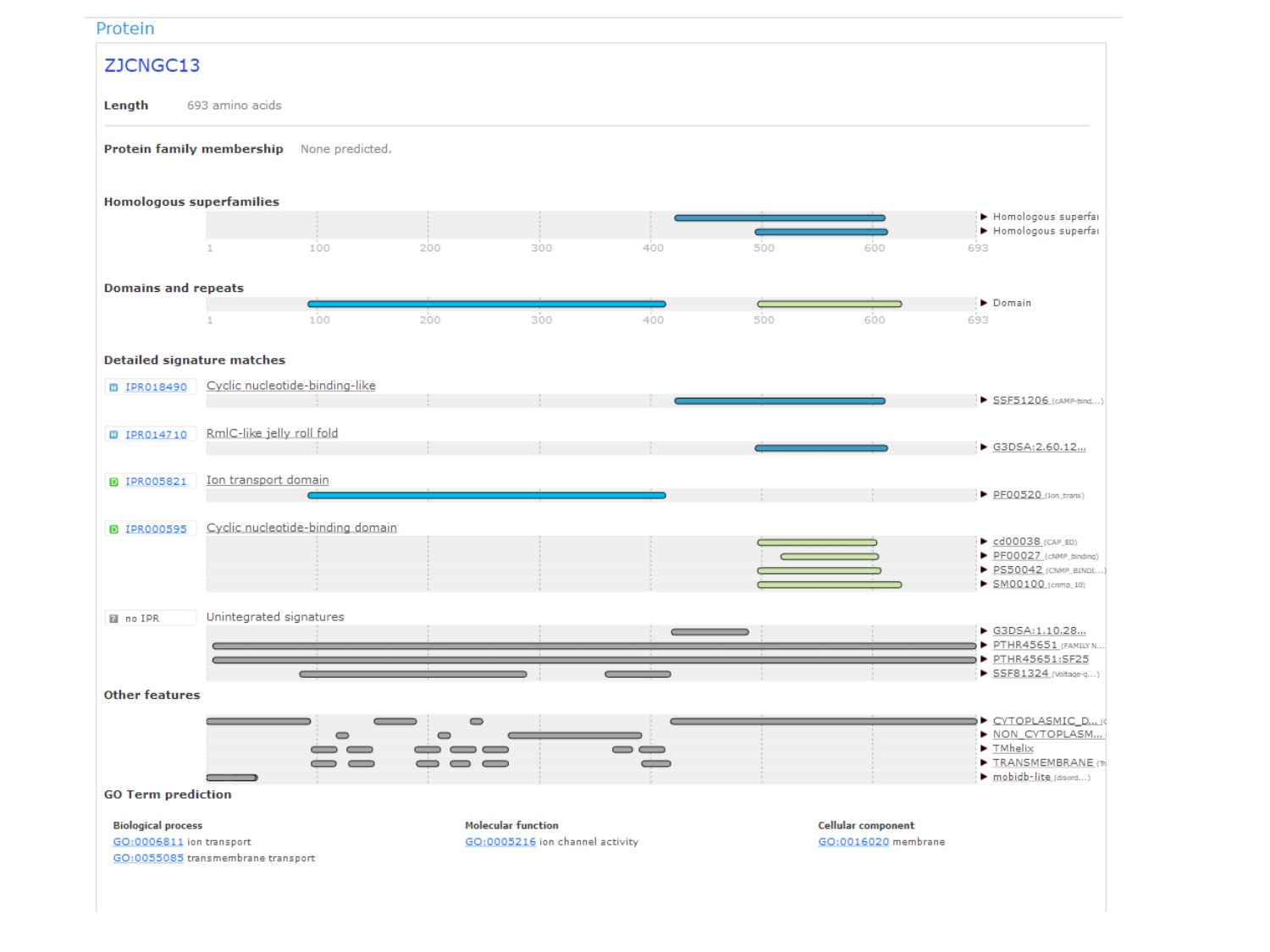

## Slide 27
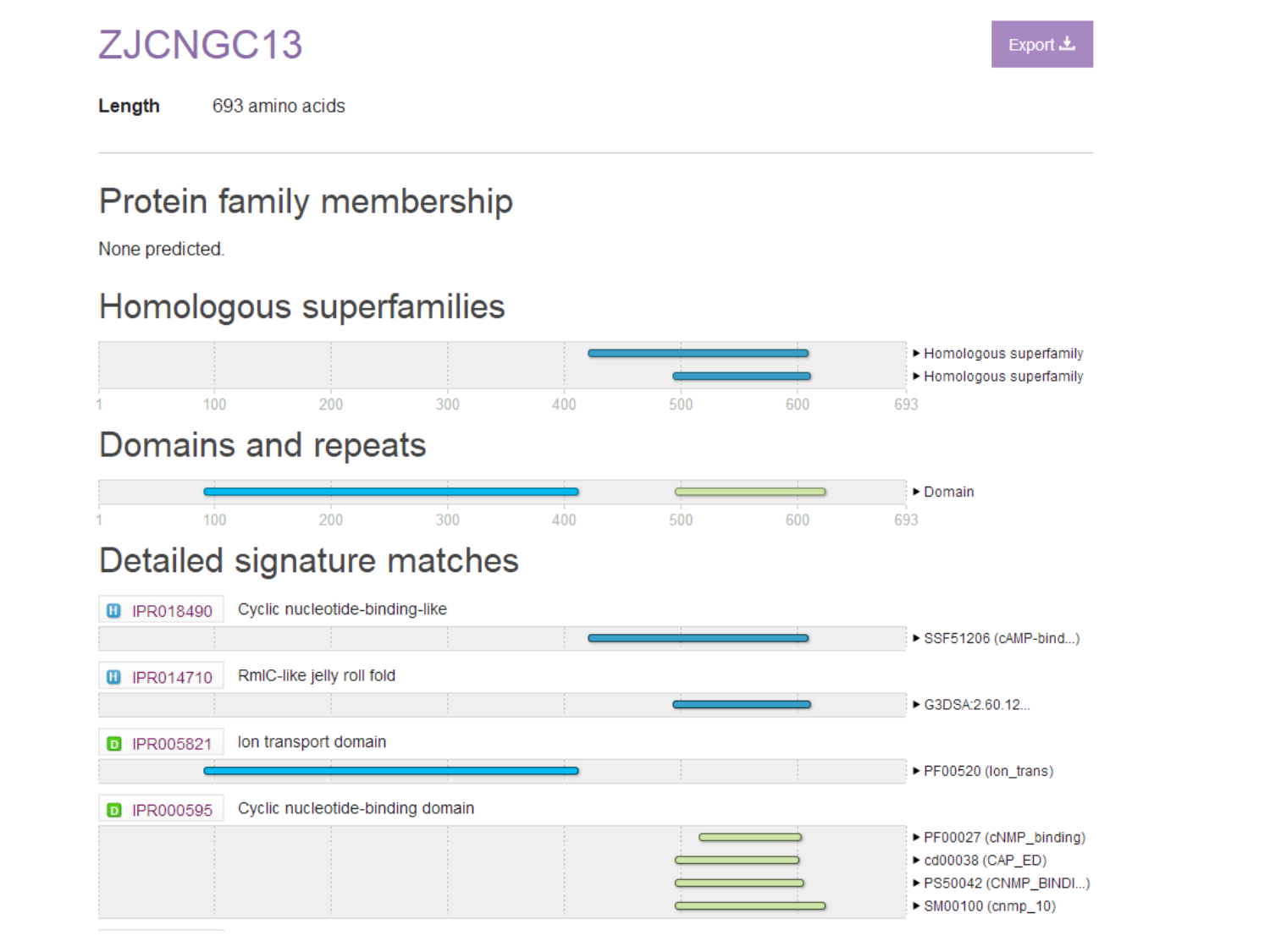

## Slide 28
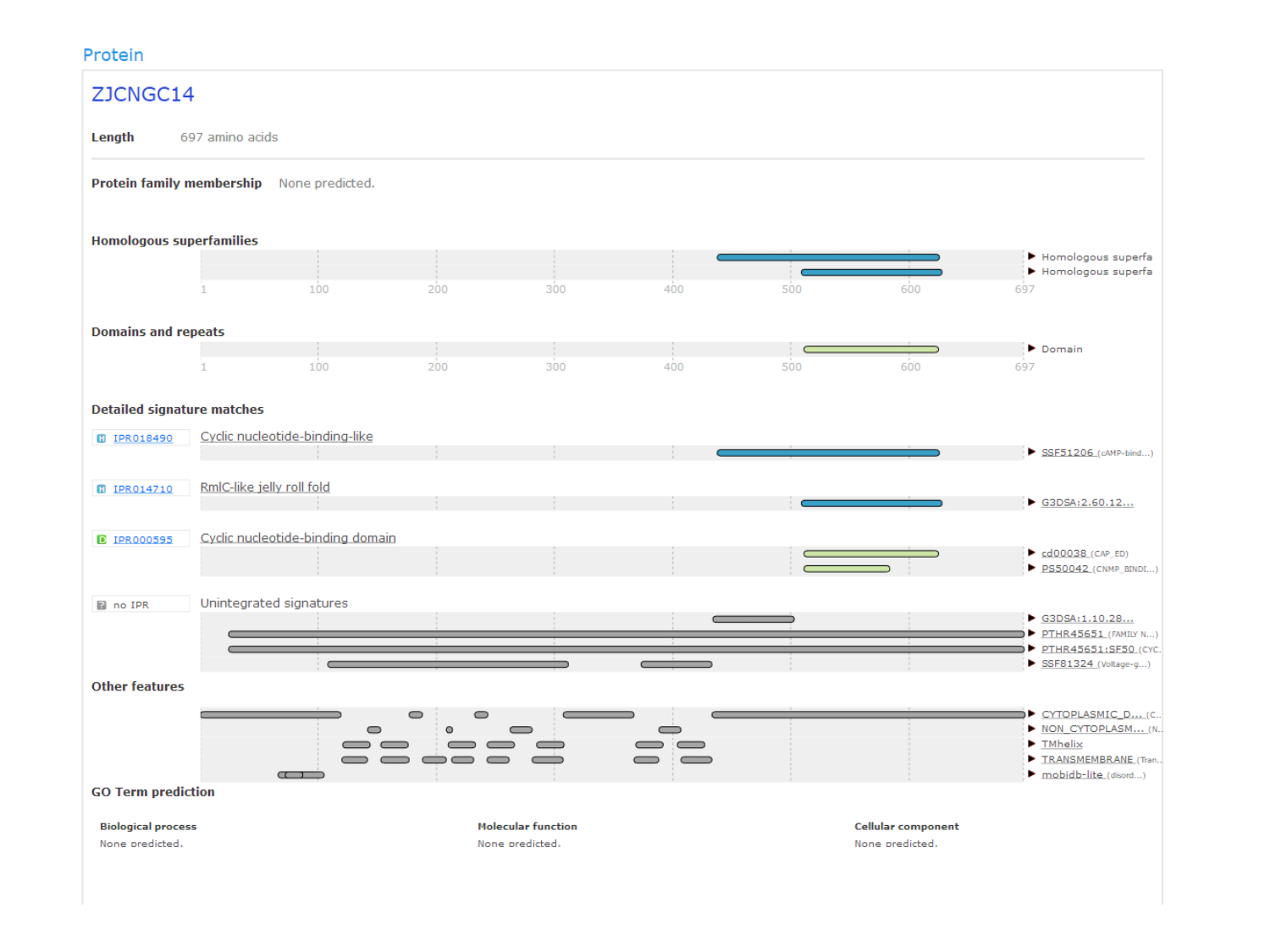

## Slide 29
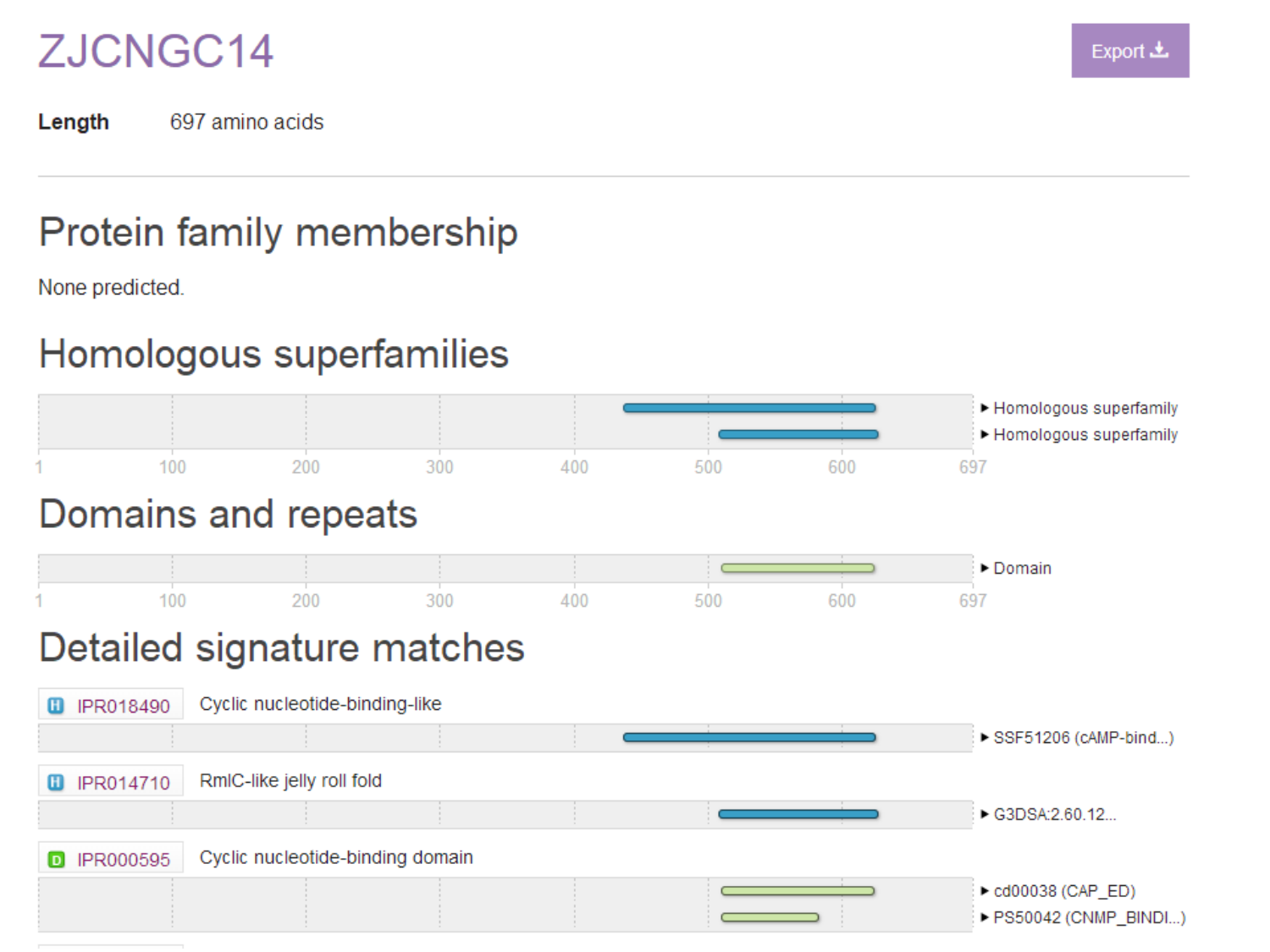

## Slide 30
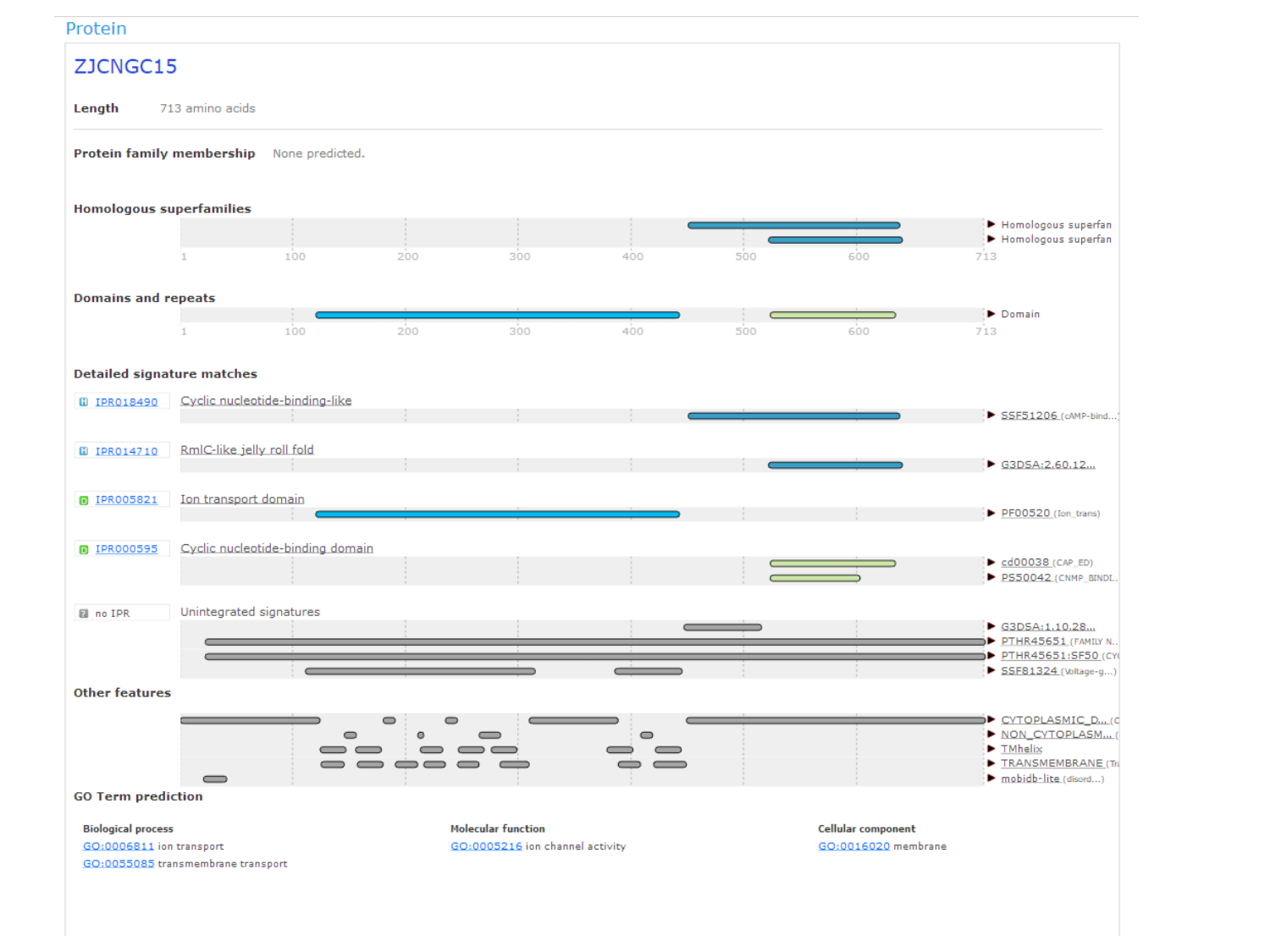

## Slide 31
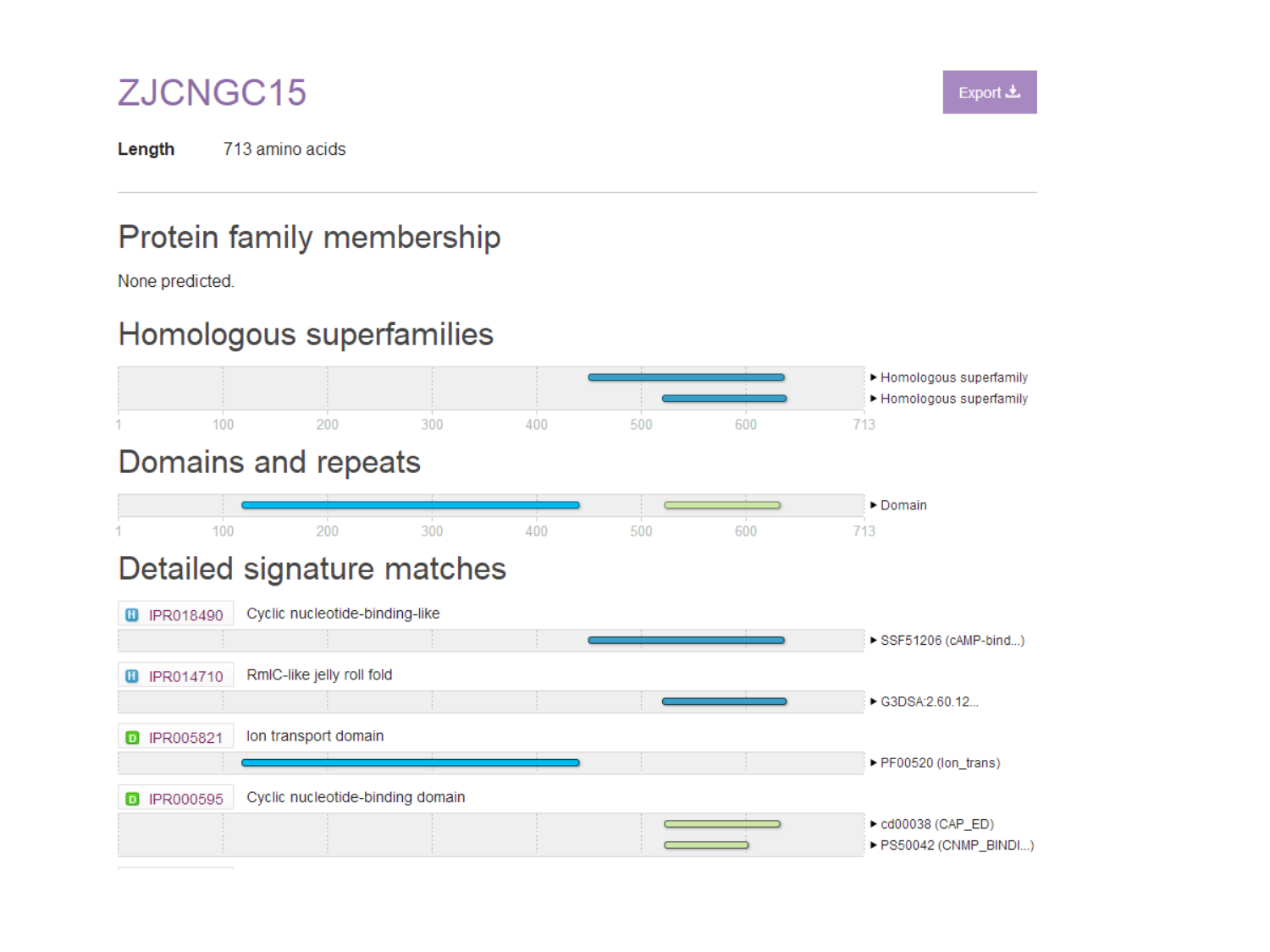

Supplement: Supplementary file 3 — Additional file S3. Domain analysis of ZjCNGCs by using the InterProScan program. [file 12864_2020_6601_MOESM3_ESM.pptx]
