# Supplementary material for: Genome-wide identification of CNGC genes in Chinese jujube (Ziziphus jujuba Mill.) and ZjCNGC2 mediated signalling cascades in response to cold stress
Source: BMC Genomics. 2020 Mar 2;21:191. doi: 10.1186/s12864-020-6601-5 (PMC7053155; doi:10.1186/s12864-020-6601-5)
Supplement: Supplementary file 4 — Additional file S4. Domain analysis of ZjCNGCs by using the SMART program. [file 12864_2020_6601_MOESM4_ESM.pptx]

## Slide 1
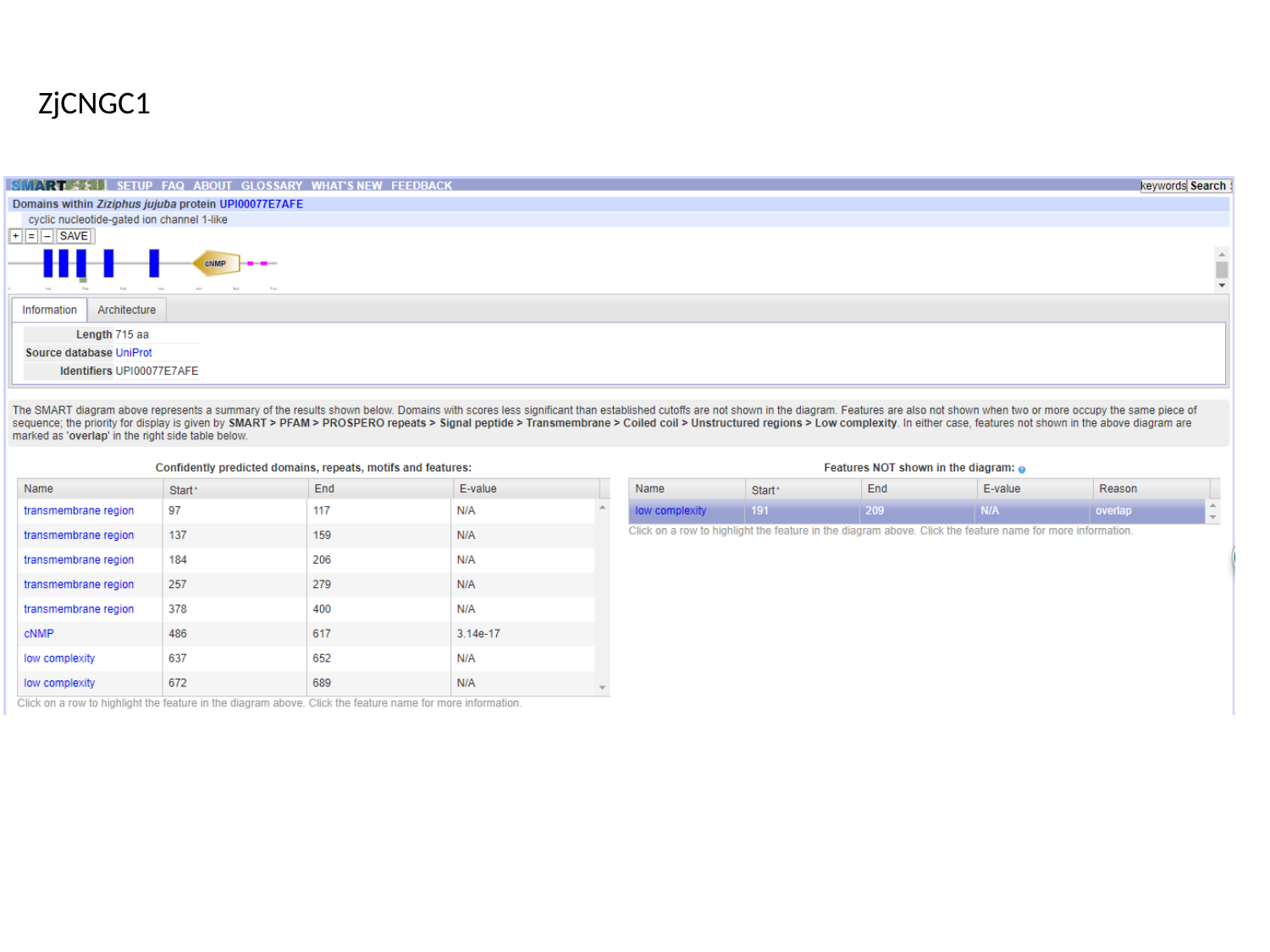

ZjCNGC1

## Slide 2
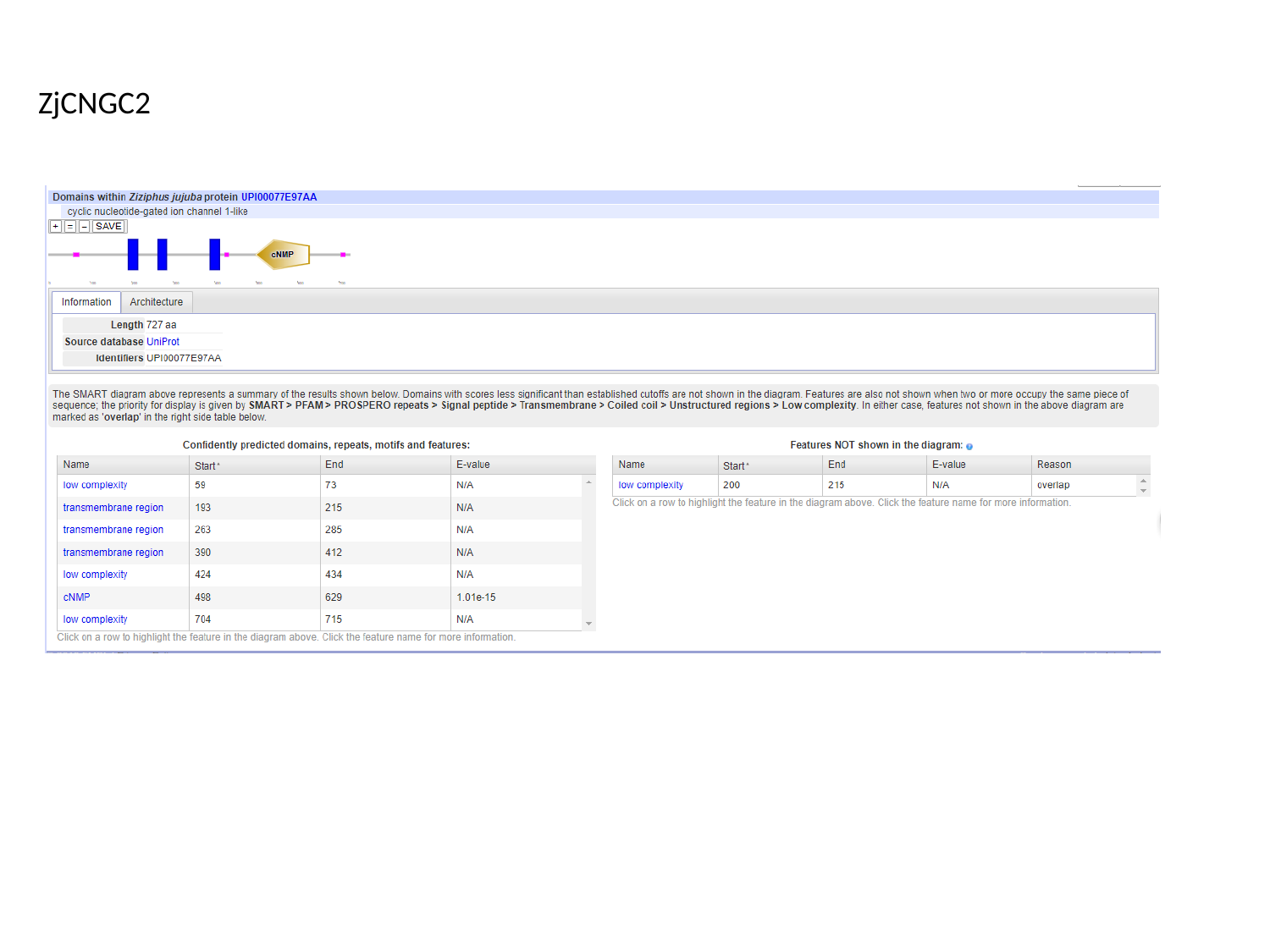

ZjCNGC2

## Slide 3
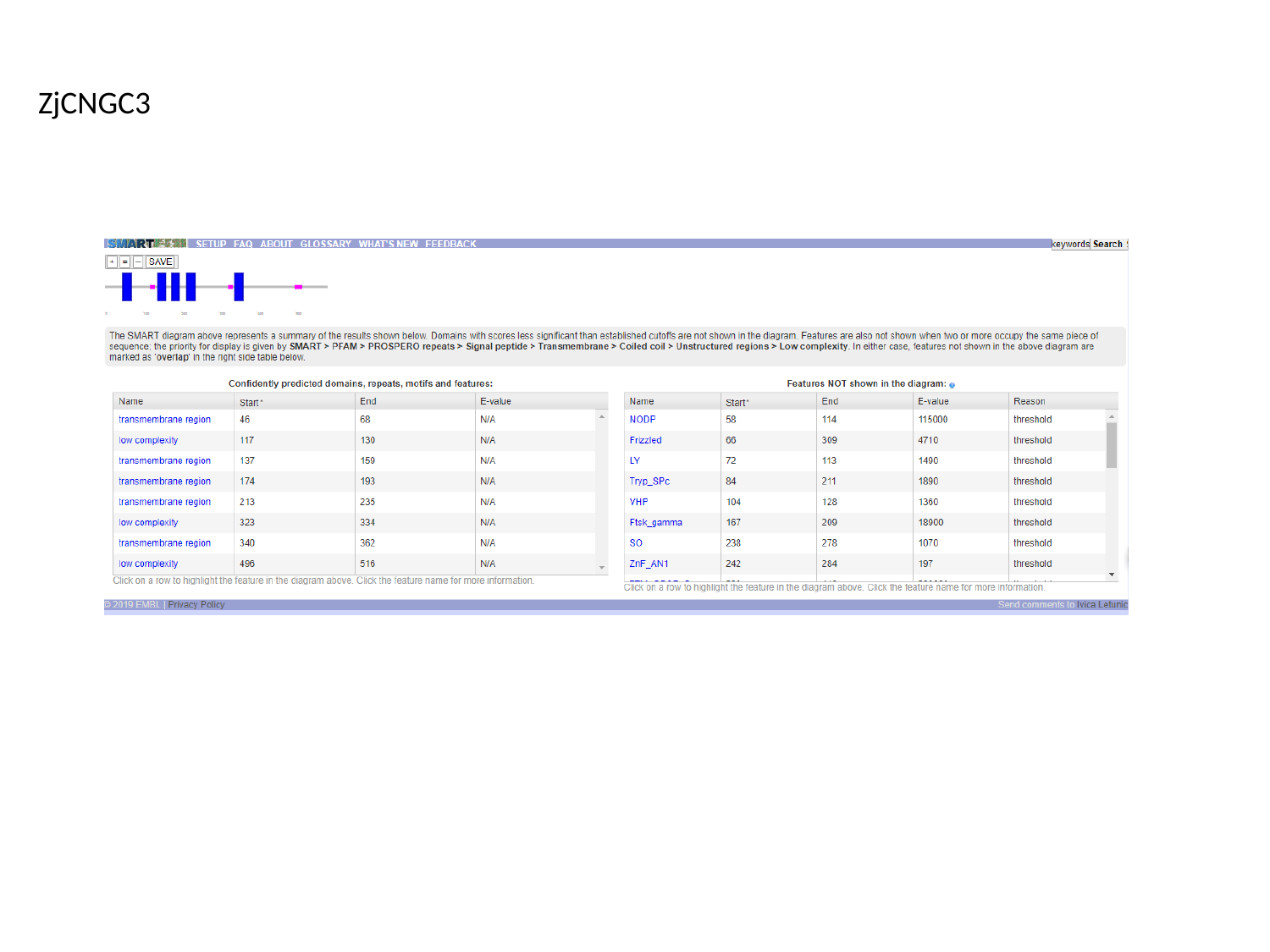

ZjCNGC3

## Slide 4
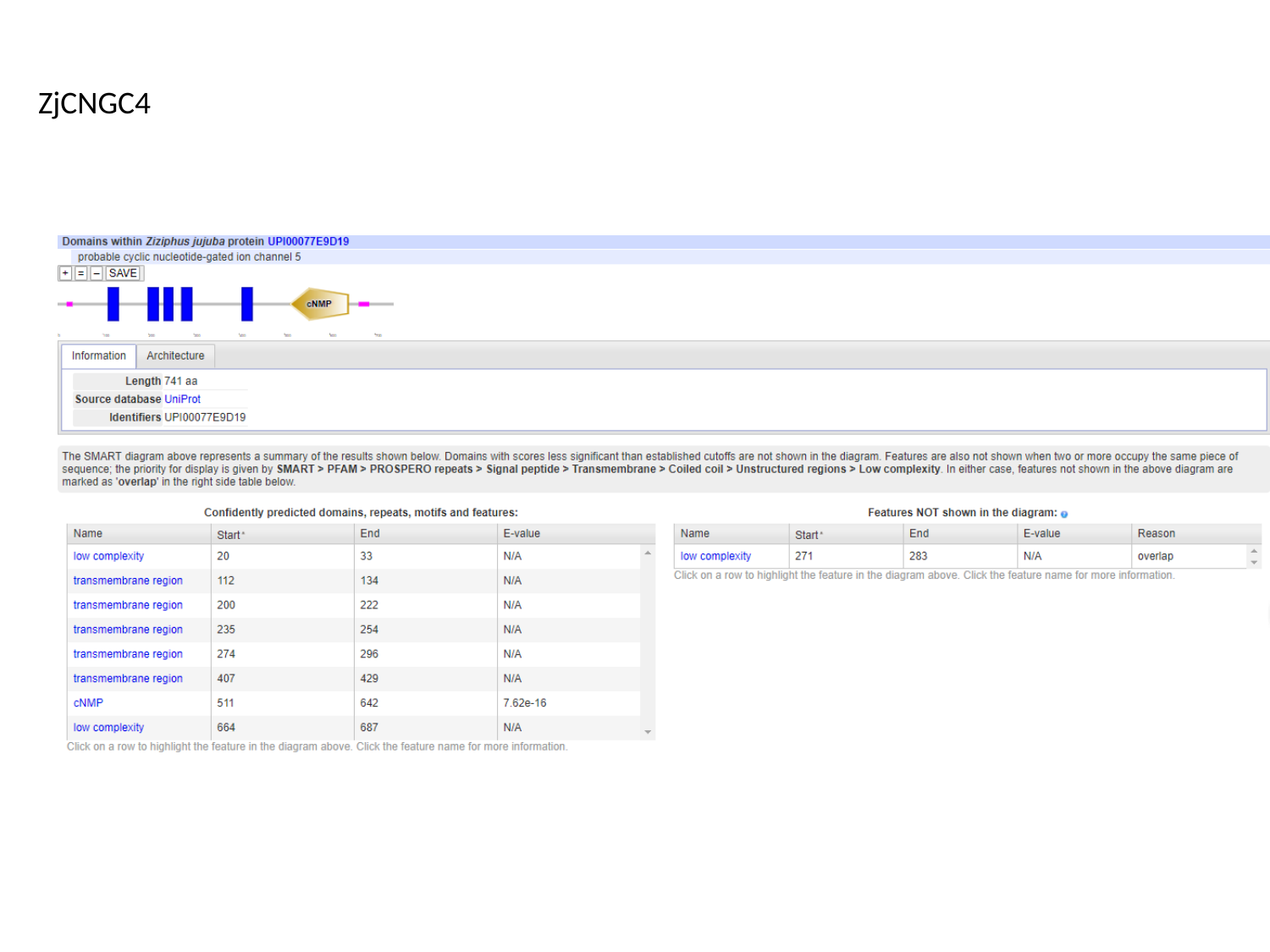

ZjCNGC4

## Slide 5
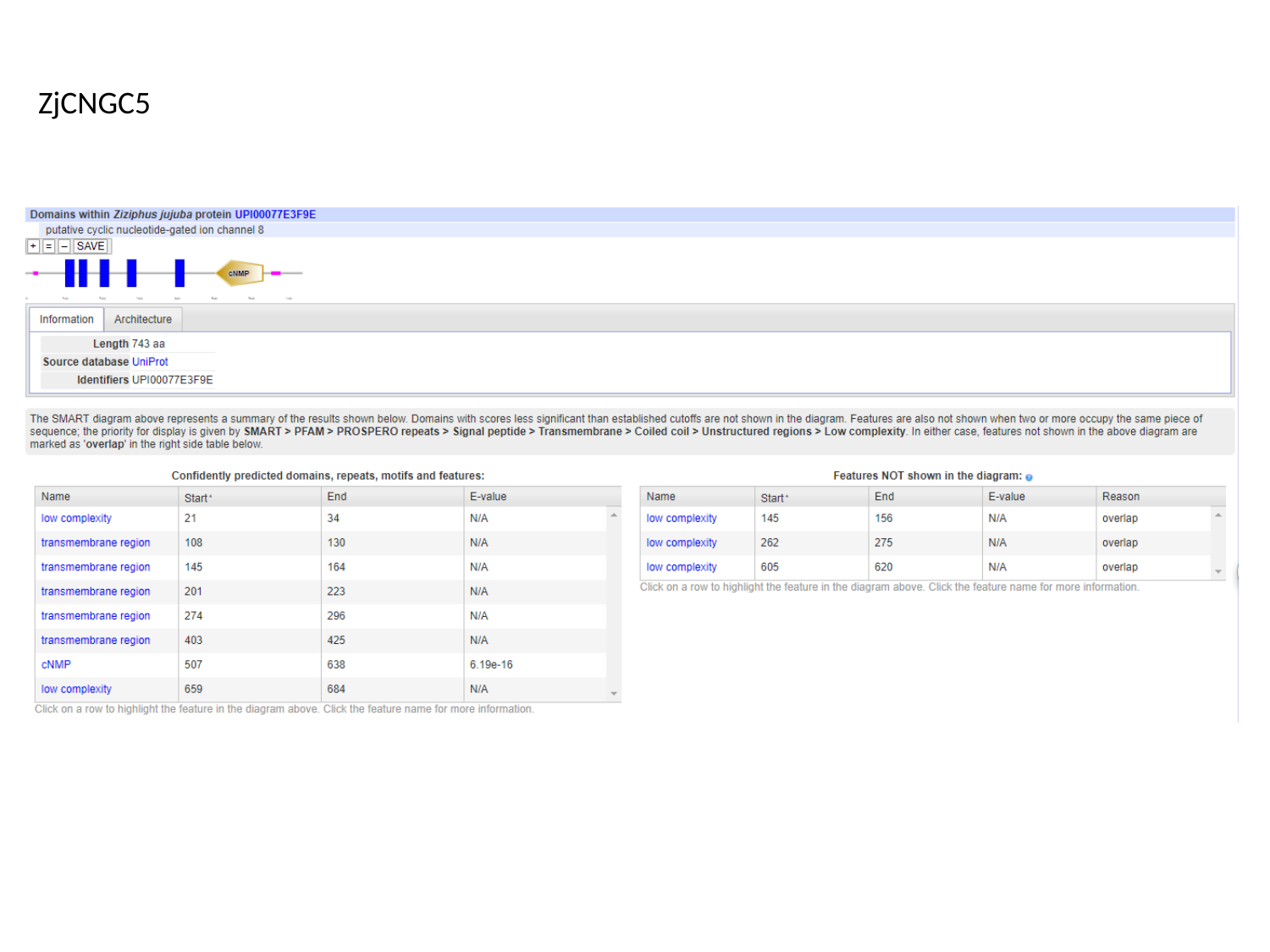

ZjCNGC5

## Slide 6
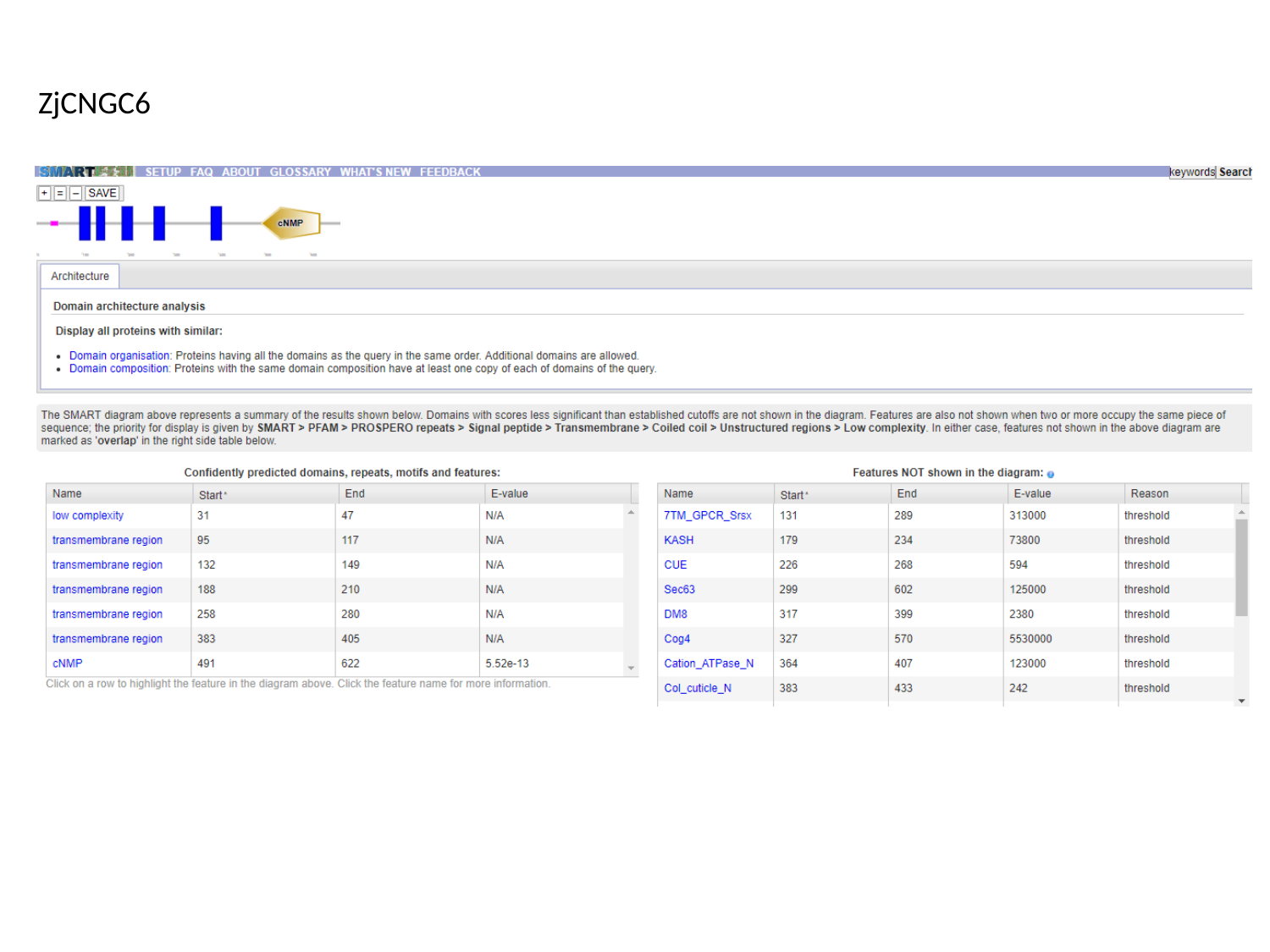

ZjCNGC6

## Slide 7
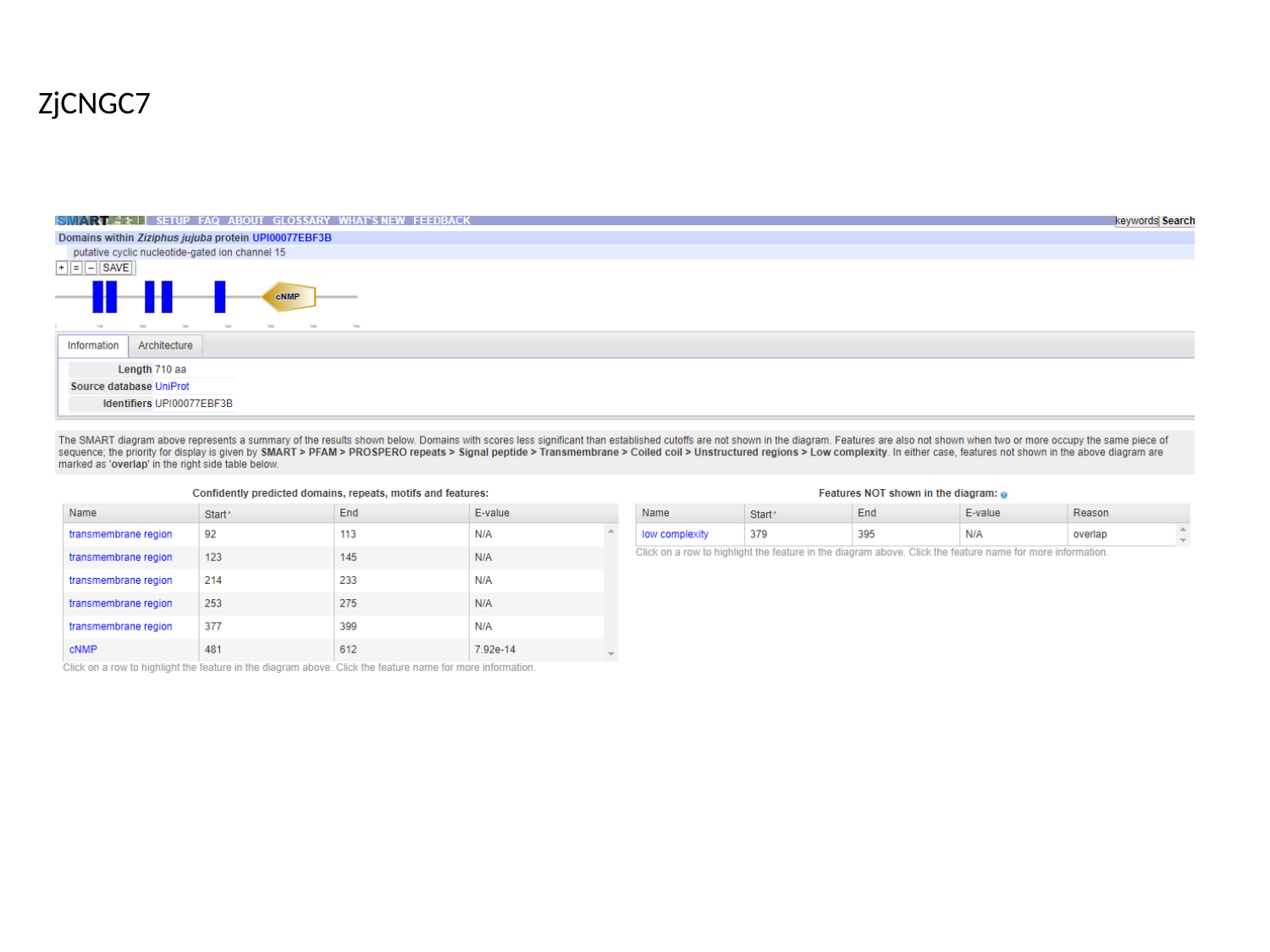

ZjCNGC7

## Slide 8
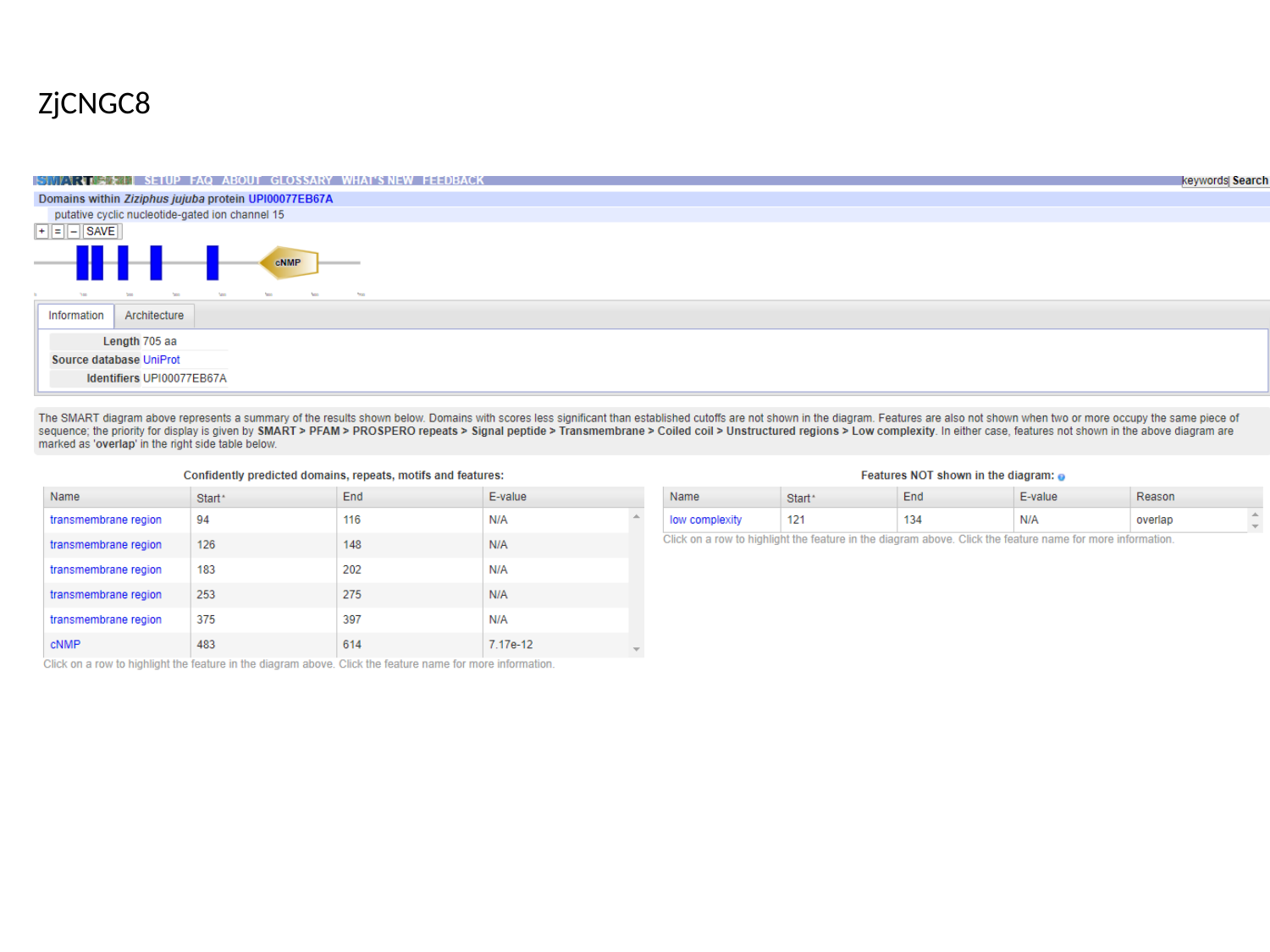

ZjCNGC8

## Slide 9
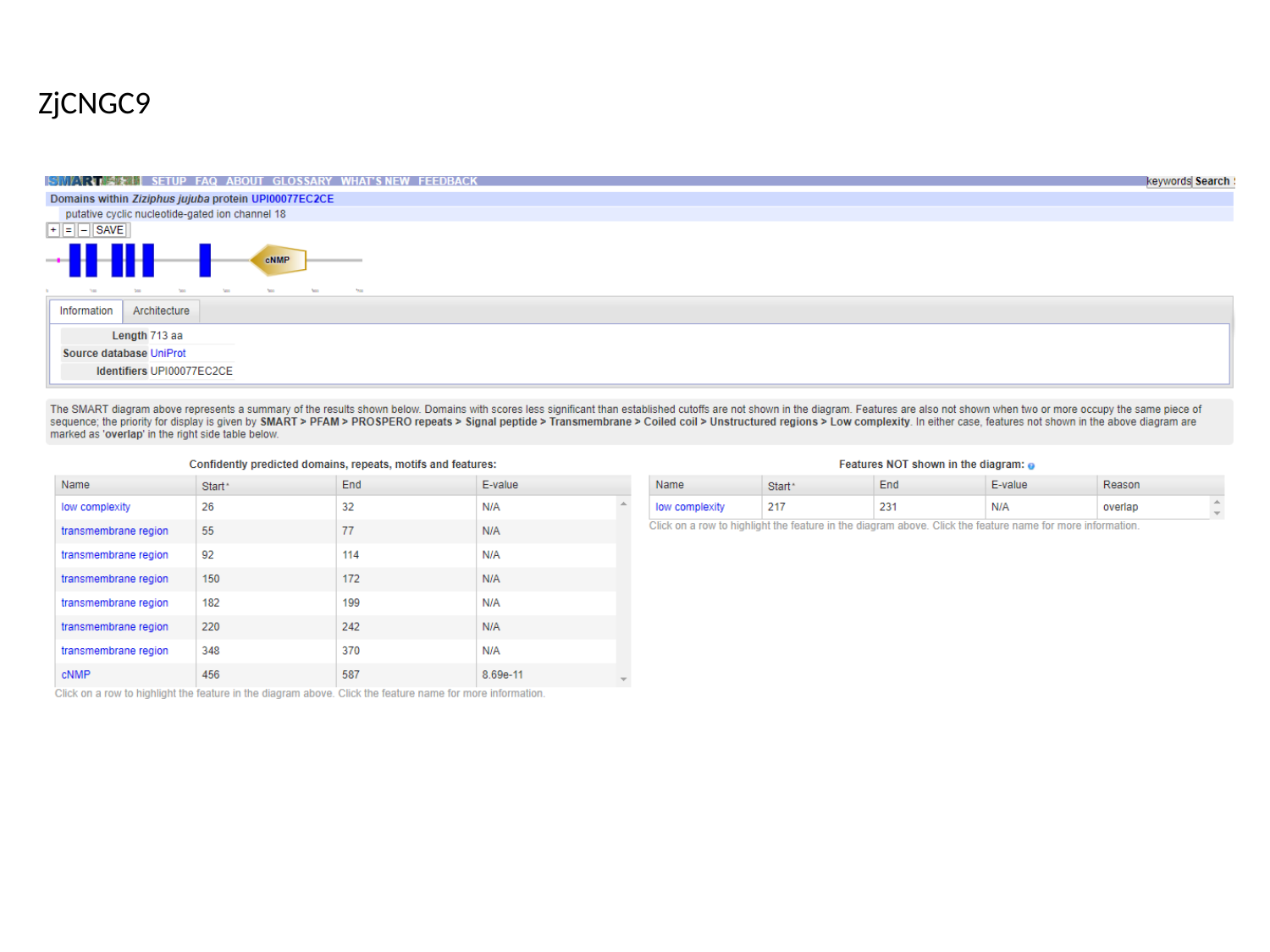

ZjCNGC9

## Slide 10
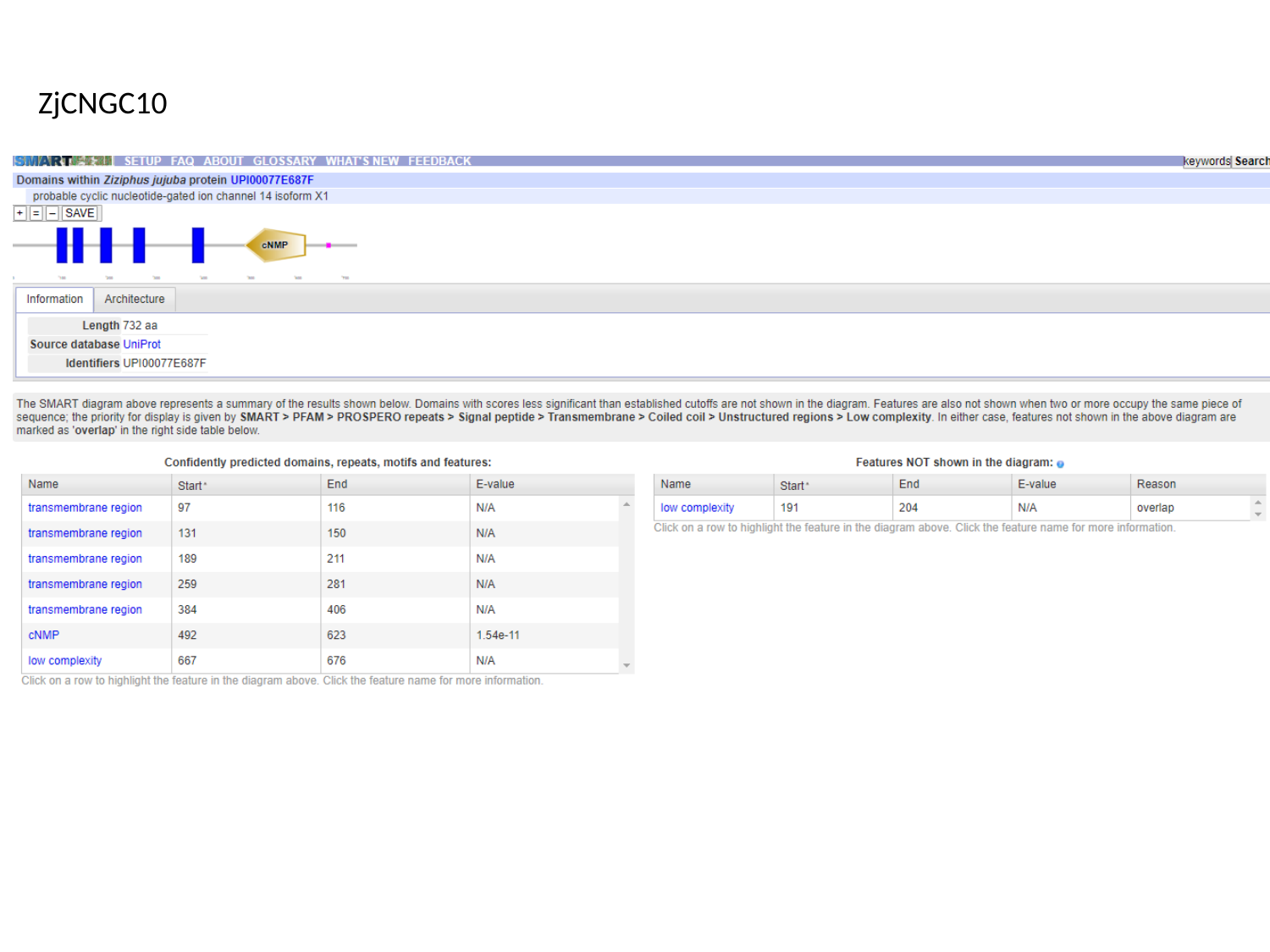

ZjCNGC10

## Slide 11
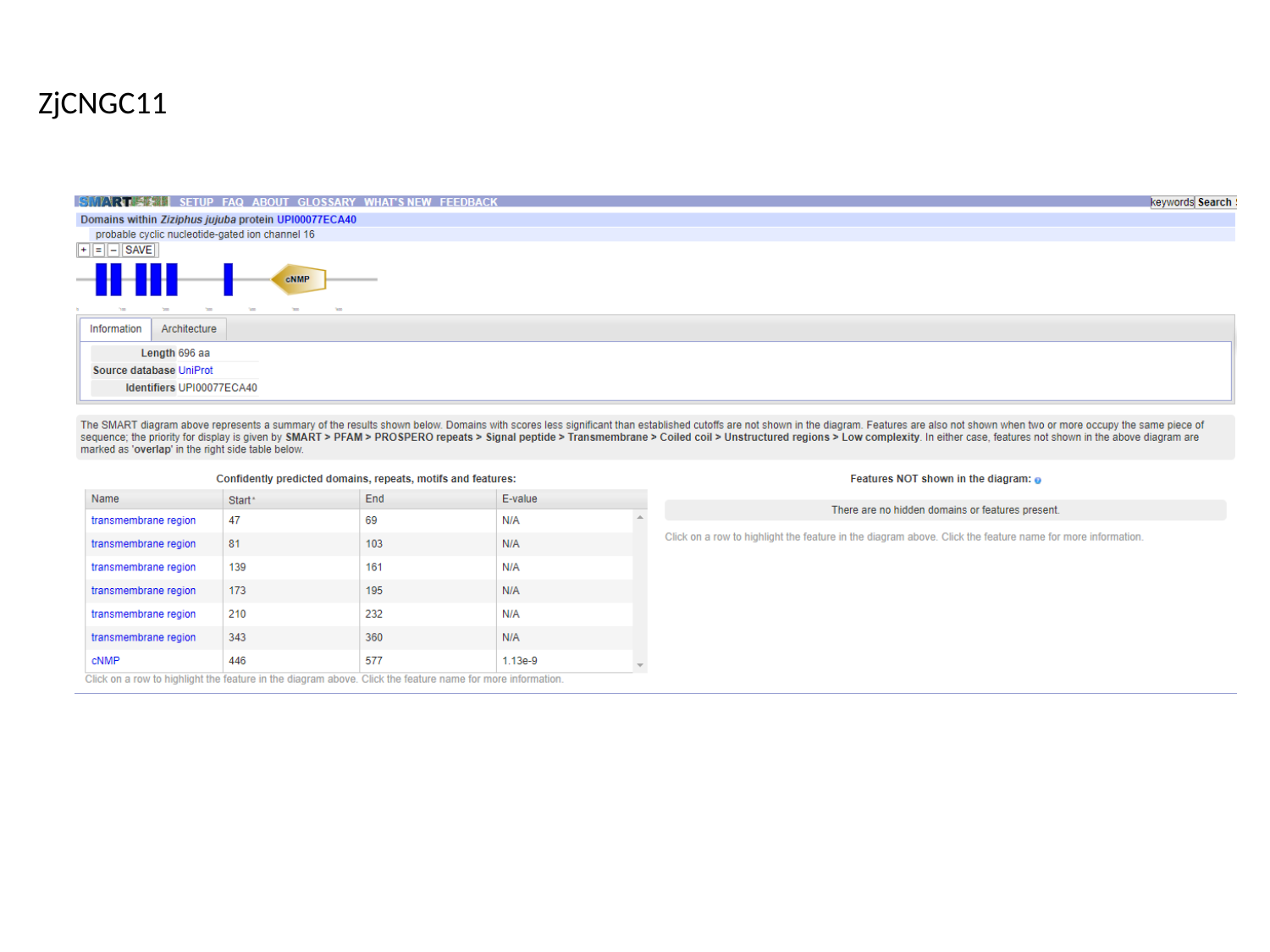

ZjCNGC11

## Slide 12
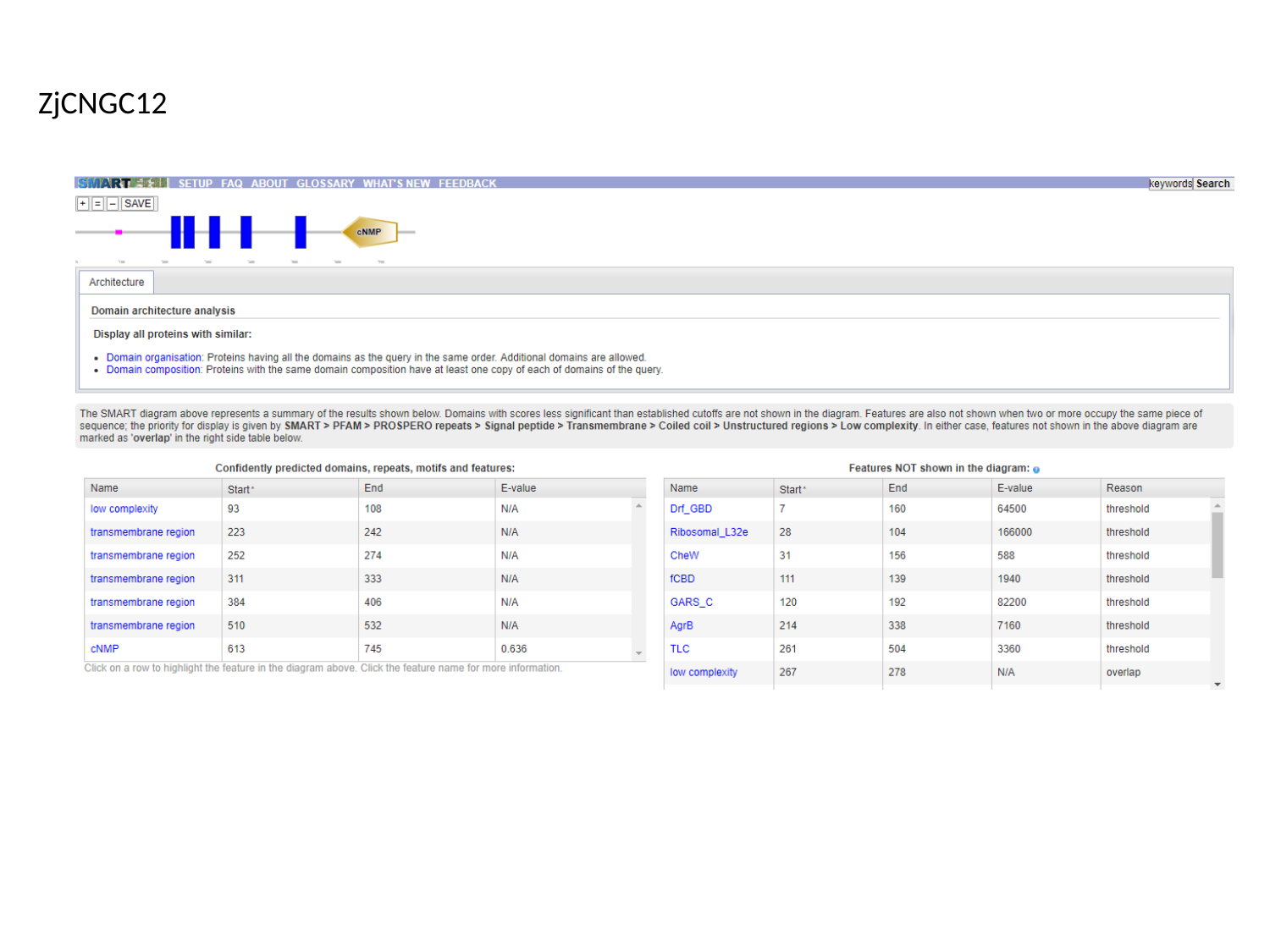

ZjCNGC12

## Slide 13
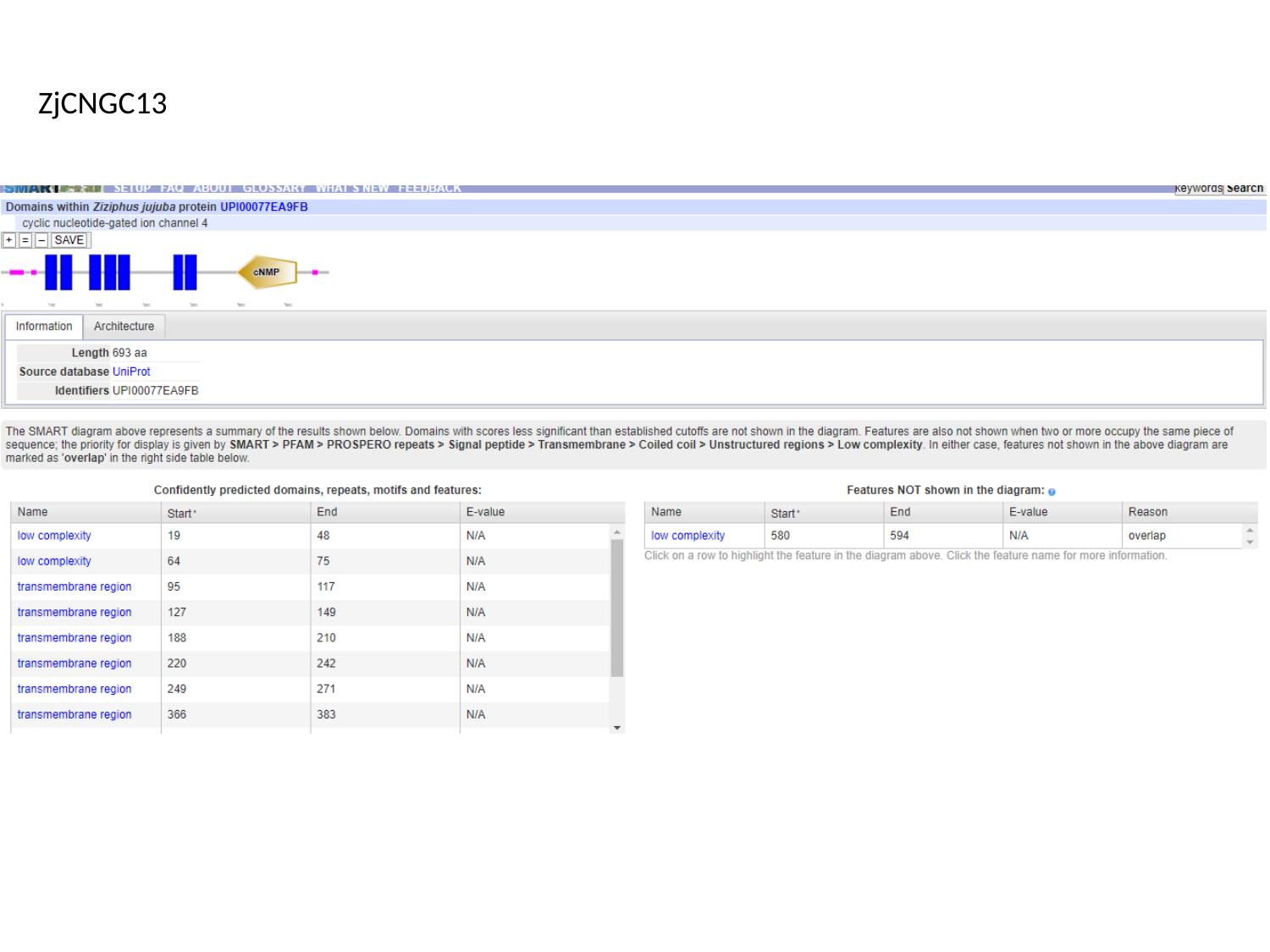

ZjCNGC13

## Slide 14
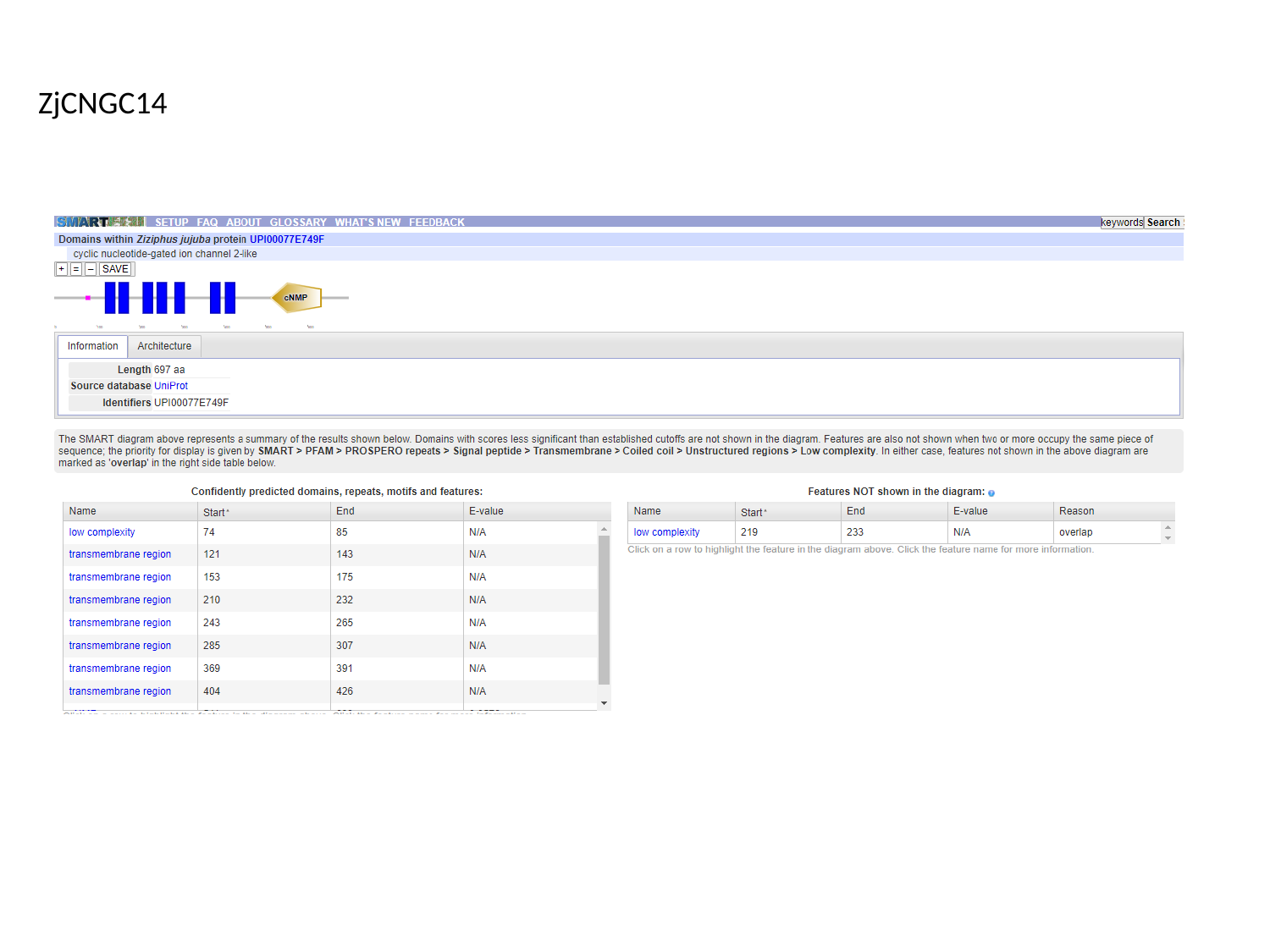

ZjCNGC14

## Slide 15
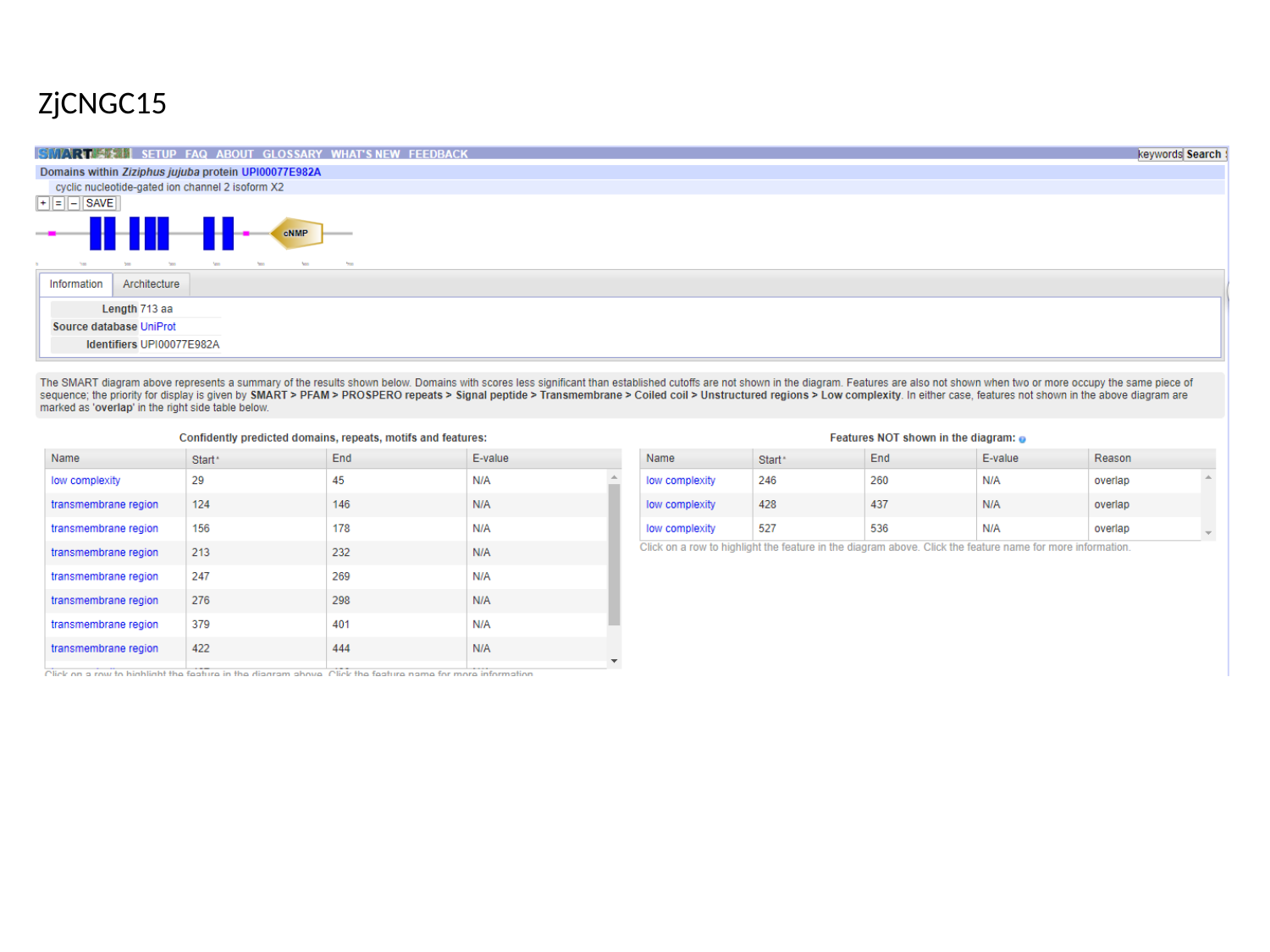

ZjCNGC15
